# Interaction of Adenosine, Modified Using Carborane Clusters, with Ovarian Cancer Cells: A New Anticancer Approach against Chemoresistance

**DOI:** 10.3390/cancers13153855

**Published:** 2021-07-30

**Authors:** Katarzyna Bednarska-Szczepaniak, Ewelina Przelazły, Katarzyna Dominika Kania, Marzena Szwed, Miroslava Litecká, Bohumír Grűner, Zbigniew J. Leśnikowski

**Affiliations:** 1 Laboratory of Medicinal Chemistry, Polish Academy of Sciences, Institute of Medical Biology, 106 Lodowa, 92-232 Lodz, Poland; sekretariat@cbm.pan.pl (E.P.); zlesnikowski@cbm.pan.pl (Z.J.L.); 2 Laboratory of Transcriptional Regulation, Polish Academy of Sciences, Institute of Medical Biology, 106 Lodowa, 92-232 Lodz, Poland; kkania@cbm.pan.pl; 3 Laboratory of Virology, Polish Academy of Sciences, Institute of Medical Biology, 106 Lodowa, 92-232 Lodz, Poland; 4 Department of Medical Biophysics, Institute of Biophysics, Faculty of Biology and Environmental Protection, University of Lodz, Pomorska 141/143, 90-236 Lodz, Poland; marzena.szwed@biol.uni.lodz.pl; 5 Institute of Inorganic Chemistry of the Czech Academy of Sciences, Hlavní 1001, 250 68 Rež, Czech Republic; litecka@iic.cas.cz (M.L.); gruner@iic.cas.cz (B.G.)

**Keywords:** ovarian cancer, chemoresistance, cancer spheroids, cisplatin, apoptosis, reactive oxygen species, nucleoside derivatives, metallacarboranes

## Abstract

**Simple Summary:**

Ovarian cancer has the highest mortality rate among gynecological malignancies and is the second most commonly diagnosed gynecological cancer. Acquired resistance to platinum therapy remains a major problem in gynecological oncology. Considering the unique physicochemical properties of the metallacarboranes and antimetabolite activity of nucleoside derivatives, we designed and synthesized the adenosine conjugates with metallacarboranes containing iron, cobalt, or chromium as semi-abiotic compounds that influence cisplatin-resistance of cancer cells. The iron-containing conjugate of metallacarborane and adenosine sensitized resistant cancer cells and highly resistant multicellular cancer spheroids to cisplatin, increasing cell cycle arrest, apoptosis or necrosis, and reactive oxygen species production. The presence of nucleosides in the structure of the conjugates was revealed to be indispensable for protecting cells against the development of cross-resistance to cisplatin, carboplatin, doxorubicin, paclitaxel, or gemcitabine in long-term-cultures. The findings indicate that adenine nucleoside modified with metallacarboranes may help sensitize ovarian cancer cells to chemotherapeutic agents in combination therapy.

**Abstract:**

Platinum compounds remain the first-line drugs for the treatment of most lethal gynecological malignancies and ovarian cancers. Acquired platinum resistance remains a major challenge in gynecological oncology. Considering the unique physicochemical properties of the metallacarboranes modifier and the significant role of nucleoside derivatives as anticancer antimetabolites, we designed and synthesized a set of adenosine conjugates with metallacarboranes containing iron, cobalt, or chromium as semi-abiotic compounds that influence the cisplatin sensitivity of ovarian cancer cells. Adherent cultures of ovarian carcinoma cell lines and multicellular spheroids, ranging from sensitive to highly resistant including experimental cell lines “not responding” to platinum drugs were used. Iron-containing metallacarborane conjugates showed the best anticancer activity, especially against resistant cells. Compound modified at the C2′ nucleoside position showed the best activity in resistant cancer cells and highly resistant cancer spheroids exposed to cisplatin, increasing cell cycle arrest, apoptosis or necrosis, and reactive oxygen species production. Moreover, it showed high cellular accumulation and did not induce cross-resistance to cisplatin, carboplatin, doxorubicin, paclitaxel, or gemcitabine in long-term cultures. The reference *nido*-carborane derivative (no metal ions) and unmodified nucleosides were not as effective. These findings indicate that metallacarborane modification of adenosine may sensitize ovarian cancer cells to cisplatin in combination treatment.

## 1. Introduction

Intrinsic drug resistance and acquired drug resistance are major causes of failure of therapy for ovarian cancer, the most deadly gynecologic malignancy [1,2]. Most patients who have ovarian cancer and are in the advanced stage of the disease respond well to platinum-based chemotherapy used postoperatively. However, the therapeutic effect is short-lived and relapse often occurs [3,4]. In ovarian cancer, the low effectiveness of chemotherapy results not only from the drug resistance of cancer cells, but also from the suppression of the antitumor activity of the immune system in the tumor microenvironment [5,6,7]. Adenosine inhibits the biological functions of various immune cells in the tumor environment by interacting with adenosine receptors located on the cell surface, mainly on leukocytes. Moreover, large amounts of adenosine monophosphate released from apoptotic cells induces the anti-inflammatory activity of macrophages through interaction with the adenosine receptor [8]. In this context, purinergic signaling is a first-line molecular target in personalized cancer treatment and is currently recognized as including novel checkpoint inhibitor targets [9,10,11]. Modified nucleosides, antimetabolites, have been previously considered in the design of anticancer drugs and recently, initial attempts have been made toward personalized therapy for ovarian cancer [12,13,14,15,16]. Modified nucleosides used in antitumor treatments have a good toxicity profile and do not damage non-hematopoietic tissues. Adenosine derivatives or analogs have limited efficacy in treating solid tumors; nevertheless, some of them are used as second-line drugs to support combination therapy for recurrent ovarian cancer. Although the concept of using adenosine derivatives as potential supportive drugs in cancer therapy targeting immune cells is well established, the use of cancer cells as a therapeutic target in the context of the purinergic system is a newly developing approach. In our recent study, we found that adenosine analogs may enhance the sensitivity of resistant ovarian cancer cells to cisplatin, acting in an additive or even synergistic manner [17].

One possible avenue in the search for innovative solutions to the design of bioactive compounds is the use of boron clusters as new 3D scaffolds and modifying units [18,19]. Twelve vertex *closo*-dicarbadodecaboranes(12) (C_2_B_10_H_12_), with frequently used trivial name carboranes, are the most commonly used type of boron clusters in biomedical research; they are cages with rigid icosahedral geometry (icosahedron) and are composed of boron, carbon, and hydrogen atoms. Carboranes are abiotic structures that have no counterparts in nature; they are chemically stable in living systems and resistant to enzymatic catalysis. *Meta* and *para* isomers of *closo*-dicarbaboranes may be degraded to form a *nido*-7,8-dicarbaundecaborate anion. The *nido*-7,8-dicarbaundecaborate anion undergoes insertion reactions with transition metals to form polyhedral double-cage complexes. The resulting structure is a sandwich compound consisting of two 11-vertex boron cluster ligands coordinated with a metal ion. Currently, the most frequently used semi-trivial name for this class of compounds is metalla bis(dicarbollide). The use of metalla bis(dicarbollides) for designing biologically active compounds has garnered increasing interest, primarily because of their extraordinary characteristic properties including their rigidity, three-dimensional ellipsoidal shape, stability in the biological environment, bio-orthogonality, and hydrophobicity. Metallacarboranes and carboranes have been identified as promising therapeutically important compounds [20,21,22,23,24] including boronated nucleosides that have been extensively studied in recent years [25,26,27,28]. Several approaches have been previously proposed by us for the synthesis of adenosine and 2′-deoxyadenosine derivatives modified with boron clusters (carboranes and metallacarboranes) [29,30,31,32,33,34,35,36,37,38].

In our current study, we designed, synthesized, and evaluated some carborane modifications of adenosine and 2′-deoxyadenosine with biological activity that inhibit the respiratory burst of activated neutrophils and inhibit aggregation of blood platelets [30]. In this direction, a series of already published as well as newly synthesized derivatives, that is, adenosine- and 2′-deoxyadenosine-metallacarborane conjugates bearing metallacarboranes with various metals and at different locations within the nucleoside, were obtained for the purpose of the current study, that is, for assessment as anticancer compounds.

To obtain derivatives of adenosine and 2′-deoxyadenosine, four different boron clusters were used: three types of sandwich complexes corresponding to *closo*-3,3′-*commo*-metalla-bis(1,2-dicarbaundecaborate) [M(C_2_B_9_H_11_)_2_] ions (abbreviated name: 3-metalla-bis(1,2-dicarba-closo-undecaborate](−1)), containing iron (Fe), chromium (Cr), or cobalt (Co) ions; and *nido*-7,8-dicarbaundecaborate ([C_2_B_9_H_11_](1-) ion as a reference compound. The cesium salts of these synthesized anions were converted into their corresponding sodium salts through metathesis and used in this study. The hybrid compounds obtained, consisting of an abiotic (metallacarborane) and organic moiety (adenine nucleoside), are expected to exhibit features of multifunctional compounds interacting with specific cellular targets of cancer cells. In the current study, the anticancer activity of the adenine nucleoside conjugates bearing metallacarboranes was evaluated in ovarian carcinoma cell lines that differed in cisplatin sensitivity, ranging from high sensitivity to high refractoriness. Herein, we determined the short- and long-time effects of adenosine analogs modified with different carboranyl clusters, directly or in combination with cisplatin, on ovarian cancer cells including cancer cell spheroids. The toxicity of the compounds and the relationship between toxicity, cell cycle arrest, and cellular accumulation as well as the relationship between phases of cell cycle and reactive oxygen species (ROS) formation were evaluated to assess the nature of the activities of the compounds.

## 2. Results

### 2.1. Principle of the Study

**Scheme 1:**
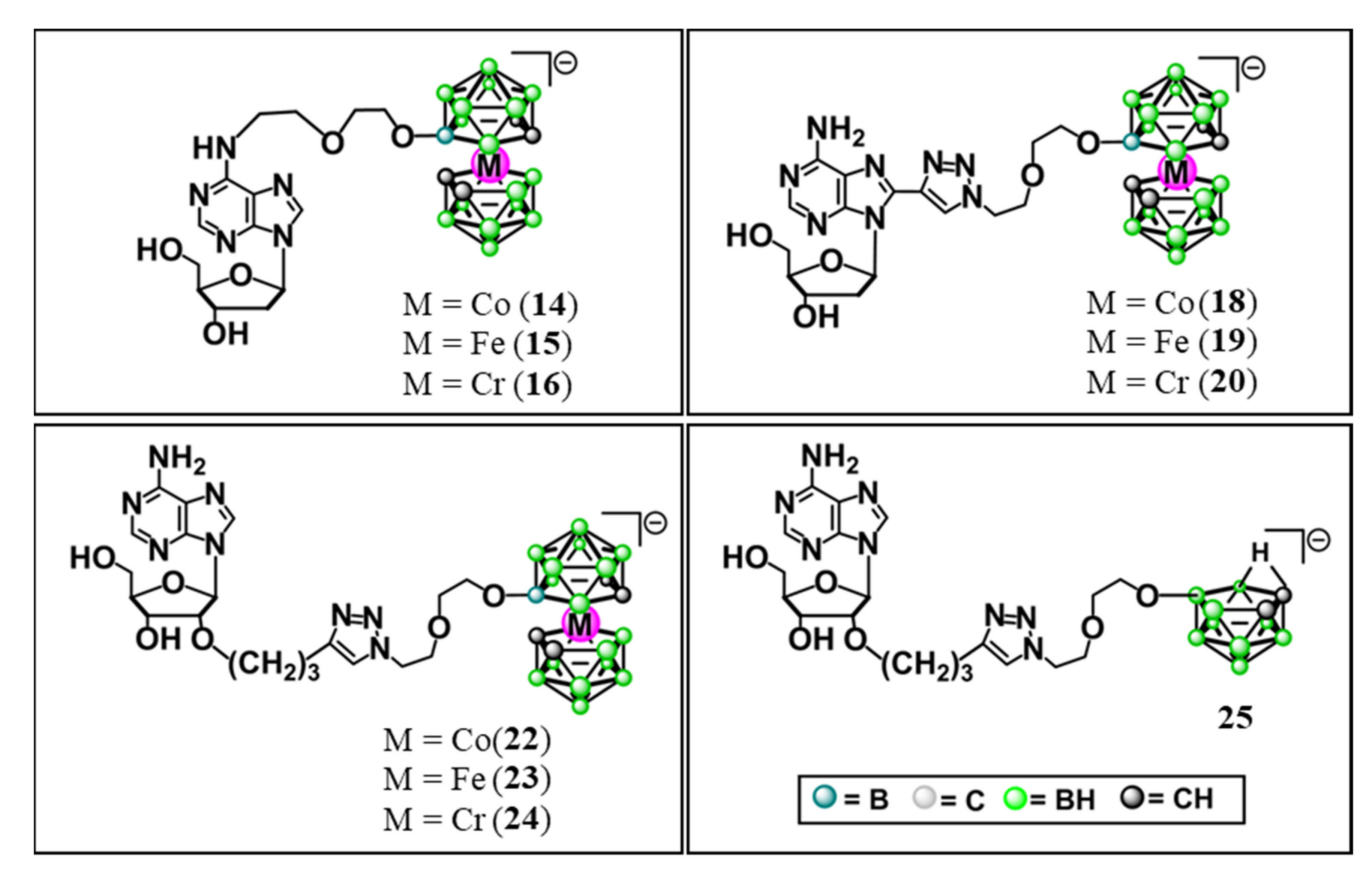
Adenine nucleosides modified with metallacarboranes and *nido*-carborane; 6-*N*-{5-[3,3′-metalla-bis(1,2-dicarba-*closo*-undekaborate)-8-yl]-3-oxa-pentoxy}-2′-deoxyadenosine (**14**, **15**, **16**); 8-{5-[3,3′-metalla-bis(1,2-dicarba-*closo*-undekaborate)-8-yl]-3-oxa-pentoxy}-1*N*-1,2,3-triazol-4-yl}-2′-deoxyadenosine (**18**, **19**, **20**); 2′-*O*-{{5-[3,3′-metalla-bis(1,2-dicarba-*closo*-undekaborate)-8-yl]-3-oxa-pentoxy}-1*N*-1,2,3-triazol-4-yl}propyloadenosine (**22**, **23**, **24**); 2′-*O*-{[5-(7,8-dicarba-*nido*-undekaborate-10-yl)-3-oxa-pentoxy]-1*N*-1,2,3-triazol-4-yl}propyloadenosine (**25**). The compounds were used in the form of their respective sodium salts.

The following set of nine Co-, Fe-, or Cr-containing metalla bis(dicarbollide) derivatives of adenine nucleosides and one reference compound without metal ions were synthesized and evaluated against ovarian cancer cells (Scheme 1):

- modifications at the N^6^ position of purine, compounds **14**, **15**, and **16**;
- modification at the C8 of the nucleobase, compounds **18**, **19**, and **20**; and
- modification at the C2′ position of the ribose moiety, compounds **22**, **23**, and **24**.

Moreover, adenosine derivative with *nido*-7,8-dicarbaundecaborate, modified at the C2′ position of the ribose moiety without metal ions was synthesized and examined as the reference compound (compound **25**, Scheme 1).

Unmodified adenosine (Ade), or 2′-deoxyadenosine (dAde), and metalla bis(dicarbollide) modifying units itself (compounds **1**–**3**) were reference compounds in relation to the presence of the nucleoside or metalla bis(dicarbollide) unit:

- 3-cobalt-bis(1,2-dicarba-*closo*-undecaborate](−1) (**3**) (**1**),
- 3-ferra-bis(1,2-dicarba-*closo*-undecaborate](−1) (**2**),
- 3-chromma-bis(1,2-dicarba-*closo*-undecaborate](−1) (**3**).

The preparation of metallacarborane- and *nido*-7,8-dicarbaundecaborate derivatives is described at the end of this subsection (Section 2.11, Preparation of Metallacarborane- and *Nido*-7,8-Dicarbaundecaborate Derivatives). The x-ray structures obtained for the parent chromium sandwich **3** and its dioxane derivatives are also presented.

**Figure 1:**
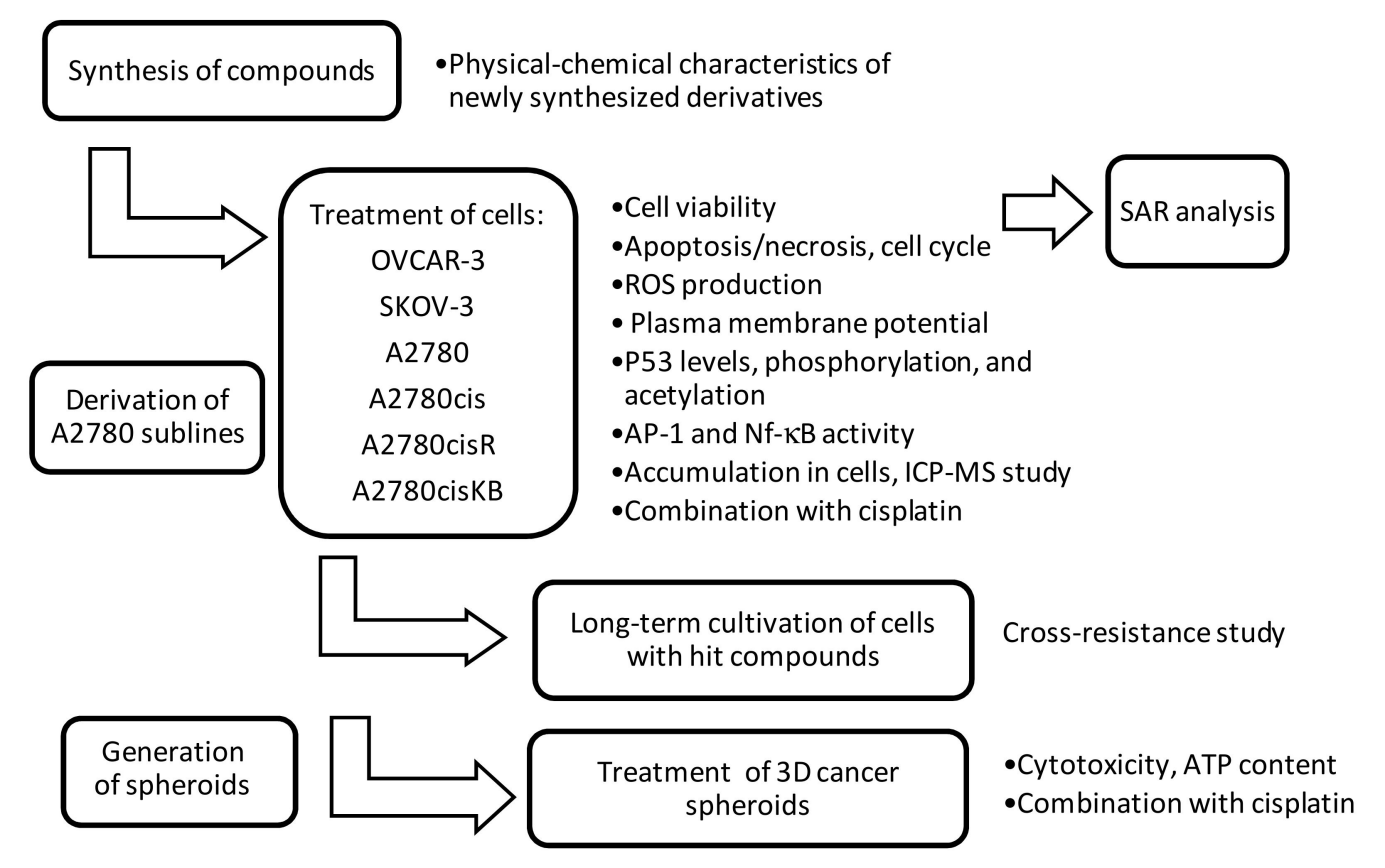
Principle of the current study. SAR—structure-activity relationship; ROS—reactive oxygen species; p53—tumor protein p53; AP-1—activator protein 1, transcription factor; Nf-κB—nuclear factor kappa-light-chain-enhancer; ICP-MS—inductively coupled plasma mass spectrometry; ATP—adenosine triphosphates.

Anticancer activities of adenine nucleoside modifications with metalla bis(dicarbollides) were evaluated using ovarian carcinoma cell lines that differed in cisplatin sensitivity, ranging from high sensitivity to high refractoriness: sensitive (A2780 and OVCAR-3) > moderately resistant (A2780cis) > resistant (A2780cisR and SKOV-3) > highly resistant (A2780cisKB). Two cell culture models, adherent monolayer and 3D spheroids, were used. Moreover, long-term cell cultures, in addition to the standard 24–48 h assays, were applied to evaluate the possibility of acquired drug resistance. In basic experiments, the compounds were used alone, while in cell sensitization studies, they were used in combination with cisplatin. For long-term treatment, the selected compounds were added to the culture medium and used to culture the cells, which were then treated with five standard anticancer drugs (cisplatin, carboplatin, doxorubicin, paclitaxel, and gemcitabine). The flow chart of the study is shown in Figure 1.

The study was performed using six cell lines with various levels of sensitivity to cisplatin including commercial cell lines with differing sensitivities to platinum drugs (A2780, A2780cis, SKOV-3, and OVCAR-3) as well as experimental cell lines obtained in our laboratory (A2780cisR and A2780cisKB), which are highly resistant to platinum drugs. The experimental cell lines were obtained using long-term cultures of A2780cis cells with successively increasing concentrations of cisplatin (5–15 μM, as described in the Section 4). The two resulting cell lines, A2780cisR and A2780cisKB, which were further characterized by short tandem repeat (STR) analysis, showed enhanced resistance to cisplatin and cross-resistance to carboplatin. The IC50 values of cisplatin and carboplatin for the cell lines, together with the resistance index values, are summarized in Table 1.

The A2780 cell line is a parental, cisplatin-sensitive cell line. A2780cis, A2780cisR, and A2780cisKB are cells with increased resistance to cisplatin and carboplatin. These cells were completely resistant to carboplatin (IC50 >100 μM) and 13 times more resistant to cisplatin than the parental A2780 cell line, which is sensitive to this drug (IC50, 2.8 ± 0.21 μM). The resistance of SKOV-3 cells to cisplatin was similar to that of A2780cisKB cells and the sensitivity to carboplatin was similar to that of A2780cisR, whereas platinum sensitivity of OVCAR-3 cells was relatively high (IC50 5.1 ± 0.45 μM, Table 1). 

Originally, the OVCAR-3 cell line was derived from progressive ovarian adenocarcinoma. Although the OVCAR-3 cell line is considered chemoresistant [39], practically, its sensitivity to cisplatin in vitro is comparable to that of the “sensitive” cell line A2780 (5.1 ± 0.45 μM versus IC50 2.8 ± 0.21 μM, respectively, Table 1). Therefore, we classified the OVCAR-3 cells as relatively cisplatin-sensitive in our study. This in vitro phenomenon in OVCAR-3 cells has been previously reported by others [40]. 

### 2.2. Cytotoxicity of the Compounds in Relation to the Resistance of the Cells to Cisplatin

**Figure 2:**
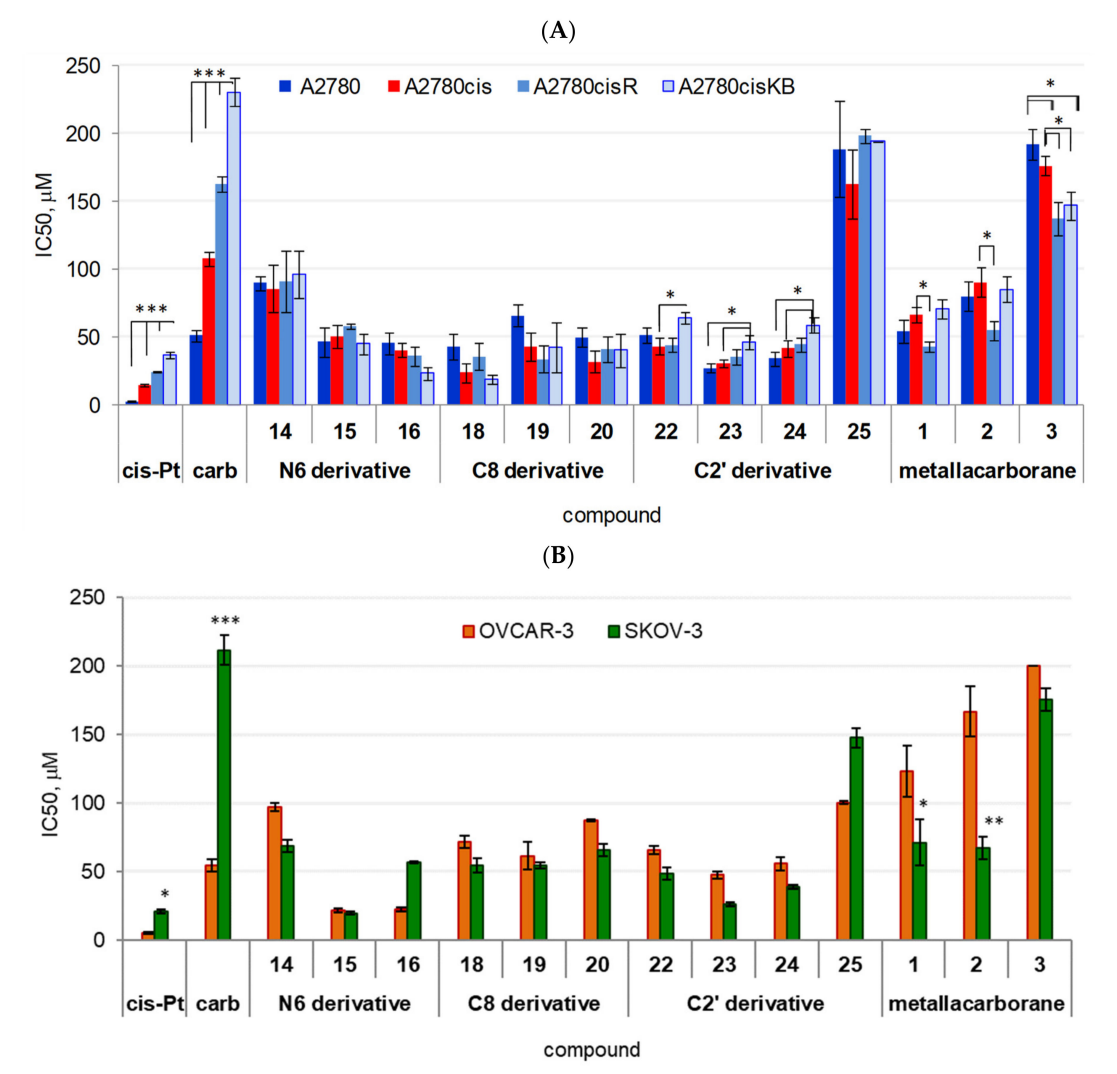
Effect of adenosine and 2′-deoxyadenosine modified with metalla bis(dicarbollides) containing Co, Fe, or Cr (compounds 14, 15, 16, 18, 19, 20, 22, 23, and 24, IC50 [μM]) on cancer cell viability, (**A**) A2780 cell lines, (**B**) SKOV-3 and OVCAR-3 cell lines. Unmodified metalla bis(dicarbollides) (compounds 1, 2, and 3) and compound 25 were used as reference compounds; effects of cisplatin and carboplatin are shown for comparison. Data are expressed in relative fluorescence units (MFI, median fluorescence intensity); the mean ± SEM values were calculated for n ≥ 6 independent experiments. * *p* < 0.05, ** *p* < 0.01, and *** *p* < 0.001, differences between cell lines (ANOVA, Bonferroni post-hoc comparison). Compound 25, not having a metal ion cluster, was not effective (IC50 ≥150 μM for A2780 lines and SKOV-3 cells; IC50 100 μM for OVCAR-3 cells, Figure 2A,B). Adenosine and 2′-deoxyadenosine used as reference compounds were not cytotoxic (data not shown).

When the cytotoxicity of the metallacarborane adenosine conjugates was compared by adding the investigated compounds at increasing concentrations to a panel of cancer cell lines, a striking difference was observed in the cytotoxicity of the metallacarborane adenosine derivatives. The cells were incubated for 48 h with the compounds (0.01–200 μM). The activity of the compounds was analyzed within and between cell lines differing in sensitivity to cisplatin. Cell viability was evaluated using a neutral red assay (see the Section 4). A comparison of the IC50 values is shown in Figure 2A,B.

#### 2.2.1. Sensitive A2780 Cell Line

In general, adenine nucleosides modified with metallacarboranes significantly decreased cell viability, regardless of cisplatin resistance. The cytotoxicity of the compounds (IC50 values) was relatively comparable across all A2780 sublines ranging from sensitive (A2780) to highly resistant (A2780cisKB) cells, except that of compound **14**, bearing metallacaborane with Co ion at the N^6^ position, which was the least cytotoxic (Figure 2A). The IC50 values of compound **14** were comparable to those of carboplatin for A2780cis cells (IC50 90 ± 4.9 μM, A2780; 85 ± 18 μM, A2780cis; 91 ± 23 μM, A2780cisR; and 96 ± 18 μM, A2780cisKB, *p* < 0.001 *versus* other compounds, ANOVA). The IC50 values of compounds **15**–**23** ranged from 27 ± 3.6 μM (compound **23**) to 66 ± 7.9 μM for sensitive A2780 cells (compound **19**), and they were significantly higher than that of cisplatin IC50 (2.8 ± 0.21 μM, *p* < 0.00001), but comparable to carboplatin IC50 (51 ± 4.4 μM) (*post-hoc* Bonferroni comparisons). The range of IC50 values of the compounds for resistant A2780cis cells was similar to that for A2780 cells with values from 24 ± 7.2 μM (compound **18**) to 51 ± 8.7 μM (compound **15**). 

#### 2.2.2. Resistant A2780cis Cell Line and Highly Resistant A2780cisR and A2780cisKB Cells

The IC50 values of the compounds for A2780cis cells were still higher than those of cisplatin (15 ± 0.64 μM), but considerably lower than those of carboplatin (108 ± 4.9 μM, *p* < 0.001, *post-hoc* Bonferroni comparisons). The toxicity of compounds **16**, **18**, **19**, and **23** for A2780cisR cells, and compounds **15** and **23** for A2780cisKB cells was comparable to that of cisplatin and higher than that of carboplatin (*p* < 0.00001). Significant differences were found across cell lines for the effects of compounds **22**, **23**, and **24**; the IC50 values of these compounds increased slightly with increasing cell resistance to cisplatin (*p* < 0.05; *post-hoc* Bonferroni comparisons, Figure 2A).

#### 2.2.3. OVCAR-3 and SKOV-3 Cell Lines 

Similar to the A2780 sublines, the sensitivity of OVCAR-3 and SKOV-3 cells to the nucleoside derivatives was independent of cisplatin resistance (Figure 2B). In general, the toxicity of the compounds for SKOV-3 cells was lower than that of cisplatin, except for two derivatives with Fe ions (compounds **15** and **23**), which were as active as cisplatin (IC50 respectively, 19 ± 0.9 μM and 26 ± 1.6 μM *versus* 21 ± 1.6 μM, Figure 2B). In contrast, all nucleoside derivatives were more potent than carboplatin against SKOV-3 cells (*p* < 0.0001). The nucleoside derivatives were less effective on the viability of OVCAR-3 cells than cisplatin and less toxic than carboplatin, except for compounds **15** and **16** (N^6^ modifications with Fe and Cr ions) that were considerably more toxic than carboplatin (*p* < 0.05, *post-hoc* Bonferroni comparisons). In contrast, a high sensitivity to compound **15** was a distinguishing feature between OVCAR-3 or SKOV-3 and other cells.

### 2.3. Structure–Activity Relationship. The Presence of Nucleoside, Role of Metal Ions, and Substitution Site

#### 2.3.1. Role of Nucleoside and Metal Ion

The in-depth SAR analysis showed that the presence of nucleoside qualified (Cr derivatives), enhanced (Fe derivatives), or differentiated (Co derivatives) the cytotoxicity of these compounds.

Cr-containing compounds: The cytotoxicity of the metallacarboranes depended on whether they were bound to the nucleosides (*p* < 0.0001, ANOVA), and it was confirmed for all cell lines and almost all the compounds, especially those containing Cr ions (**3**, **16**, **20**, and **24**). Unmodified, Cr-containing metalla bis(dicarbollide) (compound **3**) was practically nontoxic to A2780, A2780cis, SKOV-3, and OVCAR-3 cells (IC50 >150 μM) and less toxic to the highly resistant A2780cisR and A2780cisKB cells (IC50 up to 150 μM, *p* < 0.05, Figure 2A,B). The adenine nucleosides modified with this Cr-containing bis(dicarbollide) cage (compounds **16**, **20**, and **24**), regardless of the substitution site, were significantly toxic to all cell lines (Figure 2A,B).

Co-containing compounds: In contrast to Cr, Co-containing bis(dicarbollide) (compound **1**) showed significant toxicity to all A2780 sublines and SKOV-3 cells, among which the resistant A2780cisR cells showed the highest sensitivity to this compound (IC50 43 ± 3.7 μM, *p* < 0.05, Figure 2A,B). The 2′-deoxyadenosine modification at the N^6^ position in Co-containing metallacarborane (compound **14**) resulted in a loss of toxicity to A2780 (*p* < 0.05), A2780cis (*p* < 0.05), and A2780cisR cells (*p* < 0.01, increase in IC50, Figure 2B), while substitution at the C8 position (compound **18**) increased toxicity to resistant A2780cis (*p* < 0.01) and A2780cisKB (*p* < 0.05) cells, in comparison to the unmodified parent cage; substitution at the C2′ position of adenosine (compound **22**) did not significantly change toxicity (*p* > 0.05, Figure 2A). Nucleoside modification with cobalta bis(dicarbollide) did not significantly alter the toxicity against both SKOV-3 and OVCAR-3 cells with respect to the unmodified metallacarborane (compound **1)**, regardless of whether the metallacarborane was toxic to cells (SKOV-3) or not (OVCAR-3, Figure 2B).

Fe-containing compounds: Ferra bis(dicarbollide) (compound **2**) significantly decreased the viability of A2780 sublines and SKOV-3 cells, as did compound **1**; the IC50 values ranged from 55 ± 7.3 μM (for A2780cisR cells) to 90 ± 11 μM (for A2780cis cells, *p* < 0.05, Figure 2A,B). The toxicity of 2′-deoxyadenosine modification at the N^6^ position with ferra bis(dicarbollide) (compound **15**) was similar to that of the unmodified parent compound **2** for almost all A2780 sublines; however, for A2780cis cells, compound **15** was twice as toxic as compound **2** (*p* < 0.01, Figure 2A). Compound **15** showed high toxicity to SKOV-3 and OVCAR-3, that is, 3.5 and 8 times higher than the toxicity of the unmodified compound **2** (*p* < 0.0001 and *p* < 0.001, respectively). The modification at the C8 position of the nucleoside (compound **19**) was less effective on cell viability than the unmodified cage, and significant differences were found only for A2780cis (*p* < 0.01) and OVCAR-3 cells (*p* < 0.05). Substitution of the adenosine at the C2′ position with ferra bis(dicarbollide) (compound **23**) was the most efficient modification in relation to all the cell lines for which this compound was significantly more toxic than the unmodified cage. The IC50 values for compound **23** ranged from 27 ± 3.6 μM (A2780 cells) to 46 ± 5.2 μM (A2780cisKB cells), which were 2–3.5 times lower than that of the unmodified ferra bis(dicarbollide) (A2780, *p* < 0.01; A2780cis, *p* < 0.001; A2780cisR, *p* < 0.05; A2780cisKB, *p* < 0.05; SKOV-3, *p* < 0.05; and OVCAR-3, *p* < 0.01).

**Figure 3:**
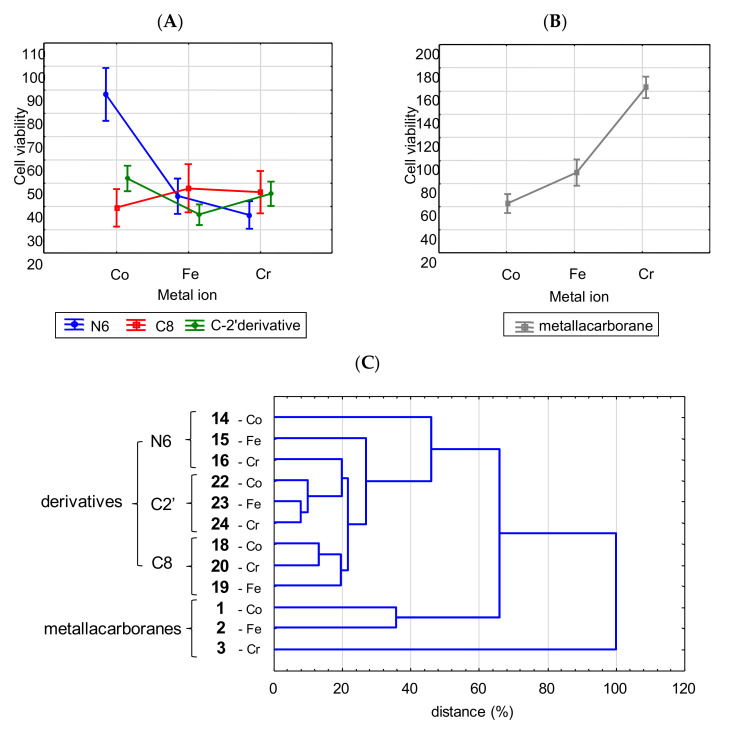
Activity of adenosine derivatives modified with metallacarboranes in relation to metal type and substitution site (**A**); (**B**) the significance of metal ion for the activity of unmodified metalla bis(dicarbollide) ions. Data were weighted arithmetic averages ± CI 0.95 calculated for a given compound (IC50, μM) across the cancer cell lines (factor analysis). In the case of the unmodified metalla bis(dicarbollide) ions and the N^6^ derivative with Co ion (compound **14**), the dependence between cytotoxicity and type of metal is clearly visible. Contrary to the latter, the diversity of activity of the remaining derivatives was not as spectacular, although significant (*p* < 0.05, Co- or Cr- *versus* Fe-derivatives). (**C**) The result of cluster analysis of twelve compounds, based on their toxicity against ovarian cancer cells. Compounds **14**–**16**, **18**–**20**, and **22**–**24** are adenine nucleosides modified at the N^6^, C2′, and C8 positions with Co-, Fe-, and Cr-containing bis(dicarbollide) ions; compounds **1**–**3** are unmodified parent cages; unweighted pair-group method with arithmetic mean (UPGMA), Euclidean distance.

The cytotoxicity of the nucleoside derivatives also depended on both the substitution site (Figure 3A) within the adenosine scaffold for all the cell lines (*p* = 0.032) and the metal ion (*p* < 0.0001, Figure 3B), and these two factors were interrelated (*p* < 0.0001, factorial ANOVA).

#### 2.3.2. Role of Substitution Site

N^6^ substitution: Among the N^6^ derivatives, the presence of Co ions (compound **14**) resulted in a 2–4-fold decrease in cytotoxicity for all cell lines in comparison to those of the analogous compounds containing Fe or Cr ions (Figure 3A); compound **15**, *p* < 0.001; and **16**, *p* < 0.00001. The effect of N^6^ derivatives containing Fe or Cr ions (compounds **15** and **16**) on cell viability was comparable for all cell lines except SKOV-3, which showed very high sensitivity to compound **15** than to compound **16** (*p* < 0.001, see Figure 2B). C8 and C2′ substitutions: The effect of the C8 derivative on cell viability (compounds **18**, **19**, and **20**) was independent of the type of metal ion (Figure 3A). Although the C2′ derivative modified with ferra bis(dicarbollide) (compound **23**) showed a 1.4–2-fold increase in cytotoxicity when compared to that of their Co-containing counterpart (Figure 2A,B and Figure 3A, compound **22**, A2780 cells, *p* < 0.05; OVCAR-3 cells, *p* < 0.01; and SKOV-3 cells, *p* < 0.05), all C2′ derivatives displayed significantly higher cytotoxicity than their *nido*-7,8-dicarbaundecaborate counterpart (compound **25**). Compound **25**, which did not have a metal ion cluster, did not affect cell viability in the range of the concentrations used (IC50 ≥ 150 μM for A2780 sublines and SKOV-3 cells; IC50 > 100 μM for OVCAR-3 cells, see Figure 2A,B). Compound **25** provided a clear example of the structure–activity relationship, showing the important role of sandwich-type *closo*-metalla bis(dicarbollide) ion *versus* free *nido*-7,8-dicarbaundecaborate ligand, which is almost nontoxic.

Hierarchical cluster analysis was used to characterize the diversity of compound potency against ovarian cancer cells (Figure 3C). UPGMA analysis is described in detail in the Supplementary Materials (Methodology details—UPGMA analysis).

Such a gathering showed that the presence of adenine nucleoside conjugated with metalla bis(dicarbollide) determines their effectiveness against cancer cells, and modifications at the C8 and C2′ positions may establish conditions for anticancer activity regardless of the metal ion within the metallacarborane. Modifications at the N^6^ position were active when Fe and Cr ions were present. The compounds affected cancer cell viability in the following order of potency: C2′ derivatives (**22**, **23**, **24**) > C8 derivatives (**18**, **19**, **20**) >> parent metalla bis(dicarbollides) **1**, **2** >> parent **3**; and N^6^ derivatives summarized separately: **16** >**15** >>**14**.

### 2.4. Accumulation of the Compounds in the Cells

**Figure 4:**
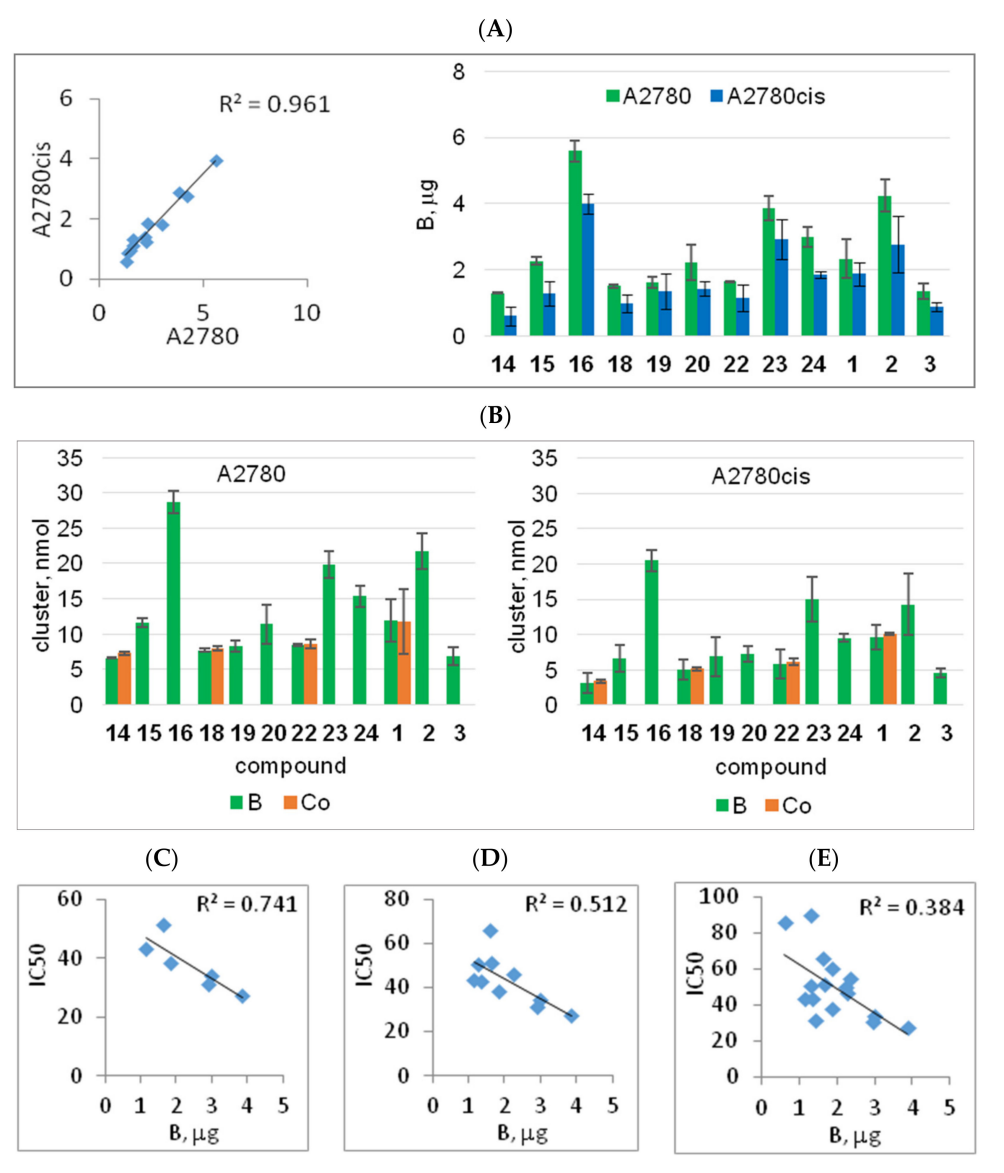
Incorporation of metalla bis(dicarbollide)-modified adenine nucleosides into A2780 and A2780cis cells (**A**,**B**) and its relationship to the cytotoxicity of the compounds (**C**–**E**). The amount of boron-bearing compounds in the cells was determined using ICP-MS, and it was expressed as μg B per 10^6^ cells (**A**) and nmol of compound per 10^6^ cells (**B**); the amount of the compounds was calculated for the B-containing metalla bis(dicarbollide) cluster [M(III)(1,2-C_2_B_9_H_11_)_2_]. Accumulation of Co-containing compounds (**14**, **18**, **22**, and **1**) was also determined based on Co content for comparison (orange bars). (**C**–**E**) Boron content in A2780cis cells in relation to compound cytotoxicity (IC50, μM); a stepwise analysis: C2′ derivatives (R^2^ = 0.741, *p* = 0.028, (**C**)); C2′ derivatives and compounds with Fe ions (R^2^ = 0.512, *p* = 0.020, (**D**)); all compounds **14**, **20**, and **1** (R^2^ = 0.384, *p* = 0.010, (**E**)); R^2^, Pearson correlation coefficient values.

The intracellular levels of the compounds were measured using inductively coupled plasma mass spectrometry (ICP-MS) in both cisplatin-sensitive and cisplatin-resistant cell lines in terms of B content in whole-cell lysates, as described in the Materials and Methods (Section 4) and expressed as micrograms of B or nmol of a compound in 1 × 10^9^ cells. A similar B accumulation pattern was found for compounds in both cell lines A2780 and A2780cis (R^2^ = 0.961; *p* < 0.00001, Figure 4A left); however, it was 30% lower in cisplatin-resistant cells than in the sensitive cells (Figure 4A right and Figure 4B).

The compounds accumulated in the cisplatin-resistant cells 30% lower than that in the sensitive cells, similar to cisplatin. Cellular accumulation of the compounds expressed in nM concentrations, calculated from B content, was compared to cisplatin accumulation (measured as Pt accumulation).

The compounds accumulated in A2780 cells in a range of 2.64–11.49 nmol, which was 2–10 times higher than the accumulation of cisplatin (1.10 ± 0.13 nmol, data not shown). In A2780cis, as in A2780, the compound accumulation (1.49–8.21 nmol) was distinctly above the cisplatin amount (0.75 ± 0.044 nmol, data not shown). Comparing the accumulation of the compounds and platinum showed that all the compounds entered the cells equally well and some in even greater amounts than that of platinum.

The ability of the compounds to accumulate in A2780cis cancer cells was closely related to their cytotoxicity levels (Figure 4C–E). However, this relationship was not observed for cisplatin-sensitive cells (data not shown). The analysis, which was performed in a stepwise manner, showed a significant relationship between compound cytotoxicity and their accumulation for C2′ derivatives (**22**, **23**, and **24**), compounds **15** and **19** (containing Fe ion), compounds **14** and **20**, and cobalta bis(dicarbollide) **1** (Figure 4C–E). Among them, compound **23** showed the highest accumulation, and compound **14** showed the lowest accumulation, which corresponded to their toxicity potential. The relationship between compound accumulation and cytotoxicity was not observed for derivatives **16** and **18** and unmodified metalla bis(dicarbollides), **2** and **3**. The amount of compound **16** in the cells was twice as high as that of the other Cr-containing derivatives with comparable cytotoxicity. Conversely, compound **18**, which was also significantly cytotoxic, accumulated in the cells at lower levels (Figure 4B). The unmodified Cr-containing bis(dicarbollide) (**3**), although non-active, was incorporated into the cells at low amounts (Figure 4B). Ferra bis(dicarbollide) **2** was taken up by the cells in twice the amount of cobalt bis(dicarbollide) **1** (Figure 4B), but it was considerably less effective on cell viability.

### 2.5. Apoptosis, Necrosis, and Reactive Oxygen Species (ROS) Production

**Figure 5:**
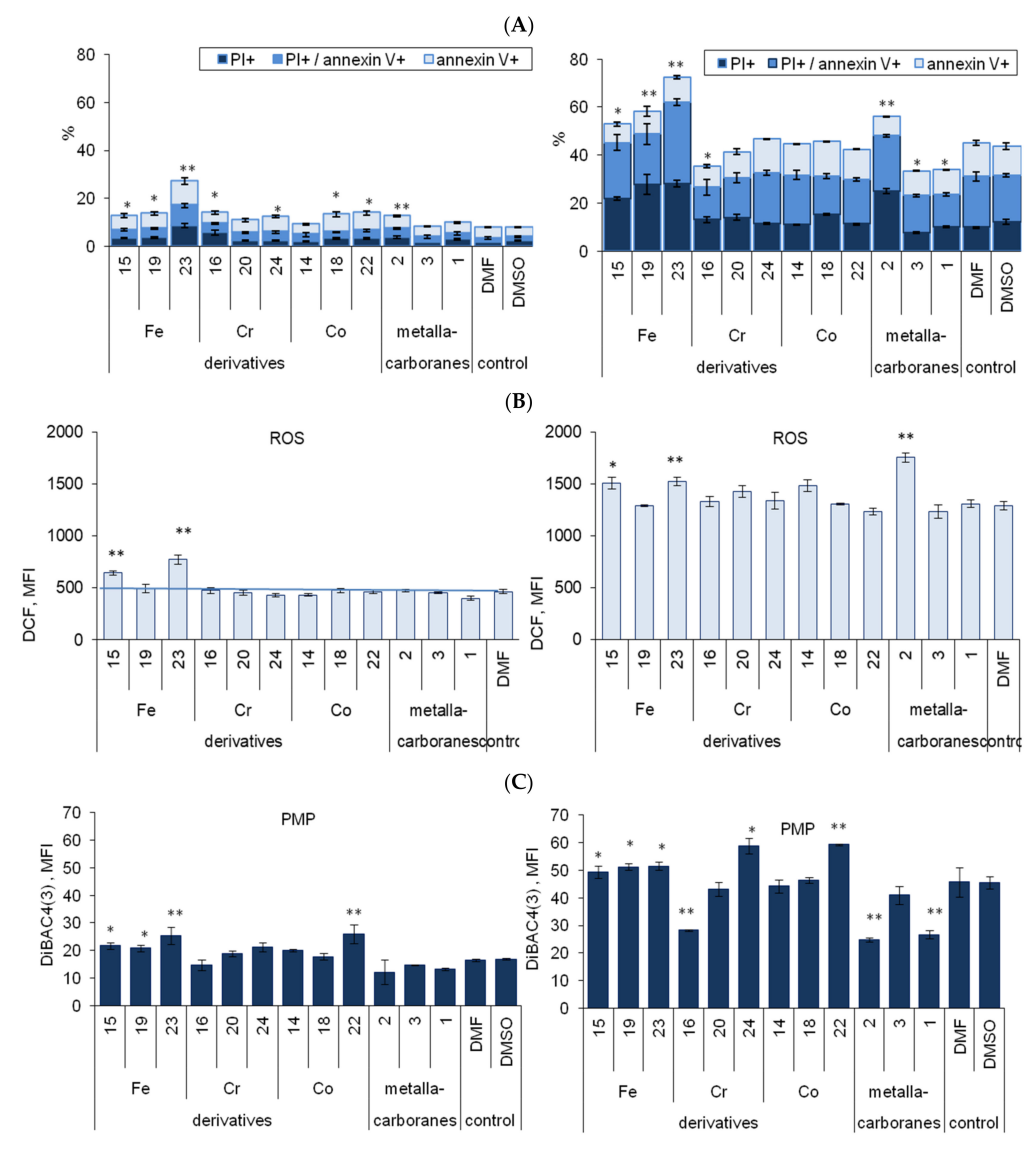
Effect of compounds **1**–**24** alone (40 μM) and in combination with cisplatin (10 μM) on the apoptosis and necrosis in A2780cis cells (**A**), ROS generation (**B**), and plasma membrane potential (**C**). N-dimethylformamide (DMF) or dimethyl sulfoxide (DMSO) (<0.04%, not toxic) was used as the control. Data are the mean of three to six independent experiments ± SEM; * *p* < 0.05; ** *p* < 0.01, compound *versus* without compound, Student t-test with Bonferroni correction. (**A**) Data were expressed as cumulative percentage of apoptotic and necrotic cells at different stages: necrosis (PI+, annexin V−), late (annexin V+/PI+), and early apoptosis (annexin V+/PI−); *p* values for nonapoptotic cells are shown, a detailed statistical analysis for necrosis and apoptosis is in the Supplementary Materials, Figure S1. (**B**) ROS generation was assessed by using the DCF-DA assay and measured by flow cytometry, data are expressed as MFI, median intensity of DCF fluorescence. (**C**) Plasma membrane potential was measured as DiBAC4(3) median fluorescence intensity (MFI) in cells.

To assess whether the differences in the cytotoxicity of metallacarborane-modified adenine nucleoside are related to disturbances in cellular homeostasis, we analyzed the specific induction of various types of cell death. Using a viability assay, we estimated the average IC50 for C2′ and C8 derivatives to be 40 ± 11 μM, and this concentration was chosen for subsequent experiments. Programmed cell death phases, early apoptosis, apoptosis/necrosis, and necrosis were determined in the A2780cis cells in a cross-sectional study of all metallacarborane-modified adenosine derivatives **14**–**16**, **18**–**20**, and **22**–**24** and unmodified metalla bis(dicarbollides) (compounds **1**–**3**). The reference compounds, **Ade**, **dAde**, and **25**, were excluded from these analyses as nontoxic, therefore, the mechanisms of this cytotoxicity, understood as processes of apoptosis and necrosis, were not studied for the reference compounds. The cells were treated with the compounds (40 μM), cisplatin (10 μM), or in combination for 48 h. In the combined treatment, the cells were incubated first with one of the metallacarborane-modified adenosine derivatives (for 3 h), followed by a 48 h culture with cisplatin, and the percentage of apoptotic and/or necrotic cells was evaluated using the PI/annexin V assay (Figure 5A). Changes in the plasma membrane potential and ROS production levels were determined in the same cell samples (Figure 5B,C).

Adenosine derivatives or unmodified metalla bis(dicarbollides) were weak inducers of programmed cell death with the exception of compound **23**. When compound **23** was used alone, it induced significant necrosis, and early and late apoptosis in A2780cis cells (in total 26 ± 2.2%; *p* < 0.01, Figure 5A left). A less pronounced proapoptotic effect was observed for other derivatives, **15**, **16**, **18**, **19**, **22**, and **24** (*p* < 0.05) and compound **2** (*p* < 0.01, Figure 5A, left). The detailed results for necrosis and late apoptosis are presented in Figure S1. Compound **23** exerted similar effects on the sensitive cell line, A2780, and resistant cells, A2780cis and A2780cisR (Figure S2, Supplementary Materials). Among the derivatives, compound **23** also induced the greatest increase in ROS formation (*p* < 0.01, Figure 5B, left); the effect of compound **15** was less pronounced, but significant (*p* < 0.01). Moreover, compounds **23** and **22** induced a decrease in plasma membrane potential (PMP, *p* < 0.01, Figure 5C, left), which together with the incorporation of PI into the cells, indicated a dysfunction or even damage to the plasma membrane. Compounds **15** and **19**, which were Fe-containing derivatives, similar to compound **23**, decreased PMP significantly but to a lesser extent (*p* < 0.05, Figure 5C, left).

The effect of the compounds in combination with cisplatin: Cisplatin alone (control, 10 μM) induced apoptosis and necrosis in A2780cis cells, in total 45 ± 2.0% (Figure 5A right); and this was accompanied by ROS outburst (*p* < 0.0001, Figure 5B right) and significant decrease in PMP (increase of MFI, *p* < 0.0001, Figure 5C right). The concentration-dependent decrease in PMP and initiation of apoptosis and necrosis in cisplatin-treated A2780, A2780cis, and A2780cisR cells are shown for comparison in Figure S3A (Supplementary Materials). The increase in apoptosis was accompanied with a proportional increase in the compound accumulation (Figure S4, Supplementary Materials). In combination treatment, it was found that compound **23** considerably increased the proapoptotic effect of cisplatin on cisplatin-resistant A2780cis cells by doubling a percentage of necrotic and apoptotic (PI+/annexin V+) cells (Figure 5A right). The other two Fe-containing derivatives (**15** and **19**) and unmodified ferra bis(dicarbollide) (**2**) also increased necrosis and late apoptosis in cisplatin-treated cells, but by half as much as compound **23** (Figure 5A, right), which showed that the conjugation of ferra bis(dicarbollide) with adenosine has an advantage over unmodified metallacarborane. ROS production induced by cisplatin also increased significantly in cells incubated with the Fe-containing compounds **23**, **15**, and **2** (Figure 5B, right). Cisplatin was an efficient inductor of ROS production, which accompanied apoptosis. Cells incubated with compound **23** and cisplatin showed significantly greater ROS production than cells incubated with cisplatin only. Moreover, compound **23** (*p* < 0.05), which is similar to other Fe-containing derivatives (*p* < 0.05) and C2′ derivatives containing Co and Cr ions (*p* < 0.05 and *p* < 0.001, respectively), augmented the cisplatin effect on PMP (Figure 5C, right). Exceptionally, compounds **16**, **2**, and **1** acted here as inhibitors of cisplatin-induced PMP depolarization as in the case of apoptosis (Figure 5A,C, right). In combination treatment, it was found that the Fe-containing compounds exerted an opposite effect on apoptosis in comparison to the effects of compounds **16**, **2**, or **1**.

### 2.6. Phases of Cell Cycle

**Figure 6:**
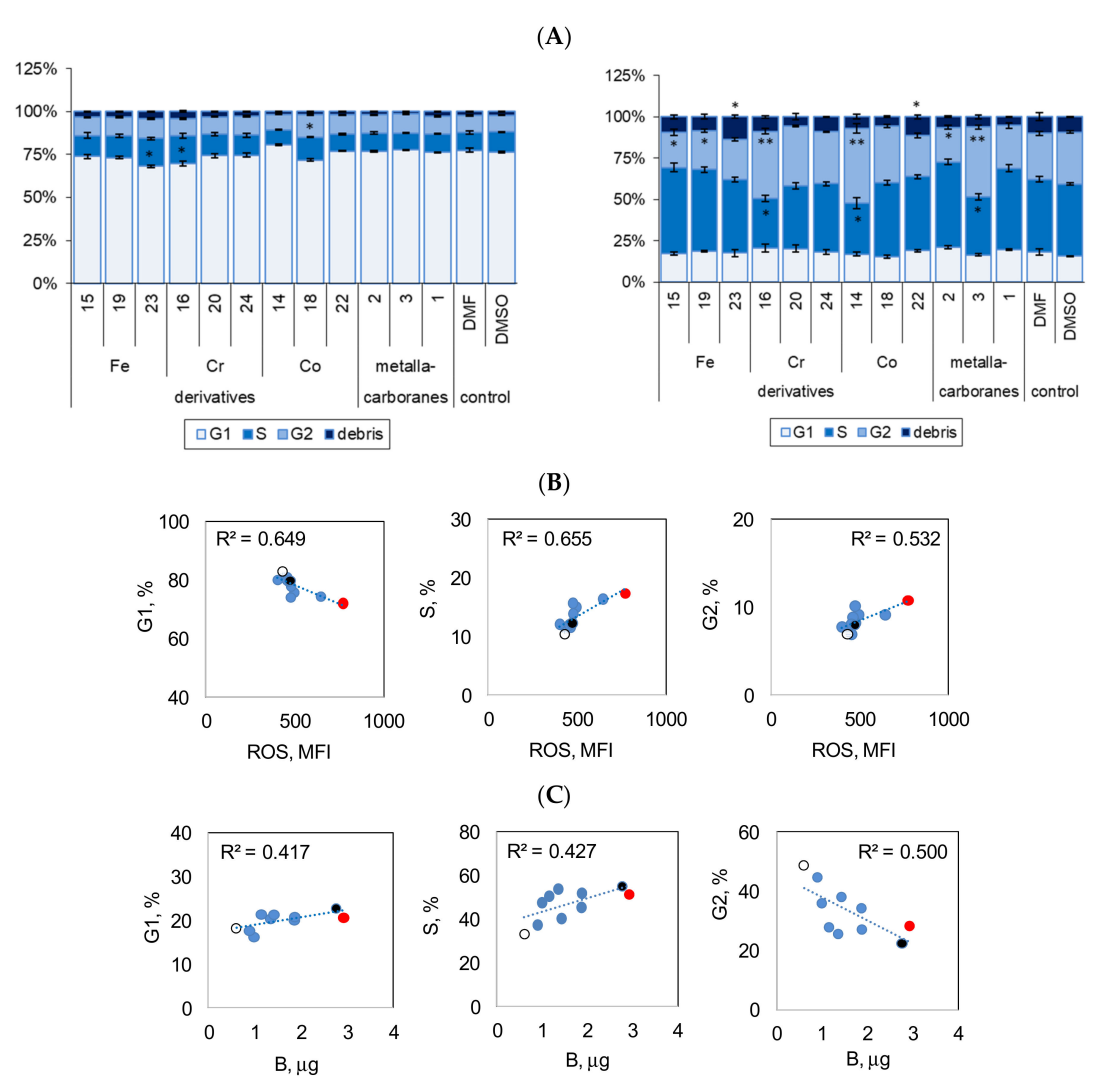
Effect of compounds **1**–**24** alone (40 μM) and in combination with cisplatin (10 μM) on the cell cycle. (**A**) Cumulative percentage of cells at different phases of cell cycle is shown: G1—one set of DNA; S—commence DNA replication; G2—pre-mitotic phase with double the amount of DNA; debris—DNA fragmentation (apoptosis); cell cycle phases were determined by intracellular DNA staining using PI/RNAse method; data are the mean of three to six independent experiments ± SEM; * *p* < 0.05, ** *p* < 0.01, compound *versus* without compound; Student t-test with Bonferroni correction. N,N-dimethylformamide (DMF) or dimethyl sulfoxide (DMSO) (< 0.04%, not toxic) was used as the control. (**B**) Relationship of cell cycle phases to ROS production. Significant relationships: G1 phase, *p* = 0.002; S phase, *p* = 0.001; G2 phase, *p* = 0.007. Data for cells incubated with the compounds. R^2^, Pearson correlation coefficient values. (**C**) Boron content in A2780cis cells in relation to cell cycle phases (G1, S, and G2); red dot—compound **23**, black dot—compound **2**, empty circle—compound **14**. Significant relationships: G1 phase, *p* = 0.045; S phase, *p* = 0.044; G2 phase, *p* = 0.025. R^2^, Pearson correlation coefficient values. Data for cells incubated with the compounds followed by treatment with cisplatin.

Keeping in mind that induction of programmed cell death is accompanied with disturbances in cell division, the effect of the compounds on cell cycle phases was also determined in the same cell samples (Figure 6A). Compounds **23**, **16**, and **18** showed slight but significant inhibition of the cell cycle at the S and G2 phases as well as DNA fragmentation (**23** and **16**, Figure 6A, left chart). In combination treatment, compounds **23** and **22** significantly increased the effect of cisplatin on DNA fragmentation (debris, Figure 6A, right chart). Compounds **15**, **19**, and **2** significantly reduced the number of cells in the G2 phase (*p* < 0.05, Figure 6A). Compounds **16**, **14**, and **3** induced an S/G2 shift in cells, reduced the number of cells in the S phase, and increased the number of cells in the G2 phase (*p* < 0.01, Figure 6A). The changes in cell cycle phases were closely related to ROS production and the cellular accumulation of the compounds (Figure 6B,C).

### 2.7. Effect of Compound 23 on Tumor Suppressor Protein p53 Expression and its Phosphorylated and Acetylated Forms

P53 is involved in the cellular response of ovarian cancer cells to chemical factors (xenobiotics, anticancer drugs) or physical stresses (e.g., UV), being a key regulator of apoptosis. The phosphorylation of the p53 protein regulates the expression of genes that mediate cell cycle arrest and/or apoptosis [41]; the acetylation prevents p53 from degradation [42]. Expression, phosphorylation, and acetylation of the p53 protein were determined by the intracellular staining method using fluorescently labeled monoclonal antibodies specific for phosphoserine-15 p53 (pSer15-p53), phosphoserine-37 p53 (pSer37-p53), and p53 acetylated on lysine382 (ac-p53) (see the Section 4). A2780 cells express wild type (wt) p53, whereas OVCAR-3 and SKOV-3 cells contain different p53 mutants. Therefore, specific antibodies (clone DO-7) recognizing both wt-p53 and the mutant p53 were used for intracellular detection of the protein. 

**Figure 7:**
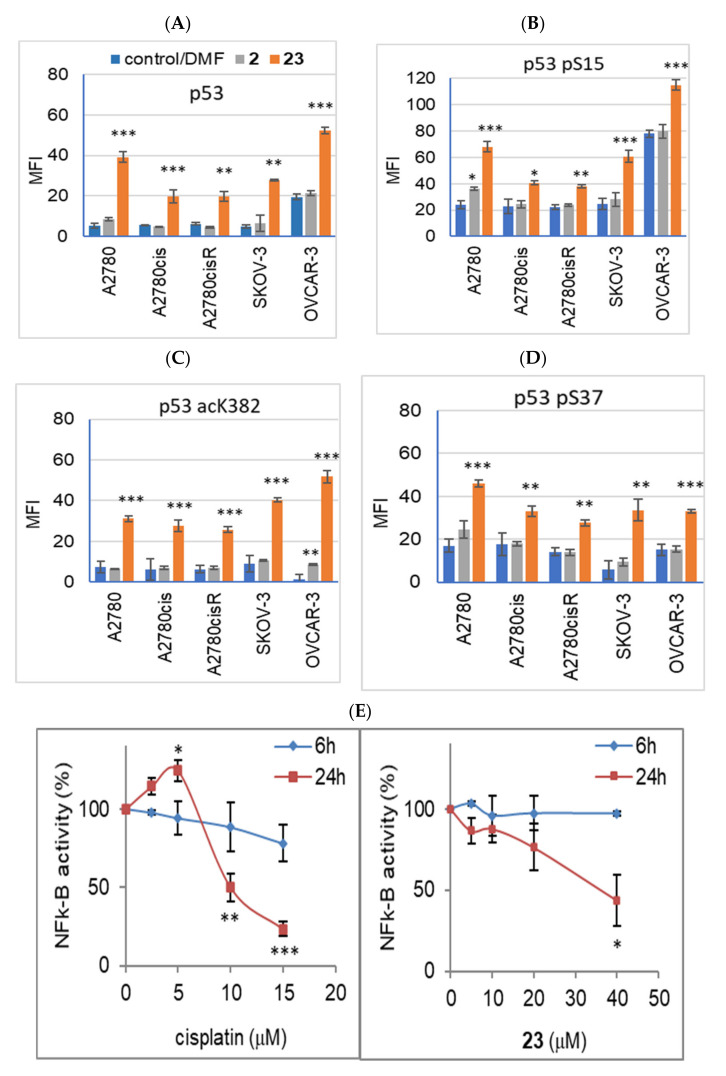

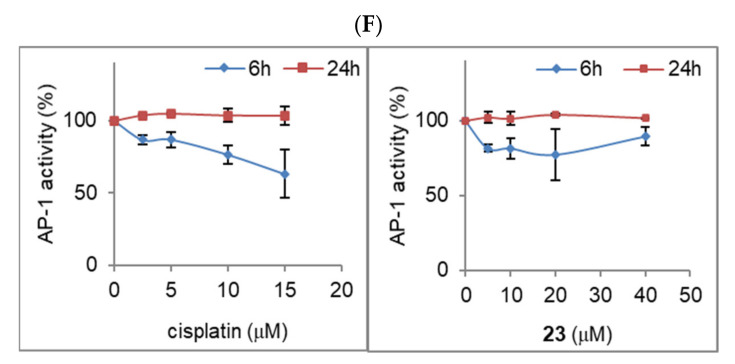
(**A**–**D**) Effect of 2′ derivative of adenosine modified with ferra bis(dicarbollide) (compound **23**) and the corresponding parent sandwich ion (compound **2**) on p53 expression levels and its phosphorylation and acetylation in ovarian cancer cell lines. The expression of p53 was measured using the intracellular labeling method with specific antibodies for human wild type and mutant p53, clone DO-7 (p53, (**A**)); p53 phosphorylated at Ser15 (p53 pS15, (**B**)); p53 phosphorylated at Ser37 (p53 pS37, (**C**)); and p53 acetylated in the C-terminal region at Lys382 (p53 acK382, (**D**)). Intracellular labeling was performed as described in the Materials and Methods (Section 4). Data are expressed in relative fluorescence units (MFI, median fluorescence intensity) as means ± SEM values calculated for three–four independent experiments. Cells were incubated for 24 h with compounds **2** or **23** (40 μM). DMF was used as the control (0.01%, 0.02%, or 0.04%, not toxic, not effective on p53 protein expression level). (**E**,**F**) Effect of compound **23** (5–40 μM) and cisplatin (2.5–15 μM) on the NFk-B (**E**) and AP-1 (**F**) activity determined in stable transfected cell lines (SKOV-3 and HeLa, respectively). Cells were incubated with the compound for 6 h and 24 h. Luminescence was measured in RLU (relative luminescence units), and expressed as the percentage of control values for untreated cells. The mean ± SEM values were calculated for three independent experiments. * *p* < 0.05, ** *p* < 0.01, and *** *p* < 0.001, compounds *versus* control (Student’s *t*-test with Bonferroni correction for multiple comparisons).

Interestingly, we observed substantial differences in the basal p53 protein expression level. A2780, A2780cis, A2780cisR, and SKOV-3 cell lines showed low expression levels of p53 (Figure 7A) and its phosphorylated or acetylated forms (pSer15-p53, pSer37-p53, or ac-p53, Figure 7B–D; and Figure S5A, Supplementary Materials). In contrast, the OVCAR-3 cells exhibited considerably high expression levels of p53 (Figure 7A) and pSer15-p53 (Figure 7B), while expression of ac-p53 and pSer37-p53 was at a marginal level of detection (Figure 7C,D, respectively; and Figure S5A, Supplementary Materials). However, all cell lines showed significant and considerable increases in basal p53 expression and its acetylation in response to cisplatin, which is a strong inducer of p53 activation (Figure S5B, Supplementary Materials). Increased expression levels of pSer15-p53 and pSer37-p53 were noted in all cell lines except for OVCAR-3 cells in response to cisplatin (Figure S5B, Supplementary Materials). There were no changes in pSer15-p53 expression (initially high in the control, untreated cells), but only a slight significant increase in pSer37-p53 expression levels in OVCAR-3 cells (Figure S5B, Supplementary Materials).

**Figure 8:**
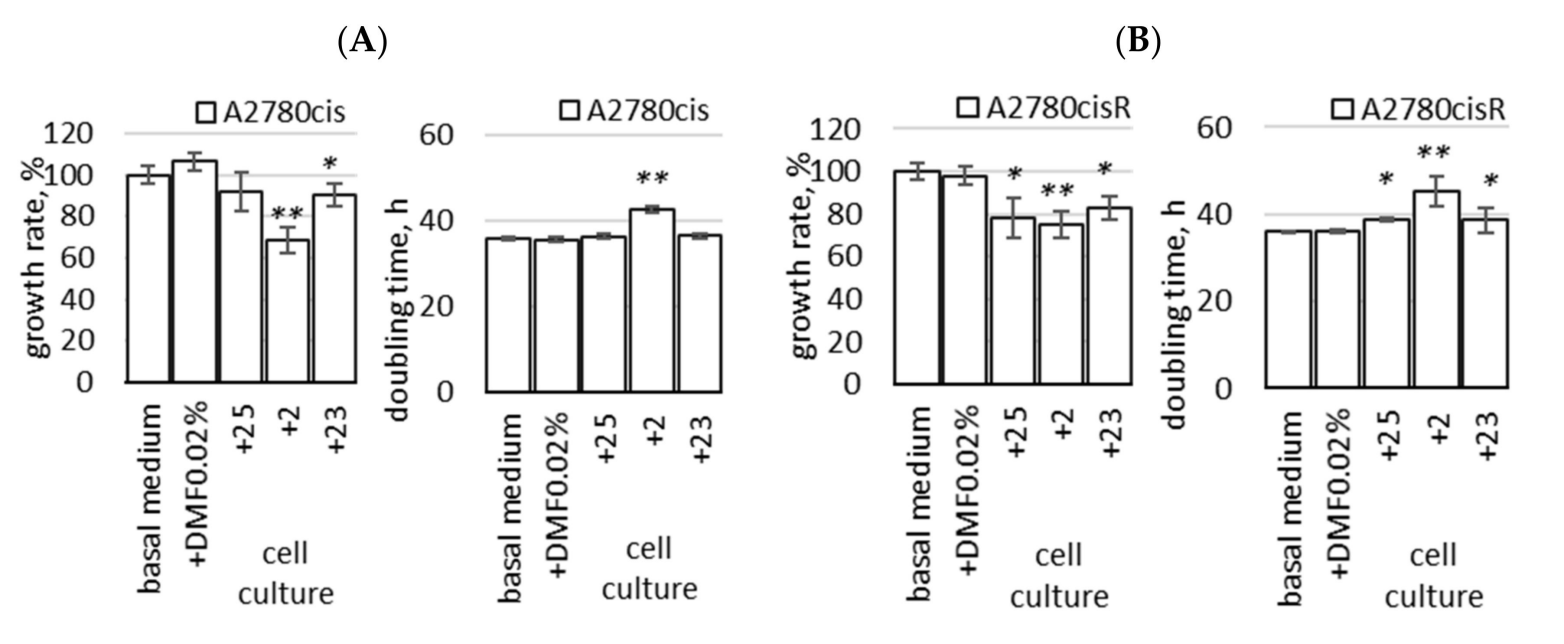
Growth rate and doubling time of A2780cis (**A**) and A2780cisR (**B**) cells cultured long-term in a medium with the addition of compound **23** (2′ adenosine derivative modified with ferra bis(dicarbollide) ion); compounds **25** (analogous 2′ derivative containing *nido*-7,8-dicarbaundecaborate) and **2** (unmodified ferra bis(dicarbollide)) were used for comparison. The cells were grown for two weeks in the basal medium with the addition of compounds **25**, **2**, and **23** (cultures named “+23_cells”, “+25_cells” and “+2_cels”) or without compounds (“basal medium” and “DMF 0.02%” control), as described in the Section 4. Cell growth rate is expressed as percentage of the number of control cells harvested at the end of the culture period (100%, equal to 7.5–10 × 10^6^ cells). The mean values ± SEM of three experiments are shown. * *p* < 0.05, ** *p* < 0.01; Student *t*-test with Bonferroni correction was calculated for compounds *versus* control cells.

Fe-containing compound **23** (C2′ derivative), with the highest proapoptotic activity, was selected for studies on the effect of the derivatives on the expression and post-translational modification of p53. Among all the cell lines, compound **23** (40 μM, 24 h) induced an apparent increase in the expression of p53 and its phosphorylated or acetylated forms (pSer15-p53, pSer37-p53, and ac-p53, Figure 7A–D). The largest increase in p53 and pSer15-p53 expression levels was found in cisplatin-sensitive cells, A2780 and OVCAR-3 (Figure 8A,B). Compound **23**, unlike cisplatin, enhanced p53 phosphorylation at Ser15 (by 30%) and induced phosphorylation at Ser37 in OVCAR-3 cells (Figure 8B). Cisplatin-resistant cell lines A2780cis and A2780cisR showed a significantly lower increase in p53 expression than the parental cell line, A2780 (*p* < 0.01, and *p* < 0.001, calculated for percentage values). In the SKOV-3 cells, p53 expression, enhanced by compound **23**, was visibly lower than that in the cisplatin-sensitive cell lines, that is, A2780 and OVCAR-3 (Figure 8A). Compound **2**, unlike compound **23**, did not increase the expression of p53 or its phosphorylation or acetylation (Figure 8A,B). Only a slight but significant increase in pSer15-p53 expression was observed under the influence of compound **2** in A2780 cells (*p* < 0.05, Figure 8). Compound **2** also induced slight ac-p53 expression in the OVCAR-3 cells. The baseline expression of ac-p53 in the OVCAR-3 cells was at a marginal level of detection (Figure 7C); therefore, the effect of compound **2** was significant (*p* < 0.01, Figure 7C). A comparison of the results for compounds **23** and **2** is shown in Figure S6 (Supplementary Materials).

The effect of compound **23** was dose-dependent, that is, increasing doses of compound **23** (10–40 μM, 24 h) induced an apparent increase in p53 expression in A2780, SKOV-3, and OVCAR-3 cells, whereas compound **2** was not effective (Figure S6). The expression level of phosphorylated p53 (Ser15) also increased in a dose-dependent manner in A2780 and SKOV-3 cell lines under the influence of increasing doses of compound **23** (Figure S6). The results for compound **23** (effective) and compound **2** (ineffective), expressed as fold change (treated/control), are summarized in Table S1 and Figure S7 (Supplementary Materials).

### 2.8. Activation of Transcription Factors—AP-1 and NF-κB

Elevated ROS levels in cancer cells activate redox sensitive transcription factors including nuclear factor kappa B (Nf-κB) and activator protein-1 (AP-1) [43]. Unfortunately, AP-1 and Nf-κB are upregulated in tumors and directly correlate with the cancer cell proliferation rate and their chemoresistance [44].

Intrigued by the differential effects of metallacarborane-modified adenine nucleosides on the induction of apoptosis and necrosis, we aimed to assess the changes in the functionality of the key transcriptional factors involved in cellular homeostasis. The activity of AP-1 and Nf-κB transcription factors was determined in our study using stably transfected cells, HeLa luciferase AP-1 reporter cell line, and SKOV-3 luciferase Nf-κB reporter cell line, as described in the Materials and Methods (Section 4). Cells grown in a monolayer were incubated for 6 h and 24 h with compound **23** or cisplatin at concentrations ranging from 5–40 μM for compound **23** and 2.5–15 μM for cisplatin. The concentration ranges were adjusted according to the toxicity of the compounds, determined using the NR viability assay for both HeLa AP-1 and SKOV-3 Nf-κB cell lines, and calculated respectively as 22 ± 0.6 and 18 ± 3.0 μM for cisplatin and 54 ± 1.9 and 41 ± 2.6 μM for compound **23**. Luminescence measurements were then performed to determine the AP-1 and Nf-κB promoter expression levels (Figure 7E,F).

Cisplatin concentrations of 10 μM and 15 μM significantly reduced the activation of Nf-κB after 24 h (80% decrease, Figure 7E) and transiently (after 6 h) downregulated the AP-1-mediated promoter activity at a concentration of 15 μM (Figure 7F). A slight increase in Nf-κB activity was observed at a cisplatin concentration of 5 μM (Figure 7E). Compound **23** significantly decreased Nf-κB activity after 24 h of incubation with the cells at the highest concentration of 40 μM (60% decrease, Figure 7E). Compound **23** did not affect the AP-1 expression levels (Figure 7F).

### 2.9. Long-Term Cell Cultures

The refractory or recurrent ovarian cancer second-line chemotherapy protocols are based on multi-drug regimens. Usually, platinum preparations are used in combination with paclitaxel (PTX), doxorubicin (DOX), or gemcitabine (GEM) for the treatment. However, prolonged exposure to a single chemotherapeutic agent may also lead to the development of resistance to other, structurally unrelated drugs. A similar phenomenon is observed in long-term cancer cell cultures. In this experimental protocol, we examined whether chronic exposure to sub-toxic doses of the compound promoted possible cross-resistance to cisplatin, carboplatin, DOX, PTX, or GEM. A2780cis and A2780cisR cells were cultured for 16 days in a culture medium supplemented with compound **23** [2′-adenosine derivative modified with ferra bis(dicarbollide)] (20 μM) or reference compounds **25** (2ʹ derivative of adenosine modified with *nido*-7,8-dicarbaundecaborate, without metal ion) and **2** [unmodified ferra bis(dicarbollide)]. The [4+3+1]_2_ regimen, that is, four days with and three days without the compound, plus one day of passage, repeated twice was used; the protocol is described in detail in the Section 4 (4.10. Long-Term Cultures). The effect of prolonged cultivation of cells in a medium with the addition of the compounds was evaluated after removing the medium enriched with the compounds, replacing it with the basal medium, and processing the culture for an additional three days. The control cells were cultured in a basal medium and a medium supplemented with a vehicle (DMF, 0.02%, not toxic). 

#### Growth Rate and Doubling Time

First, the total cell number was determined at the end of the culture period, and the cell growth rate and doubling time were calculated (Figure 8). There was no significant difference in the growth rate of the control cells cultivated in the basal medium and the medium supplemented with a vehicle (DMF, 0.02%, Figure 8A,B). The addition of compound **23** to the cell culture decreased the growth rate in both cell lines, A2780cis and A2780cisR, slightly but noticeably (*p* < 0.05, Figure 8A,B). Reduced cell growth rate was observed in the culture medium containing compound **2**; the growth rate decreased by almost 40% in A2780cis cells (Figure 8A). A significant decrease in growth rate was also found in the A2780cisR cells cultured with the reference compounds **25** (*p* < 0.05, Figure 8B) and **2** (*p* < 0.01, Figure 8A,B). The effect of cell culture with the compounds in A2780cisR cells was the most pronounced.2.9.2. Susceptibility to Cisplatin, Carboplatin, DOX, PTX, and GEM in Long-Term Cultures

**Figure 9:**
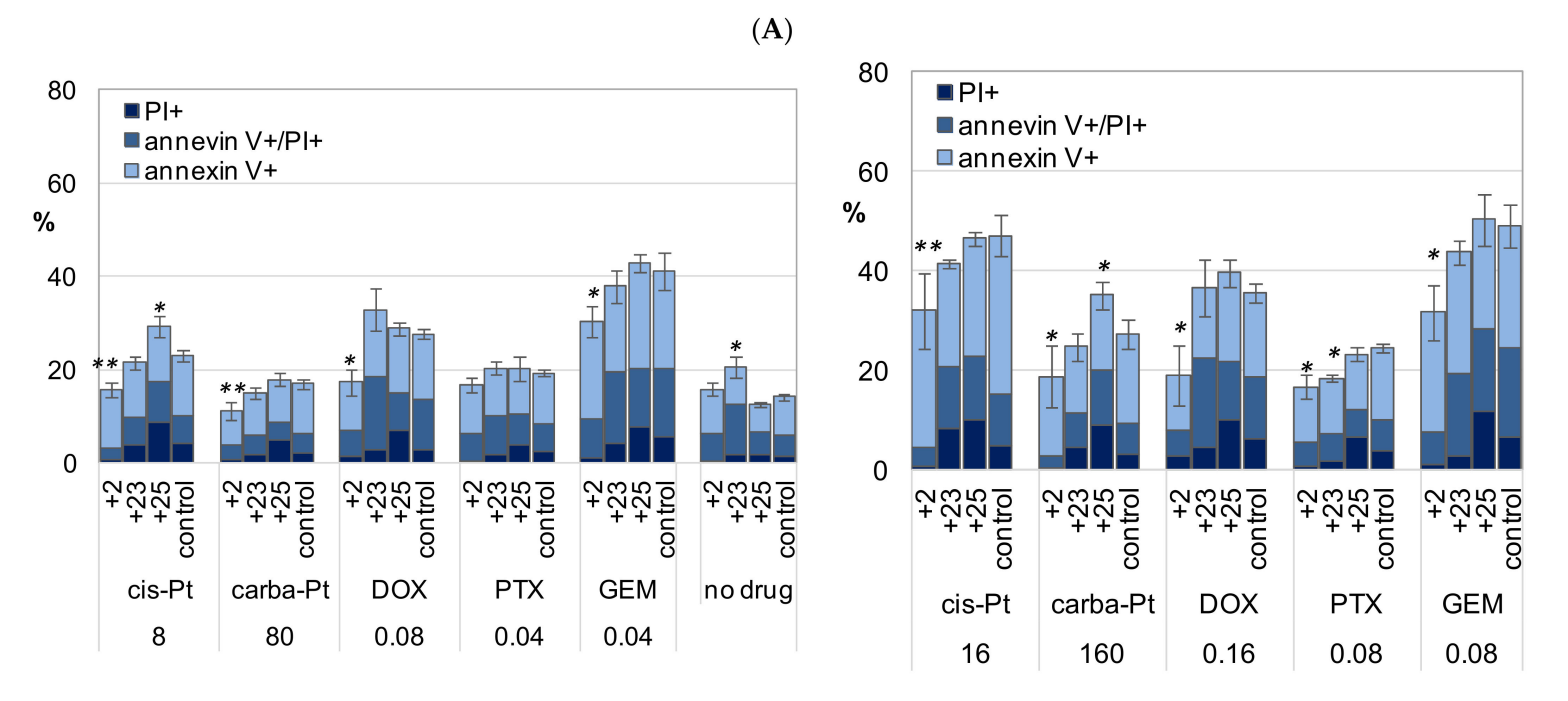

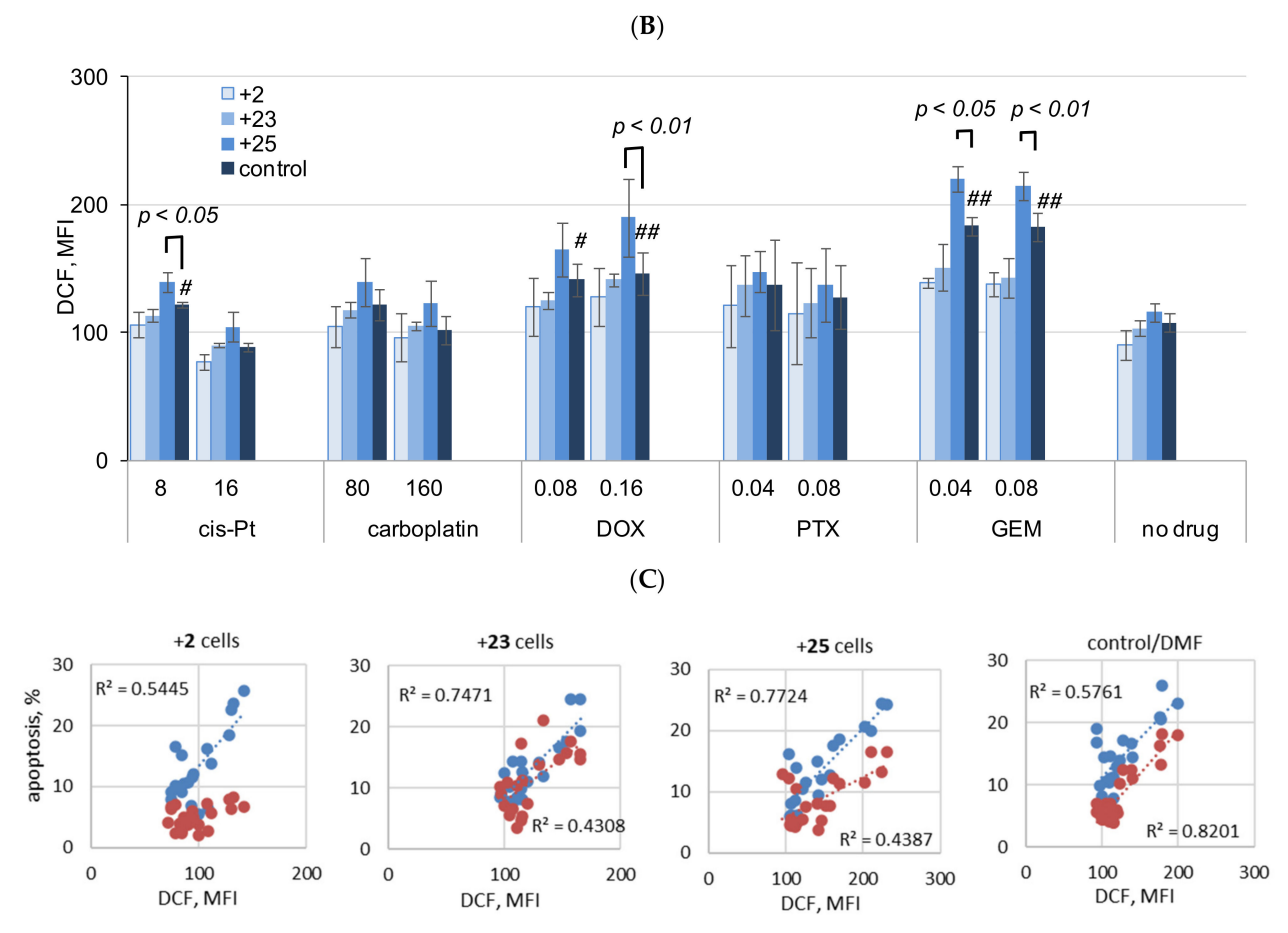
Apoptosis and necrosis (**A**), and ROS production (**B**) in A2780cisR cells cultured long-term in medium with the addition of compounds **23**, **2**, or **25**, and then exposed to low and high concentrations of drugs, cisplatin, carboplatin, DOX, PTX, or GEM (at IC50 and ½ × IC50). Relationship of apoptosis to ROS production in cells cultured with the compounds, and the control cells, cultured without the compounds is shown in (**C**). Cell death was determined in terms of necrosis and apoptosis of cells stained with PI and annexin V-FITC. Double-stained cells were analyzed by flow cytometry as described in the Materials and Methods (Section 4). Cumulative percentage of necrotic/nonapoptotic (PI+/annexin V−), late apoptosis (annexin V+/PI+), and early apoptosis (annexin V+/PI−) cells is shown; (**A**) left—lower doses, (**A**) right—higher doses of drugs; “control”—cells cultured without compounds, with vehicle (DMF < 0.08%, not toxic). The mean ± SEM of three experiments are shown. *p* values (* *p* < 0.05 and ** *p* < 0.01) were calculated for nonapoptotic cells cultured with compound *versus* without compound; Student t-test with Bonferroni correction. Detailed statistical analysis is presented in the Supplementary Materials. Intracellular ROS production was measured by flow cytometry in DCF-DA stained cells (MFI, median intensity of fluorescence). The mean ± SEM of three experiments are shown in (**B**). *p* values were calculated by the Student t-test, cells cultured with compound *versus* without compound; ^#^ *p* < 0.05, ^##^ *p* < 0.01 drug *versus* no drug. Symbols “+25”, “+2”, and “+25” stand cells cultured with addition of the respective compound, **25**, **2**, or **25** to culture medium. (**C**) Blue dots—early apoptosis (annexin V-positive/PI-negative cells), red dots—late apoptosis (annexin V and PI positive cells); data for A2780cisR cells treated with cisplatin, carboplatin, DOX, PTX, GEM, and cells without drugs were collected and analyzed; R^2^, Pearson correlation coefficient values. Cisplatin (8 μM), DOX (0.08 μM and 0.16 μM), or GEM (0.04 μM and 0.08 μM) induced cellular ROS production in the cells not treated with the compounds (control/DMF, Figure 9B). In all cases, ROS production increased in the cells exposed to the drugs, cultured previously with compound **25**. ROS production in the cells exposed to the drugs, grown previously in the presence of compounds **2** or **23**, was at the level of the cells cultured in basal medium with the DMF vehicle only (Figure 9B).

Next, susceptibility to cisplatin, carboplatin, DOX, PTX, and GEM was evaluated in A2780cisR cells collected from different culture conditions, that is, cultured with or without the compounds. At the end of the culture in the presence of the compounds, the cells were treated with cisplatin, carboplatin, DOX, PTX, or GEM, as described in the Section 4. The culture of A2780cisR cells in the presence of compounds **23** and **25** did not cause cross-resistance to the drugs. The culture of the cells in the presence of compound **25** resulted in a significant increase in sensitivity to cisplatin (8 μM) and carboplatin (160 μM) (Figure 9A,B, respectively).

The percentage of necrotic cells (PI+) and cells in late apoptosis (PI+/annexin V+) increased twofold in “+**25** cells” compared to that of the cisplatin and carboplatin-treated control, while the percentage of early apoptosis cells remained unchanged (Figure 9A,B). An increase in late apoptosis cells was also found in the control “+**23** cells” not treated with cisplatin, carboplatin, DOX, PTX, and GEM (“no drug”, Figure 9A). Detailed analysis for apoptotic and necrotic cells is presented in the Supplementary Materials (Figure S8A,B, respectively, Supplementary Materials).

In contrast to supplementation with compounds **25** or **23**, culture of the cells in the presence of compound **2** induced drug resistance (“+**2** cells”, Figure 9A,B), which indicates that the cells overcame the relatively proapoptotic action of compound **2** and triggered some adaptive mechanisms. Although compound **2** was relatively proapoptotic in the short-term treatment of cells (48 h), the percentage of necrotic and apoptotic cells diminished considerably in the long-term treated “+**2** cells” in comparison to that of the cells cultured without compound **2** exposed to cisplatin, carboplatin, DOX, PTX, or GEM (Figure 9A,B; and Figure S8A,B, Supplementary Materials). The cumulative percentage of cells in different phases of apoptosis and necrosis revealed a significant increase in resistance to all drugs for “+**2** cells” (Figure 9A,B). The percentage of necrotic cells (PI+/annexin V−) and cells in the late phase of apoptosis (PI+/annexin V+) decreased in all drug treatments for “+**2** cells” (detailed analysis is shown in Figure S8, Supplementary Materials). Increased drug resistance, that is, greater viability of “+**2** cells” than the control cells was confirmed in the NR assays (Figure S9, Supplementary Materials), which confirms that the adenosine conjugate is a superior anticancer compound to the parent metalla bis(dicarbollide). 

Changes in ROS production including in necrotic cells were determined in the entire cell population. Necrotic cells (PI+/annexin V−) are generally dead, that is, devoid of vital functions, which may interfere with the assessment of these parameters for living cells (underestimate the results). The analysis of the normalized results confirmed the results for crude data, and the differences between the drug-treated and untreated cells were more pronounced for PTX, and a significant increase in ROS levels for cells cultured with compound **25** and exposed to DOX (0.08 μM) was revealed (Figure S10, Supplementary Materials).

Drug-induced increase in ROS production was accompanied with a proportional increase in early or late apoptosis (early necrosis) in cells previously cultured with compounds **23** or **25** or without any compound (Figure 9C). In contrast, the typical relationship between ROS production and cell apoptosis was maintained only for the early apoptotic stages of cells cultured with compound **2** (“+**2** cells”). A parallel increase in late apoptosis cells was disturbed in “+**2** cells”, indicating inhibition of this process in cells cultured with long-term treatment of compound **2** (Figure 9A).

In summary, the results indicated the inhibition of the growth of A2780cisR cells cultured with compound **2**, with a simultaneous increase in drug resistance. In contrast, prolonged cell culture with the addition of compounds **25** and **23** reduced the rate of cell division, but also increased susceptibility to drug cytotoxicity (or left drug susceptibility unchanged).

### 2.10. Three-Dimensional Spheroid Cell Cultures

**Figure 10:**
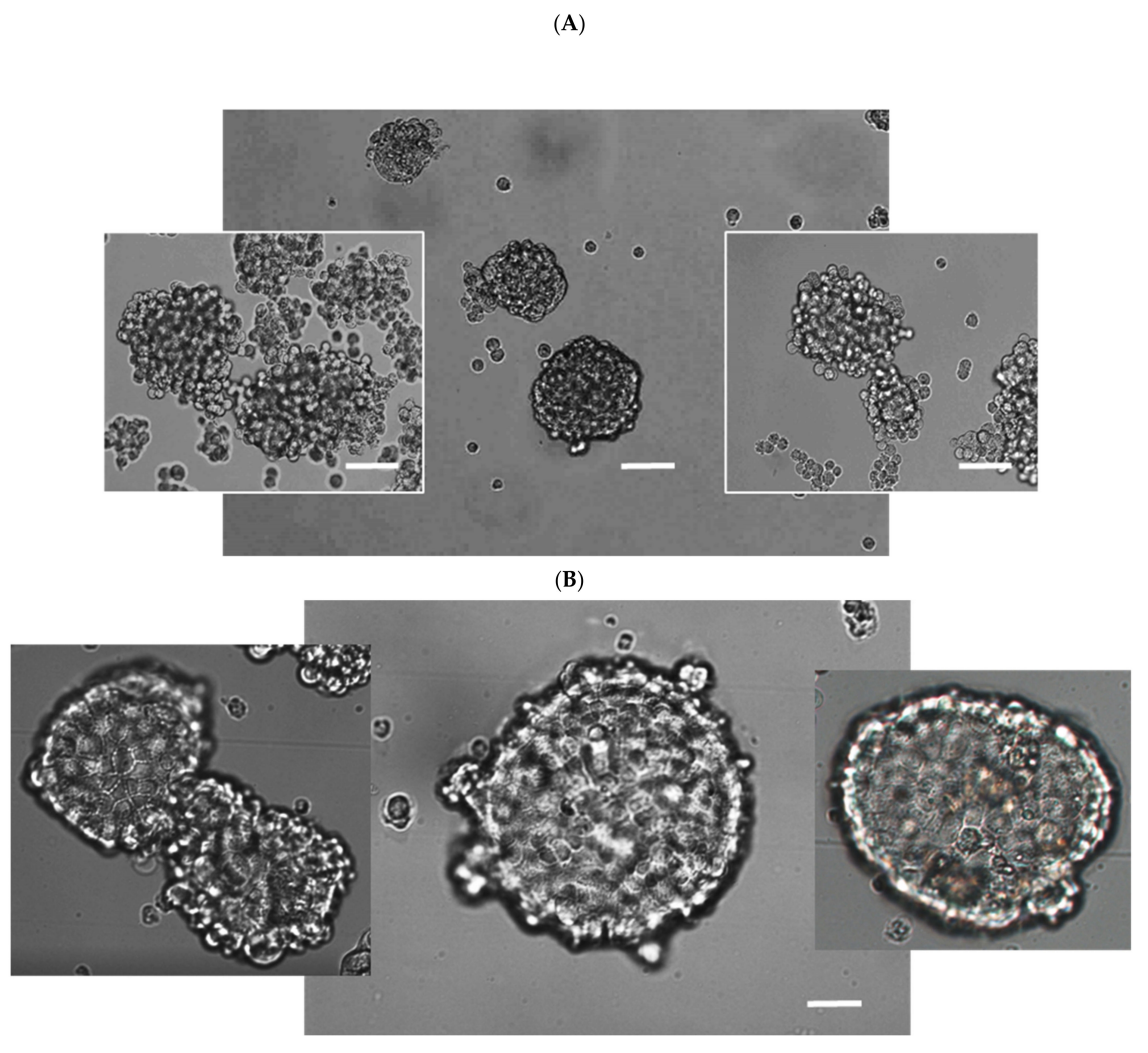
Differential interference contrast (DIC) image of the spheroids formed by A2780 cells (**A**) and OVCAR-3 (**B**) cells. Cells were cultured in ultra-low-attaching dishes as described in the Section 4. Scale bar 50 μm. Forming spheroids with a fairly loose cell packing (second day of culture) are shown as the side images in Figure (A). Mature A2780 spheroids are presented in the center. In general, the A2780 cells were smaller in size than the OVCAR-3 cells.

Spheroid aggregates of cancer cells show increased survival capacity and resistance to chemotherapy. Three-dimensional spheroid cell cultures were applied in this study as a particular model for in vitro evaluation of cancer cell chemoresistance [45,46]. Three-dimensional spherical cell cultures were derived in our study from ovarian cancer cell lines with different sensitivities/resistances to cisplatin, A2780, A2780cis, A2780cisR, SKOV-3, and OVCAR-3. Effect of compound **23** alone or in combination with cisplatin on spheroid viability was determined. The cells of each cell line showed different rates of spheroid formation: A2780 and its resistant sublines—7 days, SKOV—3–2 weeks, and OVCAR-3—3 weeks. A2780 spheroids could last in the culture for up to three weeks, whereas A2780cis spheroids disintegrated after two weeks. Therefore, spheroids derived from the A2780 line and its sublines (A2780cis and A2780cisR) were used for the experiments on the seventh day of culture.The spheroids of each cell line had a different shape and size distribution, which is a characteristic feature of a given cell line (Figure 10A,B). Cells of the A2780 line formed irregular medium-sized spheroids (50–100 μm). The packing of A2780 cells was rather loose, although some of them were quite regular (Figure 10A). The structure of the A2780cis spheroids was loose, resembling an aggregate conformation. In contrast, spheroids formed by OVCAR-3 cells were large (200–300 μm) with compact structures and tightly adjacent cells (Figure 10B). Nomarski’s DIC microscopy with three-step focusing was used to image large spheroids. The method is described in detail in Figure S11 (Supplementary Materials). The spheroid surface was also examined using time-lapse imaging along the z-axis (see the Section 4).

**Figure 11:**
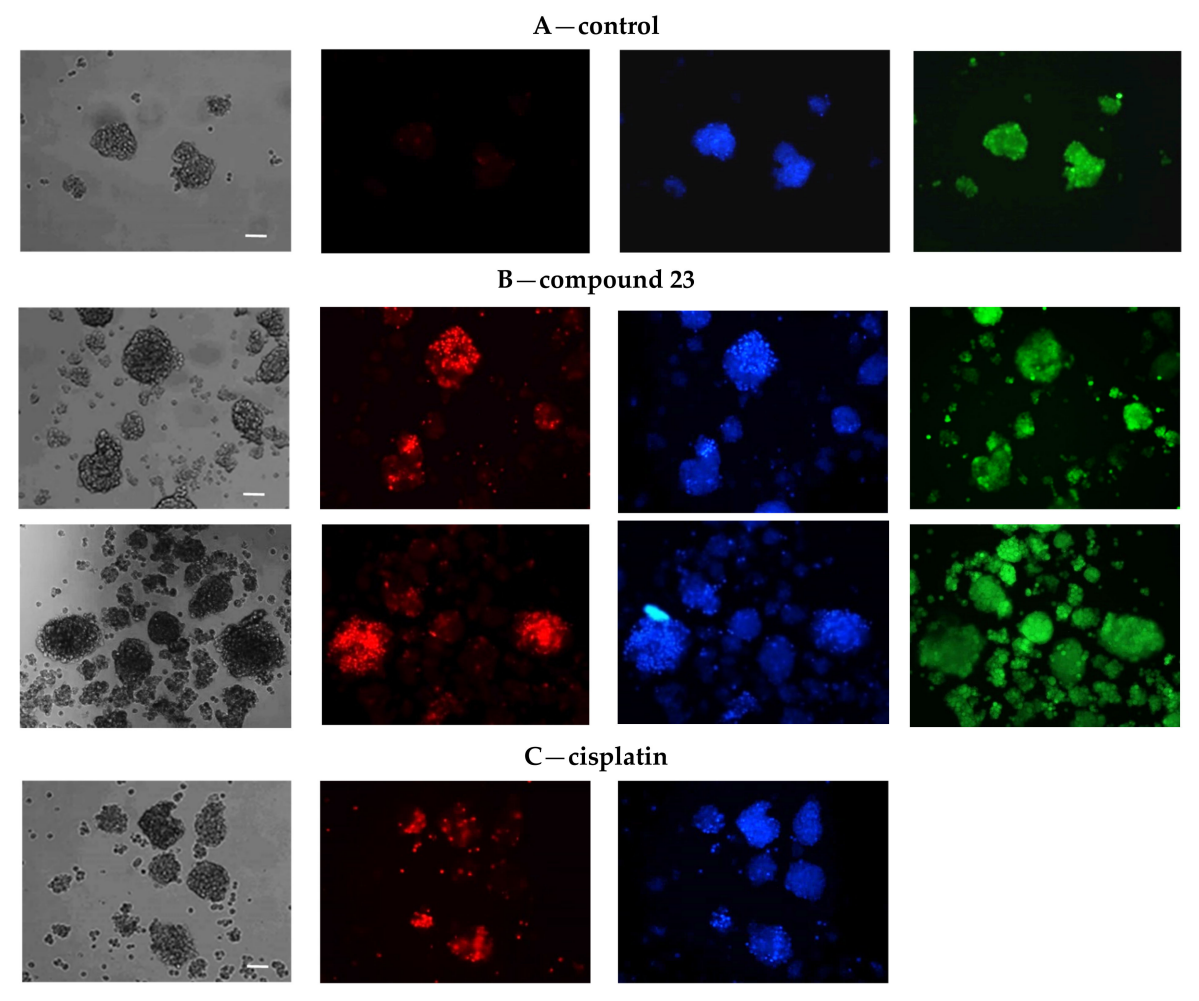
Microphotographs of A2780 spheroids (**A**) control, (**B**) treated with compound **23**, and (**C**) cisplatin. The spheroids were cultured for 48 h with compound **23** (40 μM), cisplatin (10 μM), or untreated. Next, the cells were transferred to 8-chamber slides and then stained with PI (red, dead cells), Hoechst 33258 (blue fluorescence, nuclei), and additionally with CellTrackerGreen (green fluorescence, cytoplasm, **A**,**B**), as described in the Section 4. Spheroids were photographed under an epifluorescence microscope Nikon Eclipse TE2000-U equipped with digital color camera (10 × PlanFluor objective, scale bar 50 μm, focus on dead PI-stained cells). Contrast images of A2780 spheroid morphology were captured using the differential interference contrast (DIC, Nomarski) focusing on spheroid boundary (the method of imaging is described in Supplementary Materials, Figure S11). At least 6–10 fields were collected per slide. Three separate experiments were carried out. Representative microphotographs are presented. In all photographs of PI (red) stained cells (**A**–**C**), the image brightness was equally increased by 20% for better visibility of dead cells in the control (**A**).

The viability of the spheroids was examined using contrast and fluorescence microscopy. Spheroids were stained with PI after a certain period of growth and formation. Only a slight, insignificant percentage of dead cells were found in the control, that is, untreated spheroids (images for A2780 spheroids are shown in Figure 11A). Compound **23** (40 μM) caused the death of large A2780 spheroids (Figure 11B). Dead cells were “trapped” in the spheroids, indicating that compound **23** acted inside the structure. In contrast, cisplatin (10 μM) was toxic mainly to peripheral cells, which were visible as loose dead cells “exfoliated” on the surface or coming from disintegrated structures regardless of their size (Figure 11C). Spheroid disintegration under the influence of cisplatin was mainly observed in the A2780 line and its sublines. 

**Figure 12:**
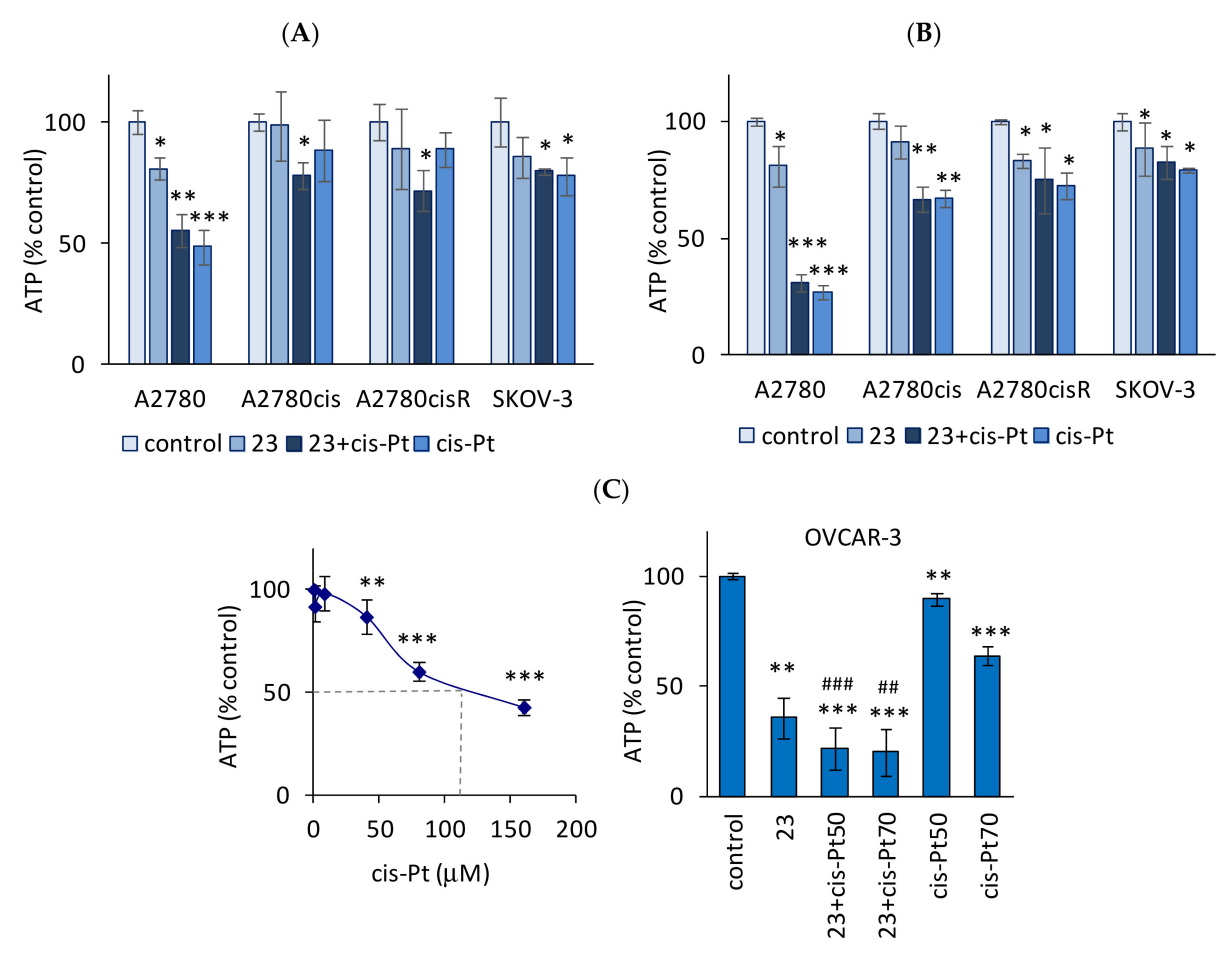
Effect of compound **23** alone and in combination with cisplatin on spheroid viability. Spheroids derived from A2780, A2780cis, A2780cisR cells, and SKOV-3 cells were incubated with compound **23** (40 μM), cisplatin (10 μM), or in combination. In the combined treatment, the cells were incubated first with compound **23** (for 3 h) followed by 48 h (**A**) and 72 h (**B**) of the treatment with cisplatin. OVCAR-3 cells (**C**) were resistant to cisplatin (left); therefore cisplatin concentration for combined treatment was 50 μM and 70 μM (right). DMF was used as the control (<0.06%, not toxic). The effect of different concentrations of DMF on ATP content is presented in the Supplementary Materials (Figure S12). Cell viability was determined by using a CellTiter-Glo 3D Viability Assay (Promega), based on the luminescent measurement of ATP levels (RLU—relative luminescent units). Data are expressed as a percentage of control values for untreated cells (the mean RLU ± SEM values for A2780, A2780cis, A2780cisR, SKOV-3, and OVCAR-3 cells were respectively 156 ± 9, 146 ± 6, 163 ± 18, 171 ± 25, and 175 ± 31). The mean ± SEM values were calculated for 3–4 experiments. * *p* < 0.05, ** *p* < 0.01, and *** *p* < 0.001, compounds *versus* control; ^##^ *p* < 0.01 and ^###^ *p* < 0.001, combined treatment *versus* cisplatin (C); Student’s t-test, differences significant for multiple comparisons are shown (Bonferroni correction).

Intrigued by the morphology changes in spheroids treated with the investigated compounds, we measured the ATP level as a quantitative indicator of the functionality of viable cells (Figure 12A–C). Spheroid viability was tested on the second and third days of treatment with the compounds. Initially, the standard protocol was used for adherent cells, 48 h incubation with compound **23** (40 μM) or cisplatin (10 μM) or in combination (Figure 12A). In the combined treatment, the cells were incubated first with compound **23** (for 3 h), followed by treatment with cisplatin. Compound **23** alone slightly, but significantly, decreased the viability of cisplatin-sensitive A2780 spheroids (Figure 12A); the cytotoxicity of cisplatin was more pronounced (50%) and remained unchanged after combination treatment with compound **23** (Figure 12A). In contrast, neither compound **23** (40 μM) nor cisplatin (10 μM) alone was toxic to spheroids derived from A2780cis, A2780cisR, and SKOV-3 cell lines (Figure 12A). However, compound **23** together with cisplatin decreased the viability of the A2780cis and A2780cisR spheroids, thereby sensitizing them to the cytotoxicity of the drug (Figure 12A).

The 48 h treatment with cisplatin or compound **23** alone reduced cell viability only in A2780 spheroids, as described above. However, prolonged incubation (72 h) with cisplatin or compound **23** resulted in a significant decrease in the viability of the A2780cisR spheroids (*p* < 0.05, Figure 11B). Moreover, a significant effect of cisplatin with 72 h incubation was observed in A2780cis spheroids (*p* < 0.01, Figure 11B). In SKOV-3 spheroids, similar to A2780cisR, compound **23** decreased the viability when applied for 72 h (Figure 12B). The effect of metallacarborane-bearing adenosine (compound **23**) alone or in combination with cisplatin on the spheroids was assessed by comparing the spheroids to adherent cells growing in the monolayer (Figure S13A,B, Supplementary Materials). The results of two viability tests (NR and ATP measurement) are consistent: compound **23** exhibited high cytotoxicity independent of platinum resistance to adherent cells. The viability of A2780 cells (measured using ATP levels) decreased to 53 ± 5.4% after the incubation with compound **23** (40 µM, *p* < 0.001); whereas in SKOV-3 cells, the effect of compound **23** was even more apparent (viability drop to 24 ± 1.5%, *p* < 0.001, Figure S13A,B, Supplementary Materials). In combination treatment, it was found that compound **23** significantly increased the cytotoxic effect of cisplatin on cells, regardless of cisplatin sensitivity (decrease in cell viability to 26 ± 3.9% in A2780, *p* < 0.001; 46 ± 3.1% in A2780cis, *p* < 0.001; 42 ± 2.1% in A2780cisR, *p* < 0.001; 22 ± 1.5% in SKOV-3, *p* < 0.001; the RLU values are shown in Figure S13A, Supplementary Materials).

OVCAR-3 cells, similar to A2780 cells, were sensitive to cisplatin when grown in a monolayer. In contrast, unlike adherent cells, OVCAR-3 spheroids completely lost their sensitivity to cisplatin, becoming highly resistant. The effect of high doses of cisplatin on the OVCAR-3 spheroid viability is shown in Figure 12C (left). The cytotoxic effect of cisplatin on OVCAR-3 spheroids was achieved at concentrations above 50 μM, and the 50% decrease in spheroid viability was reached at a concentration of 100–110 μM. In contrast, unlike cisplatin, the toxicity of compound **23** to spheroids and adherent cells was comparably high (Figure 12C, right). Compound **23** (40 μM) was twice as toxic to spheroids as cisplatin (70 μM) (Figure 12C). OVCAR-3 cells grown in a monolayer were the most sensitive to N^6^ derivative modified with ferra bis(dicarbollides) (compound **15**), as shown in the NR assay (see Section 2.2). The IC50 of compound **15** was 21 ± 1.5 μM, and it was approximately twice lower than that of compound **23** (48 ± 2.7, *p* < 0.01, Student’s *t*-test with Bonferroni correction). Concerning these results, the effect of compound **15** on spheroid viability was also investigated in comparative studies. In contrast to the adherent cells, compound **15** was twice less toxic to OVCAR-3 spheroids than compound **23** (Figure S13, Supplementary Materials).

Compound **23** (40 μM) reduced the viability of the adherent cells by about half (A2780, A2780cis, and A2780cisR lines) or by 75% (in SKOV-3), regardless of platinum sensitivity/resistance of the cell line, whereas these cells became resistant to compound **23** when grown in spheroids (Table 2). Conversely, platinum sensitivity (A2780 cells) or resistance (A2780cis, A2780cisR, and SKOV-3 cells) remained unchanged in the spheroids in comparison to adherent cells (Table 2). OVCAR-3 cells grown in a monolayer, similar to A2780 cells, were sensitive to both compound **23** and cisplatin. However, unlike the A2780 spheroids, OVCAR-3 spheroids developed complete resistance to cisplatin. In contrast, cell sensitivity to compound **23** was maintained in the OVCAR-3 spheroids (Table 2).

### 2.11. Preparation of Metallacarborane- and Nido-7,8-Dicarbaundecaborate Derivatives

The following set of nine Co-, Fe-, or Cr-containing metalla bis(dicarbollide) derivatives of adenine nucleosides was synthesized:

- 2′-deoxyadenosine derivatives containing modification at the N^6^ position of adenine, 6-*N*-{5-[3,3′-metalla-bis(1,2-dicarba-*closo*-undekaborate)-8-yl]-3-oxa-pentoxy}-2′-deoxyadenosine, compounds **14**–**16**;
- derivatives modified at C8 position of 2′-deoxyadenosine purine system, 8-{5-[3,3′-metalla-bis(1,2-dicarba-*closo*-undekaborate)-8-yl]-3-oxa-pentoxy}-1*N*-1,2,3-triazol-4-yl}-2′-deoxyadenosine, compounds **18**–**20**; and
- derivatives of adenosine modified at C2′ of ribose moiety, 2′-*O*-{{5-[3,3′-cobalta-bis(1,2-dicarba-*closo*-undekaborate)-8-yl]-3-oxa-pentoxy}-1*N*-1,2,3-triazol-4-yl}propyloadenosine compounds **22**–**24**.

The reference compound, 2′-*O*-{[5-(7,8-dicarba-*nido*-undekaborate-10-yl)-3-oxa- pentoxy]-1*N*-1,2,3-triazol-4-yl}propyloadenosine (**25**), no-metal-ion-containing conjugate of adenosine and *nido*-7,8-dicarbaundekaborate ion, was synthesized and used as counterpart of the most active metallacarborane derivatives.

#### 2.11.1. Preparation of Dioxane Adducts

**Scheme 2:**
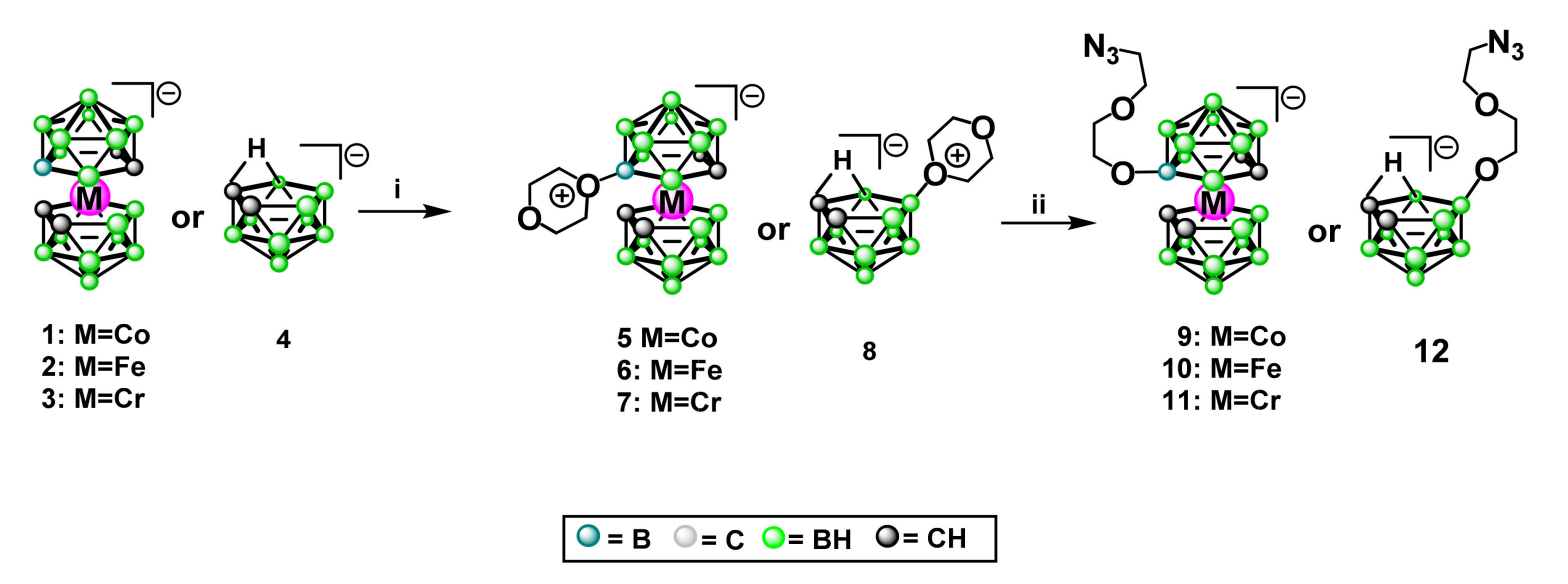
General procedure for the synthesis of 8-dioxane-[3-metalla bis(1,2-dicarbollide)] (**5**-**7**), and 10-dioxane-7,8-dicarba-*nido*-undecaborate (**8**) adducts and synthesis of 8-(5-azido-3-oxa-pentoxy)-3-metal bis(1,2-dicarba-*closo*-undekaborate](-1) (**9**-**11**) and 10-(5-azido-3-oxa-pentoxy)-7,8-dicarba-*nido*-undecaborate(-1)ate (**12**) via the dioxane ring opening in **5**-**8**: (i) 1,4-dioxane, (CHO_3_)SO_2_; (ii) NaN_3_, DMF.

**Figure 13:**
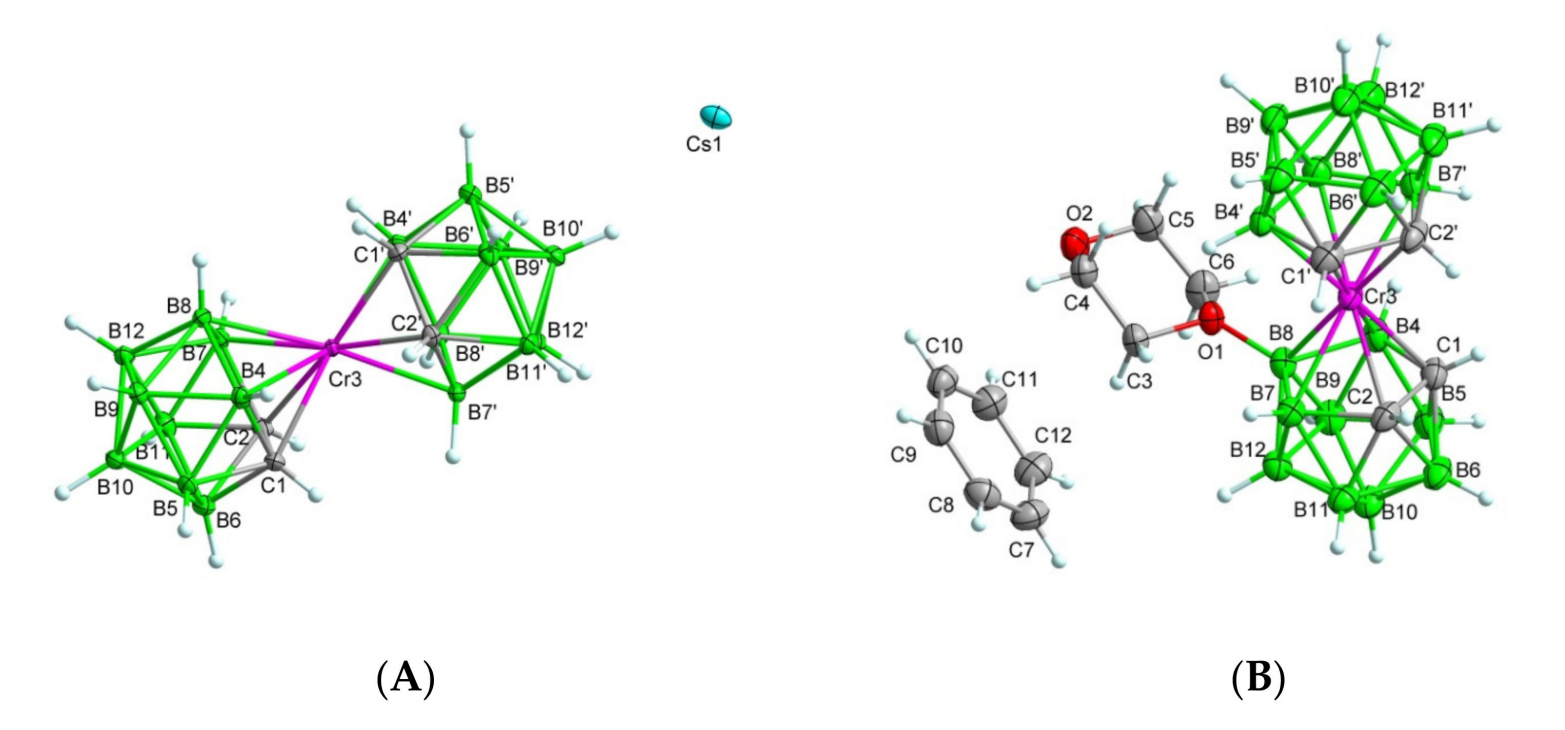
The x-ray structures of the parent chromium sandwich **3** (**A**) and the dioxane derivative **7**·C_6_H_6_ (**B**). Displacement ellipsoids are drawn at the 50% probability level. Selected interatomic distances [Å] and angles [°] are: (**A**) compound **3**, Cr3–C1 2.184(2), Cr3–C2 2.179(2), Cr3–C1′ 2.179(2), Cr3–C2′ 2.198(2), Cr3–B4 2.261(3), Cr3–B4′ 2.242(3), Cr3–B7 2.263(3), Cr3–B7′ 2.264(3), Cr3–B8 2.324(3), Cr3–B8′ 2.302(3), C1–Cr3–C2 43.31(9), C1–Cr3–C1′ 147.60(9), C2–Cr3–C2′ 146.21(9), C1′–Cr3–C2′ 42.99(9), C1–Cr3–C2′ 110.50(9); (**B**) compound **7**·C_6_H_6_, Cr3–C1 2.1910(17), Cr3–C2 2.1982(17), Cr3–C1′ 2.1789(17), Cr3–C2′ 2.1727(17), Cr3–B4 2.2540(19), Cr3–B4′ 2.2263(18), Cr3–B7 2.2669(19), Cr3–B7′ 2.225(2), Cr3–B8 2.2867(18), Cr3–B8′ 2.257(2), O1–C3 1.4889(19), O1–C6 1.4813(19), O1–B2 1.522(2), O2–C6 1.422(2), O2–C7 1.421(2), C1–Cr3–C2 42.81(7), C4–Cr3–C2 104.00(7), C1–Cr3–C1′ 134.95(7), C1′–Cr3–C2′ 43.73(7), C1′–Cr3–C2 104.50(7), C3–O1–B8 120.00(12), C6–O1–C3 111.03(12), C6–O1–B8 118.98(12).

First, the dioxane adducts were prepared as shown below (Scheme 2). The synthesis of 8-dioxane-[3-cobalta(III) bis(1,2-dicarbollide)] (**5**) [47], 8-dioxane-[3-ferra(III) bis(1,2-dicarbollide)] (**6**) [48], and zwitterions was performed according to the described procedures based on the reactions of 1,4-dioxane (used also as a reaction medium) in the presence of dimethyl sulfate, and 10-dioxane-*nido*-7,8-dicarbaundecaborate (**8**) [38] was prepared by the reaction of neutral dicarbaborane *nido*-7,8-C_2_B_9_H_13_ with dioxane as the solvent. The ring cleavage of 8-dioxane-[3,3′-chroma bis(1,2-dicarbollide)] (**7**) was shown by our group in previously published works [23,35]; however, its synthesis and characteristics are described for the first time herein (Scheme 2), inclusive of its molecular structure (Figure 13 with the description below). The reaction was performed analogously to the procedure described above with the exception that [3,3′-chromma-bis(1,2-dicarba-*closo*- undecaborate](-1)ate (**3**) was reacted with dioxane in the presence of BF_3_^.^Et_2_O instead of dimethyl sulfate [47,48]. 

8-(5-Azido-3-oxa-pentoxy)-3-metalla bis(1,2-dicarba-*closo*-undecaborates](-1) containing Co and Fe (**9** and **10**) [37,38] and 10-(5-azido-3-oxa-pentoxy)-7,8- dicarba-*nido*-undecaborate(-1) (**12**) [37,38] were obtained via dioxane ring opening in **5**, **6** or **8** correspondingly with sodium azide in anhydrous dimethyl sulfoxide as described (Scheme 2). The synthesis of counterpart containing chromium 8-(5-azido-3-oxa- pentoxy)-3,3′-chroma bis(1,2-dicarba-*closo*-undekaborate](-1) (**11**) was performed analogously from 8-dioxane-[3,3′-chroma bis(1,2-dicarbollide)] (**7**) and is described herein (Scheme 2).

Crystal structures of compounds **3** and **7:** The crystal structures of the parent chromium (III) sandwich Cs·**3** and the dioxane derivative in the form of benzene solvate **7**·C_6_H_6_ determined by x-ray diffraction analysis are depicted in Figure 13A,B (the selected interatomic distances and angles are given in the caption). For crystal packing contacts, see Figures S14 and S15 (compound Cs·**3**) and Figure S16 (compound **7**) in the Supplementary Materials. The ionic structure of the cesium salt of [3-chroma-bis(1,2-dicarba-*closo*-undecaborane](-1)ate, **3** crystallized in the monoclinic space group *P*2_1_/*n* and consisted of chroma bis(dicarbollide) anions and Cs^+^ cations. The chroma (III) cation was surrounded by two icosahedral dicarbollide ions, resulting in a sandwich-type structure (Figure 13A). The C_2_B_3_ facets were nearly parallel with a dihedral angle of 4.056°. The distances from the Cr3 to the C1–B7 and C1′–B4′ planes were 1.710 Å and 1.703 Å, respectively, similar to those observed in salts containing tetrathiafulvalenium cation [tff]^+^[Cr(C_2_B_9_H_11_)_2_]^−^ (1.69 Å, where tff = tetrathiafulvalene) [49,50].

The dicarbollide C atoms were arranged in a nearly *gauche* conformation with a (C1–C2–C1′–C2′) torsion angle of 38.94(16)°. The *gauche* conformation was probably caused by the nonbonded interactions between Cs^+^ cations and the hydrogen atoms of dicarbollide cages at distances of 2.910–3.449 Å, which was also observed in the presence of Tl^+^ cations in the Tl[3,3′-Ga(III)(l,2-C_2_B_9_H_11_)_2_] complex [51]. Each Cs^+^ cation is surrounded by six [3,3′-Cr(1,2-C_2_B_9_H_11_)_2_]^−^ anions, which are located in the intermolecular cavities between individual anionic molecules in the inversion center (see Supplementary Materials, Figure S15). Crystal data and structure refinement for compounds **3** and **7** are summarized in Table S2.

Molecular structure of 8-dioxane-[3,3-chromium-bis(1,2-dicarba-*closo*-undecaborate) (-1): Benzene solvate (**7**·C_6_H_6_), determined using x-ray crystallography (Figure 13B), revealed that the compound crystallized in the monoclinic space group *P*2_1_/*c*, and an asymmetric unit was formed by chromium(III) bis(dicarbollide) anion substituted with a dioxane molecule containing a positively charged oxonium atom (zwitterionic form) and benzene solvate. Similarly, as found in the structure of **3**, the faces of the pentagonal C_2_B_3_ were nearly parallel, with a smaller dihedral angle (2.215°). In contrast to **3**, the cages of the chroma bis(dicarbollide) ion in **7** adopted a *cisoid* conformation close to an eclipsed arrangement. Analogical coordination was observed in *closo*-[(8-(-CH_2_CH_2_O)_2_-1,2-C_2_B_9_H_10_)(1,2-C_2_B_9_H_11_)-3,3-Fe]^0^ and *closo*-[(8-O(1-CH_3_O–C_6_H_4_)-(CH_2_CH_2_O)_2_-1,2-C_2_B_9_H_10_) (1′,2′-C_2_B_9_H_11_)-3,3′-Fe], as reported by Plešek et. al. [48]. The (C1–C2–C1′–C2′) torsion angle was 37.27(13)° and the distances from the Cr·**3** to the C1–B7 and C1–B4′ planes were 1.712 Å and 1.669 Å, respectively. The compound was solvated by a benzene molecule with the shortest contact of the center of gravity (Cg) of the aromatic ring with C3-H3B hydrogen H··Cg < 3.0 Å.

#### 2.11.2. Synthesis of 2′-Deoxyadenosine and Adenosine- Metallacarborane Conjugates **14**–**24**

**Scheme 3:**
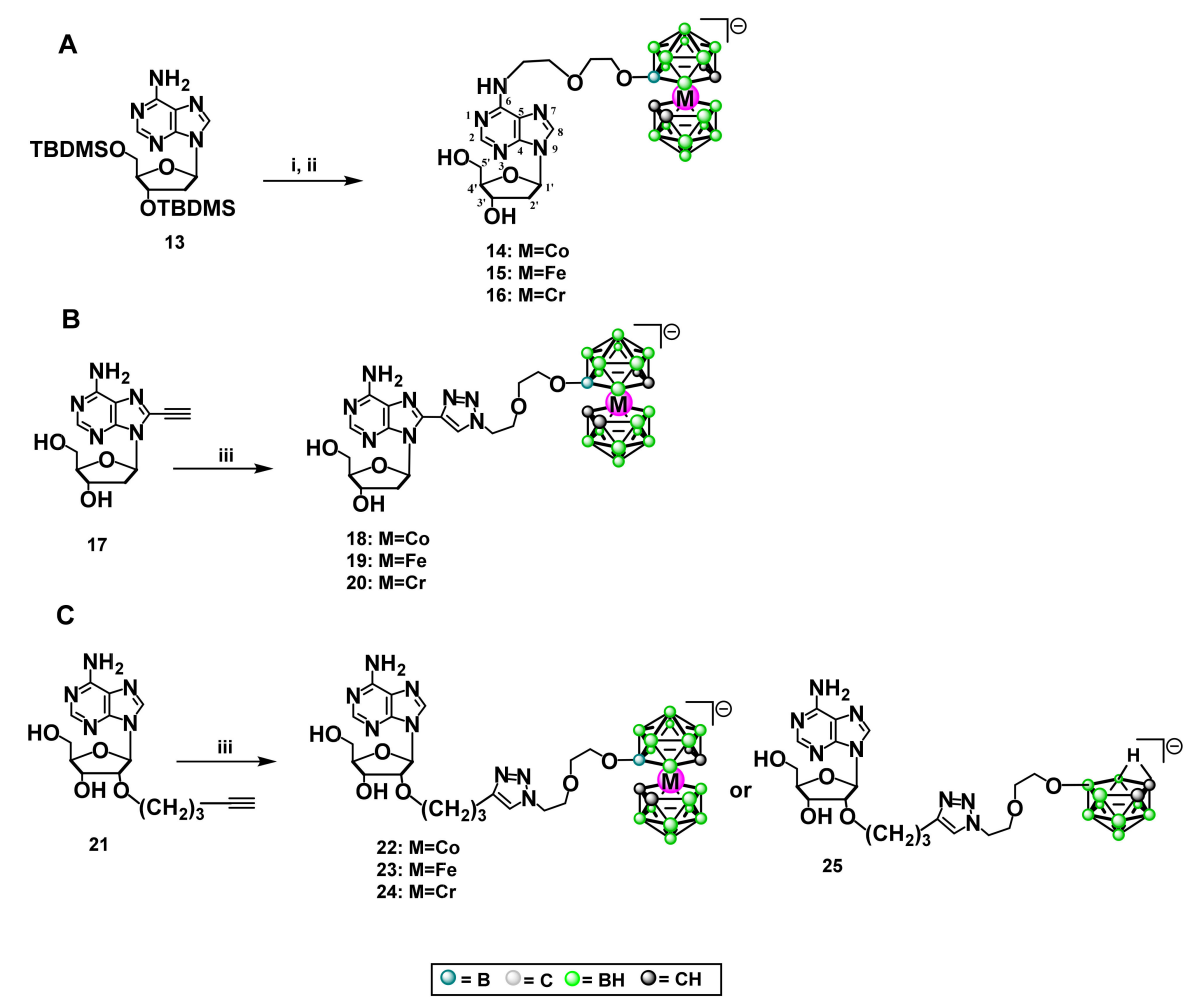
(**A**) General procedure for the synthesis of 2′-deoxyadenosine modified with metallacarborane complexes bearing Co, Fe, Cr (**14**, **15**, **16**) at position N^6^: (i) NaH/toluene, **5**, **6**, or **7**, 70 °C, (ii) TBAF/THF; (**B**) general procedure for the synthesis of 2′-deoxyadenosine modified with metallacarboranes bearing Co, Fe, Cr (**18**, **19**, **20**) at position 8: (iii) **9**, **10** or **11**, CuSO_4_ × 5H_2_O, sodium ascorbate, *tert*-BuOH/H_2_O (1:1); (**C**) general procedure for the synthesis of adenosine modified with metallacarboranes bearing Co, Fe, Cr (**22**, **23**, **24**) or *nido*-7,8-dicarbaundecaborate (**25**) at carbon 2′: (iii) **9**, **10**, **11** or **12**, CuSO_4_·5H_2_O, sodium ascorbate, *tert*-BuOH/H_2_O (1:1).

Compounds **14** [34], **15** [36], and **16** [35] modified at N^6^ were described previously and were obtained via the dioxane ring opening method [52] in a dioxane/metallacarborane adduct with an activated exo-amino group of adenine nucleobase (Scheme 3A). Briefly, first, the 5- and 3-hydroxy functions of **dAde** were protected with a *tert*-butyldimethylsilyl group, and then the -NH_2_ group at position 6 in 3,5ʹ-*O*,*O*-di(*tert*-butyldimethylsilyl)- 2′-*O*-deoxyadenosine was activated with sodium hydride and then treated with an excess of suitable dioxane/metallacarborane adduct **5**–**7**. Then, the *tert*-butyldimethylsilyl protection was removed by treatment with tetrabutylammonium fluoride to provide compounds **14–16**.

Compounds **18–20** modified at C8 were prepared via the “click chemistry” approach [37,38,53] (Scheme 3B). The boron cluster acceptor, 8-ethynyl-2′-deoxyadenosine (**17**), bearing a triple bond at carbon 8, was obtained according to the literature method [54]. Next, **17** was dissolved in butanol and water, together with an equimolar amount of a suitable metallacarborane containing Co, Fe, or Cr equipped with a 3-oxa-pentoxy spacer terminated with an azido group, compounds **9**, **10**, and **11**. Catalytic amounts of CuSO_4_ and potassium ascorbate solutions were added to the mixture obtained. Reactions were performed at room temperature over 8–50 h (usually 24 h) with a TLC control. After reaction completion, the solvents were evaporated and the crude products were purified silica gel column chromatography. The yields of the purified products **18–20** ranged from 30–65%.

The adenosine derivatives **22–24** modified with metallacarborane containing Co, Fe, or Cr at carbon 2′ were also obtained via the “click chemistry” method (Scheme 3C), analogously as described above with the difference that 2′-*O*-(1-pentyn-5-yl)adenosine (**21**), bearing a terminal triple bond, was used as a boron cluster acceptor in the “click reaction” [55]. The derivative bearing *nido*-7,8-dicarbaundecaborate modification (25) was obtained as previously described [32].

## 3. Discussion

Overcoming drug resistance in refractory or recurrent ovarian cancer in chemotherapy is based on multi-drug regimens; usually platinum-based therapy is used in combination with other drugs for the treatment of partially- or highly-resistant tumors [56]. However, the therapeutic effect is short-lived and relapse often occurs. Taking into consideration the unique physicochemical properties of metallacarboranes as modifiers of adenine nucleosides, the metalla bis(dicarbollide)-modified adenosine and 2′-deoxyadenosine derivatives studied herein that were found to exert antitumor activity may be promising candidates as drugs for cancer therapy [18]. The following set of new and already published adenosine derivatives with Fe-, Co-, or Cr-containing metallacarboranes attached at the N^6^ and C8 positions of nucleobases or at the C2′ position of the ribose moiety was synthesized and evaluated against ovarian cancer cells. The adenosine modified with the *nido*-7,8-dicarbaundecaborate ion at C2′ position, bearing a boron cluster but not a metal, was obtained as a counterpart of the lead compound and used for comparison with metallacarborane modifications. Sodium salts of parent metallacarboranes containing Fe, Co, or Cr ions were prepared and used as reference compounds; unmodified adenine nucleosides were also used for comparison. Cisplatin-sensitive and cisplatin-resistant ovarian carcinoma cell lines were used to determine the anticancer activity of these compounds and evaluate the mechanism underlying the action of these compounds. These cell lines included commercial cell lines with differing sensitivity to platinum drugs (A2780, A2780cis, SKOV-3, and OVCAR-3) and experimental cell lines ‘not responding’ to carboplatin and highly resistant to cisplatin with an IC50 2–3 times higher than that of the parental A2780cis resistant cell line and the resistance index 9–13 with respect to the sensitive A2780 cell line. The resistant cells were comparably sensitive than the other cell lines to the cytotoxic activity of Fe-containing compounds, which had higher impact on the cell viability than carboplatin. In general, the compounds, depending on the metal ion and substitution site, increased the sensitivity of resistant cells to cisplatin by cell cycle arrest, increasing apoptosis and necrosis, and the highest ROS formation. Interestingly, unmodified nucleosides or the adenosine modified with the *nido*-7,8-dicarbaundecaborate ion were ineffective on cancer cell biology.

**Figure 14:**
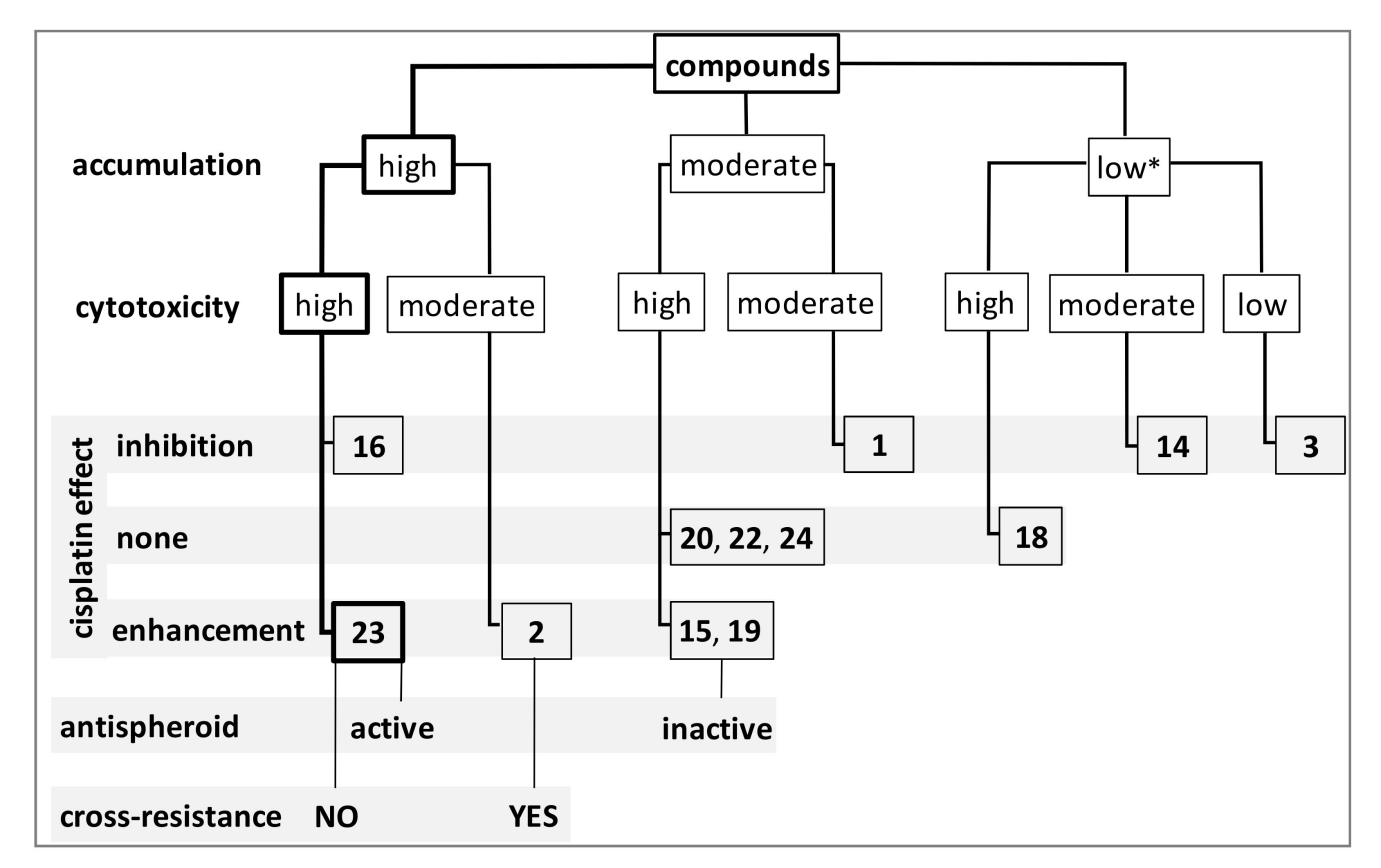
Selection of the lead compound **23** with the best anticancer activity: high intracellular accumulation, high toxicity against cisplatin-resistant cells, increase in the cytotoxic effect of cisplatin on cisplatin-resistant cells, active against cancer spheroids, and does not induce cross-resistance to anticancer drugs (cisplatin, carboplatin, paclitaxel, doxorubicin, and gemcitabine). Inhibitor—compound decreasing cisplatin effects; none—not active compound; amplifier—compound augmenting cisplatin effect; low*—relatively low accumulation, twice as high as cisplatin.

A broad cell-based screening of the derivative properties and evaluation of their mechanisms of action led to the identification of the lead compound active against ovarian cancer cells. A summary of the selection of the lead compound is shown in Figure 14.

The C2′ adenosine derivative modified with ferra bis(dicarbollide) ion (**23**) had the best spectrum of anticancer activity including high intracellular accumulation, comparable and efficient toxicity against cancer cells resistant and sensitive to platinum drugs, proapoptotic activity, and ability to augment cisplatin sensitivity of the resistant cell lines. In addition, a group of compounds with moderate or relatively low intracellular accumulation, which did not affect or slightly enhance the effect of cisplatin, was identified (**3**, **14**, and **25**). Their activity profile indicates that they are possible boron-carrier chemotherapeutics suitable for boron neutron capture therapy [28,57]. The higher the accumulation of the compounds, the higher the cytotoxic effect, as revealed by the majority of the compounds studied, with few exceptions. Nevertheless, measured in terms of nmol, the accumulation of the least penetrating compounds still exceeded twice that of platinum. Our study has shown that metallacarborane derivatives accumulate in cells and they affect cell viability as well as they inhibit cell proliferation during long-term treatment. We presuppose that metallacarboranes, considered as chemically inert toward biological systems [58], can “trick” the neoplastic cell without even being recognized as xenobiotics, therefore they can persist inside the cell. However, further studies are needed to explain this phenomena. It has been previously reported by others that some derivatives of carboranes are uptaken by cancer cells [21,59]. Moreover, the unmodified cobalt bis(dicarbollide) anion readily accumulates inside cells and is retained for at least four hours inside the cell, as has been shown by νb-H Raman spectroscopy [60]. It is possible that inside the cell, metallacarborane derivatives of adenosine can interact with cellular membrane, lipid bilayer, or DNA, analogously to other classes of carborane or metallacarboranes reported by Murphy et al., Fuentes et al., or Rokitskaya et al. [59,61,62]. Nevertheless, it can be expected that carborane compounds are poorly metabolized in the body, since no metabolites of carborane-modified biomolecules have been observed in serum by LC-MS and HPLC [21].

The analysis of the effects of the compounds on cell viability and the ability to accumulate in cells was performed with special focus on the type of metal ion in the cluster and the position of modification in adenine nucleosides as well as the importance of the presence of a nucleoside in conjunction with a metalla bis(dicarbollide) ion. The viability of ovarian cancer cells was significantly disturbed by the C2′ and C8 derivatives, and the N^6^ derivatives containing Fe and Cr ions, regardless of the cellular resistance to platinum. Unmodified metalla bis(dicarbollide) ions containing Co and Fe ions (**1**, **2**) had a moderate effect on cell viability, and the Cr-containing parent sandwich (**3**) was inactive. Among the compounds that had a weak or moderate effect on cancer cell viability, a Co-containing N^6^ derivative was found (**14**). Unlike all other derivatives, compound **14** had an even lower effect on some cisplatin-resistant lines than the corresponding unmodified cobalta bis(dicarbollide) ion (**1**). Overall, N^6^ derivatives were the most diverse group of compounds with cytotoxic effects, in contrast to the C2′ and C8 derivatives, which were similarly potent. Among the analyzed compounds, derivative **23** was the most effective in inducing apoptosis in ovarian cancer cells. In the 48 h combination treatment, compound **23** increased the proapoptotic effect of cisplatin on cisplatin-resistant A2780cis cells. In addition, the use of **23** in combination with cisplatin resulted in an almost two-fold increase in ROS levels in cells pre-incubated with compound **23** compared to that of the action of cisplatin alone. Cells incubated with compound **23** and cisplatin in combination also showed significantly greater apoptosis than cells incubated with cisplatin alone. The unmodified ferra bis(dicarbollide) ion (**2**) also enhanced ROS production and apoptosis in cisplatin-treated A2780cis cells. Nevertheless, **2** in long-term cultures induced cross-resistance in the cells to the platinum drugs, PTX, DOX, and gemcitabine, whereas compound **23** did not change cellular sensitivity to the drugs.

Compound **23**, similar to cisplatin, but unlike **2**, induced a high and significant increase in p53 expression and its phosphorylation and acetylation. P53 is a key regulator of cellular stress response, arrest in the G1 phase of the cell cycle, and apoptosis mechanisms in response to damage. Activation of p53 and its phosphorylation at Ser15 and Ser37 and acetylation play a positive role in the accumulation of p53 protein in stress response, which leads to apoptosis and inhibition of the cell cycle, which is true for wt p53. The A2780 and A2780cis cell lines contained wt p53. The functional relationship of p53 with induction of apoptosis has been shown in cisplatin-treated A2780 cells by other authors [63]. In contrast, an increase in expression of the p53 mutant (in OVCAR-3 and SKOV-3 cells) may be ineffective in triggering apoptotic processes due to the dysfunction of the mutant protein. In the context of p53 activation and its post-translational changes, compound **23** was similarly effective as cisplatin, in all ovarian cancer cells, which is of particular importance for A2780 and A2780cis cells expressing wt p53. However, in the context of the cytotoxic efficacy of compound **23** in OVCAR-3 and SKOV-3 cells, the significance of mutant p53 induction in apoptosis and inhibition of the cell cycle in these cells might be considered in additional studies. In contrast to p53, transcription factors AP-1 and NF-κB play crucial roles in cell survival, mainly in promoting cancer progression [64]. In our study, compound **23**, similar to cisplatin, inhibited NF-κB promoter activity, without significantly affecting AP-1 activity. In summary, compound **23** induced cell apoptosis and enhanced the cisplatin effect with a parallel increase in p53 activation and inhibition of NF-κB activity, which indicates its desirable proapoptotic mechanism of action.

Although monotherapy for ovarian cancer is beneficial in the initial stages of treatment, multidrug resistance develops over time, resulting in cancer recurrence. A parallel process of acquiring cell resistance to chemotherapeutic agents has also been observed in cell cultures. This phenomenon was used to obtain cultures of resistant or cross-resistant cells to the given drugs. In our study, we used the phenomenon of induced drug resistance to obtain the A2780 cell subline, which is highly resistant to cisplatin, and we used this model to test the cytotoxicity of our compounds. In contrast, we examined whether compound **23**, identified as promising in our research, did not cause cross-resistance to other chemotherapeutic agents; in other words, whether it met the criterion of a safe compound for long-term use.

In patients who are refractory or resistant to platinum drugs, the standard approach of chemotherapy for epithelial ovarian cancer is the combination of two drugs (e.g., carboplatin and paclitaxel or docetaxel) [1]. In this study, we showed how carborane-nucleoside derivatives modulate the effects of cytostatics on cancer cells. The anticancer drugs were selected due to their therapeutic efficacy and their heterogeneous mechanism of action (carboplatin, cisplatin, paclitaxel, docetaxel, doxorubicin). Therefore, we examined whether chronic exposure to compound **23** is safe for cells, that is, does not induce cross-resistance to these chemotherapeutics. Our results showed that long-term culture of platinum-resistant cells, A2780cisR with compound **23**, did not alter cell sensitivity to PTX, DOX, GEM, cisplatin, or carboplatin. The presence of nucleosides in the structure of **23** was revealed to be indispensable for protecting cells against the development of drug resistance, as demonstrated by the results for the unmodified ferra bis(dicarbollide) ion. The A2780cisR cells cultured for several cell cycles in the presence of compound **2** decreased their rate of division, but developed resistance to all the drugs tested, PTX, DOX, GEM, cisplatin, and carboplatin. Apoptosis and necrosis were completely abrogated in cells cultured for several weeks with the addition of compound **2** to the culture medium, followed by subsequent treatment with PTX, DOX, GEM, cisplatin, or carboplatin. Interestingly, the *nido*-7,8-dicarbaundecaborate derivative **25**, similar to **23**, did not change the cellular response to the drugs. Nonetheless, compound **25**, unlike compound **23**, did not affect apoptosis or cell viability. The results obtained for reference compounds **2** and **25** and for test compound **23** show that only the combination of a nucleoside with a metallacarborane protects cells from developing multidrug cross-resistance while exerting proapoptotic effects.

Another model in which we investigated the anticancer properties of the compounds was 3D spheroids. In the abdominal cavity of patients with advanced-stage ovarian cancer, single cancer cells are suspended in the peritoneal cavity fluid (ascites), whereas multicellular aggregates cover the peritoneum. Spheroidal aggregates show increased survival capacity, invasiveness, and resistance to chemotherapy ex vivo, in comparison to single cancer cells. Three-dimensional spheroid cell cultures are a particularly useful model for in vitro studies of cancer cell chemoresistance. They are the most metastatic structures in in vivo studies when administered subcutaneously, readily and efficiently forming tumors in mice. However, generating these structures requires the adjustment of culture conditions to the individual requirements of a given cell line or the cells of a given type of cancer. Spheroids were derived from the ovarian cancer cell lines A2780, A2780cis, A2780cisR, SKOV-3, and OVCAR-3. Their responses to metallacarborane-bearing adenosine derivative 23, alone or in combination with cisplatin, were determined using an ATP viability assay intended for 3D large structures.

It is known that OVCAR-3 loses its resistance in adhesive cultures in vitro. Similarly, in our adhesion cultures, OVACR-3 cells showed a similar response to platinum drugs as the A2780 cells, which were considered sensitive. Therefore, in our study, we classified adhesive OVCAR-3 cells growing in a monolayer as relatively sensitive. However, in our experiments, we showed that OVCAR-3, which had lost its cisplatin resistance in adhesive cultures in vitro (IC50 5 μM), recovered it in 3D spheroids and became highly resistant to cisplatin (IC50 70 μM). It has been previously reported by others that compact spheroid formation by ovarian cancer cells is associated with invasive phenotype [65]. Interestingly, compound **23** alone was twice as cytotoxic as cisplatin in OVCAR-3 spheroids in our study. Moreover, compound **23** enhanced the cisplatin effect in the spheroids, but this effect was additive rather than synergistic, indicating independent pathways of action of both compounds. Compound **23** also had a noticeable cytotoxic effect on the large mature spheroids of A2780 cells, where the ability of compound **23** to penetrate the interior of the spheroid was demonstrated. The A2780 cells within the interior of the spheroid underwent necrosis without disintegration of the entire structure. In contrast, cisplatin attacked mainly external cells, causing cell spheroid degradation. The results obtained suggest that compound **23** penetrates the spheroid structure differently than cisplatin when we compared the mechanism of penetration into the spheroid, not the mechanism of intracellular action. The structure of the spheroid is important for the cytotoxic activity of compound **23**. While compound **23** was less effective on loosely structured spheroids, regardless of their platinum resistance (A2780 sublines and SKOV-3), it acted twice as effective on compact but platinum-resistant spheroids (OVCAR-3). Furthermore, ex vivo studies may provide a comprehensive answer to the question of whether compound **23** or other nucleoside-functionalized metallacarboranes have the ability to penetrate tumor tissue. However, the position of nucleoside modification and type of metal seem to be important for antispheroid activity because other derivatives, cytotoxic for adherent cells, have not been effective in the spheroids built up from these cells (**15** and **24**, Fe- or Cr-containing N^6^ and C2′ derivatives).

In summary, compound **23** [a conjugate of adenosine and ferra bis(dicarbollide) ion] showed high intracellular accumulation, toxicity against both cisplatin-sensitive and cisplatin-resistant cancer cells, and against cancer spheroids. It enhanced cisplatin proapoptotic effects in cancer cells, and did not induce cross-resistance to standard anticancer drugs. It is also worth noting that although unmodified ferra bis(dicarbollide) ion **2**, which has good intracellular accumulation, sensitized cells to cisplatin treatment in short-term treatment, it induced cell resistance to anticancer drugs in the long-term period of the cell culture. In contrast, the *nido*-7,8-dicarbaundecaborate derivative **25** did not change the cellular response to the drugs. Nonetheless, compound **25**, unlike compound **23**, did not affect apoptosis or cell viability.

## 4. Materials and Methods

### 4.1. Chemicals

All chemicals for synthesis were used without further purification unless otherwise stated. The following were purchased from Sigma-Aldrich (St. Louis, MO, USA): N,N-dimethylformamide anhydrous 99.8%, ethynyltrimethylsilane, acetic acid (glacial) 100%, 2-propanol 99.5%, tetrakis(triphenylphosphine)palladium(0) 99%, and 5-chloro-1-pentyne 98%. Acetonitrile 200 SpS far UV 99.9% (H-048) was purchased from ROMIL (Cambridge, UK). Pyridine dried (max. 0.0075% H_2_O) SeccoSolv > 99.9 and N,N-dimetyloformamide EMPLURA Supelco, and dimethyl sulfoxide (DMSO) EMPLURA were purchased from Merck (Darmstadt, Germany). ACROS Organics: *tert*-butylchlorodimethylsilane 98%, sodium hydride 60% dispersion in mineral oil, and 1,4-dioxane 99.8% extra dry were purchased from Thermo Fisher Scientific (Waltham, MA, USA). Alfa Aesar: 8-bromo-2′-deoxyadenosine 99% and 1,3-Dichloro-1,1,3,3-tetraisopropyldisiloxane 97% were purchased from Thermo Fisher GmbH (Kandel, Germany). C_2_B_10_H_12_, Cs[Co(C_2_B_9_H_11_)_2_] COSAN, and Cs[Fe(C_2_B_9_H_11_)_2_] FESAN were purchased from Katchem spol. s r. o. (Prague, Czech Republic). Adenosine free base 99% was purchased from BioSchop Canada Inc. (Burlington, ON, Canada). 2′-Deoxyadenosine min 99% was purchased from Carbosynth (Compton, UK). Extra-pure nitric acid (ROTH-X898.1) was purchased from Carl Roth GmbH Co. (Karlsruhe, Germany). Silica gel column chromatography was performed on silica gel 60 (230–400 mesh), Sigma-Aldrich (Steinheim, Germany) or Merck (Darmstadt, Germany). Rf values refer to analytical TLC performed using pre-coated silica gel 60 F254 plates purchased from Sigma-Aldrich (St. Louis, MO, USA) or Merck (Darmstadt, Germany) and developed in the solvent system indicated. Flash columns for HPLC, PURIFLASH C18-HP 15 μm F0004 were purchased from Interchim (Montlucon, France).

### 4.2. Cell Culture Reagents

The following were purchased from Corning Life Sciences (Tewksbury, MA, USA): Roswell Park Memorial Institute (RPMI) 1640 with l-glutamine (10-040-CVR); Dulbecco’s modified Eagle medium (DMEM; 4.5 g/L glucose, l-glutamine, and sodium pyruvate; 10-013-CVR), Ham’s F-12 medium with l-glutamine (10-080-CV), penicillin/streptomycin (P/S) solution 100× (30-002-CI), fetal bovine serum (FBS; 35-079-CV); phosphate-buffered saline (PBS) 10× concentrate pH 7.4 (PBS; 46-013-CM), Dulbecco’s phosphate-buffered saline (DPBS) 10× (DPBS; 20-30-CVR), Hank’s balanced salt solution ×10 (HBSS; 20-023-CVR), 45% glucose solution (25-037-CIR), and trypsin/0.53 mM EDTA)in HBSS (25-052-CV). The following were purchased from Sigma-Aldrich (St. Louis, MI, USA): 3-(4,5-dimethylthiazol-2-yl)-2,5-diphenyltetrazolium bromide (MTT); bisBenzimide H (Hoechst) 33258; trypan blue; water for molecular biology (95284); a Total Protein Kit (micro-Lowry, Onishi & Barr modification); and media for initiation of A2780cis cell culture: RPMI-1640 medium, w/o l-glutamine (R0883), l-glutamine solution bioxtra (G7513), FBS (F2442), and trypsin-ethylenediaminetetraacetic acid (EDTA) solution 0.25% (T4049). RPMI-1640 ATTC medium (ATCC-30-2001) and FBS (ATCC-30-2025) for the initiation of OVCAR-3 cell culture were purchased from LGC Standards (Teddington, UK). Sterile DMSO for cell freezing medium was purchased from BioSchop Canada Inc. (Burlington, ON, Canada). BD Cytofix/Cytoperm and fixation/permeabilization kit (554714) were from BD Bioscience (San Jose, CA). RPMI 1640 ATCC modification (A1049101), HBSS 1x with calcium and magnesium without phenol red (14025100), 2′,7′-dichlorofluorescin diacetate (DCF-DA), and 5-chloromethylfluorescein diacetate (CMFDA, CellTracker Green) were purchased from Thermo Fisher Scientific (Waltham, MA, USA). Propidium iodide (PI) was purchased from Carbosynth (Compton, UK); the purity of the compounds was ≥98%. Bis(1,3-dibutylbarbituric acid)trimethine oxonol (DiBAC4(3)) was purchased from ATT Bioquest (Sunnyvale, CA, USA). 3-Amino-7-dimethylamino-2-methylphenazine hydrochloride (neutral red) was purchased from Abcam (Cambridge, UK). Annexin V–fluorescein isothiocyanate (FITC) was obtained from BD Bioscience (Franklin Lakes, NJ, USA). The purity of compounds used for cell culture treatment was ≥98–100%.

#### 4.2.1. P53 Antibodies

The FITC anti-p53 monoclonal antibody set (554298, clone DO-7 recognizing the human wild type and mutant p53, and clone 27-35 isotype control) were from BD Bioscience (San Jose, CA, USA). The following were from Miltenyi Biotec (Bergisch Gladbach, Germany): FITC anti-p53 human clone: REA609 (130-109-569; recognizes the human cellular tumor antigen p53), anti-p53 pS15-APC human clone: REA825 (130-112-621; recognizes the human cellular tumor antigen p53 phosphorylated at serine 15), anti-p53 pS37-APC human clone: REA424 (130-106-599; recognizes the human cellular tumor antigen p53 phosphorylated at serine 37), anti-p53 acK382-PE human clone: REA529 (130-109-072; recognizes the human cellular tumor antigen p53 acetylated in the C-terminal region at lysine 382), and recombinant human IgG1 isotype control antibodies FITC-, APC-, or PE-conjugated (130-113-437, 130-120-709, and 130-104-613, respectively).

#### 4.2.2. Drugs

The following chemotherapeutics were purchased from AK Scientific Inc. (San Francisco, CA, USA): paclitaxel (99%; A648), carboplatin (99%; H876), and gemcitabine HCl (98%; F287). *cis*-Diammineplatinum(II) dichloride (cisplatin, 99.99% P4394) and gemcitabine hydrochloride European Pharmacopeia (EP) Reference Standard were purchased from Sigma-Aldrich (St. Louis, MO, USA). Doxorubicin HCl (99%; DOX042) was purchased from BioSchop Canada Inc. (Burlington, ON, Canada).

#### 4.2.3. Preparation of Stock Solutions of Drugs and Compounds for Biological Study

The doxorubicin HCl, gemcitabine HCl, and carboplatin were dissolved in H_2_O at 25 mM concentration. The paclitaxel was dissolved in DMSO at 2.5 mM. Cisplatin was dissolved in H_2_O (1 mM) or DMF (50 mM); the type of solvent did not affect the cytotoxicity of cisplatin at the concentrations used. The 2′-deoxyadenosine, adenosine, 2′-deoxyadenosine derivatives, and metallacarboranes were dissolved in DMSO or DMF at 100 mM. All ionic compounds were used in the form of their respective sodium salts. The compounds were stored in aliquots at −20 °C until used. The stock solution was next diluted with a culture medium prior to use to obtain a solvent (vehicle) concentration below or equal to 0.06% without loss of cellular viability. The dilutions were performed in one step by direct dropwise addition of a specified volume of the stock to the final volume of media, with vigorous shaking. This procedure guaranteed clear solutions of the compounds.

### 4.3. Cell Lines and Growth Conditions

The following cell lines were used for the study:

- A2780 cell line (ECACC 93112519, Sigma-Aldrich), parental cisplatin-sensitive;
- A2780cis cell line (ECACC 93112517, Sigma-Aldrich), cisplatin-resistant;
- A2780cisR and A2780cisKB cell lines of increased platinum resistance or refractory, obtained experimentally for the purpose of this study;
- SKOV-3 stable cell line, cisplatin-resistant; and
- NIH:OVCAR-3 ovarian adenocarcinoma cell line (ATCC-HTB-161; LGC Standards, Teddington, UK), relatively cisplatin-resistant/sensitive.

The OVCAR-3 cell line was established from the malignant ascites of a patient with progressive adenocarcinoma of the ovary that was refractory to cisplatin, but OVCAR-3 exhibited low resistance in in vitro culture [39,40]. All the cell lines were cultured under standard cell culture conditions, that is, at 37 °C and under 5% CO_2_ in a humidified atmosphere. After the cells reached 70–80% confluency, they were passaged in a ratio of 1:5 to 1:32, depending on the growth rate of the cell line. The viability of trypsinized cells was determined using trypan blue staining. The cells were free of mycoplasma, and they were harvested in the exponential growth phase and left to settle for 24 h before treatment. Routine screening of cell cultures for mycoplasma contamination was performed using the MycoProbe Mycoplasma Detection Kit (CUL001B; R&D Systems, Minneapolis Canada). Cells were collected once every 3–4 months, frozen (10% DMSO in the relevant medium), and stored in liquid nitrogen. Cell line authentication involving short tandem repeat profiling (STR) was performed by the European Collection of Authenticated Cell Cultures (ECACC) and Eurofins Genomics Services. The cells were collected for experiments by trypsinization, washed with culture medium (200× *g*, 5 min), and then transferred in the appropriate culture medium to 96-, 24-, or 6-well plates at densities of 1 × 10^4^, 3.5 × 10^5^, and 5 × 10^5^ cells per well, respectively, depending on the experiment. The OVCAR-3 cell line was cultured in high-glucose RPMI 1640 (ATCC modification medium) with 10% FBS and 100 μg/mL penicillin/streptomycin. A2780, A2780cis, and SKOV-3 were grown in standard RPMI 1640 culture medium supplemented with 10% FBS and 100 μg/mL penicillin/streptomycin. Cisplatin was added at a concentration of 1 μM to the A2780cis cell line every two passages to maintain drug resistance. Experimental A2780cisR and A2780cisKB lines were derived from the A2780cis cell line. A2780cis cells were cultured three months with cisplatin; the concentrations were gradually increased from one to five or 15–20 μM. The resulting cell line with high refractoriness to cisplatin was evaluated using STR DNA profiling (subcontract ECACC service). A2780cisR and A2780cisKB cell lines were cultured one week without cisplatin before an experiment.

### 4.4. Determination of Compound Cytotoxicity

Two tests were initially used to assess cell viability: the MTT test, which is based on mitochondrial oxidoreductase activity, and the neutral red incorporation test (NR), which is independent of metabolism and is based on the incorporation of the neutral red dye into lysosomes. Neutral red is a supravital dye that is taken up via active transport into live cells and is preferentially located in lysosomes [66]. The neutral red test was selected for further experiments because of its independence from mitochondrial redox activity (on which the MTT test is based). The MTT assay was performed as described in the Supplementary Materials in some experiments with drugs for comparison with NR results.

Twenty-four hour cell cultures (initial cell amount, 10^4^ per well) were treated with adenosine derivatives (**14**, **15**, **16**, **18**, **19**, **20**, **22**, **23** and **24**), or reference compounds: **1**, **2**, **3**, **25**, **Ade**, **dAde** for 48 h (37 °C and 5% CO_2_ in humidified atmosphere) at concentrations ranging from 0.01 to 200 µM (0.01, 0.1, 1, 5, 10, 50, 100, and 200 µM). The cisplatin and carboplatin concentration ranges were adjusted according to cell line sensitivity (0.001–100 µM, and 0.01–200 µM, or 0.1–300 µM). In the control experiments, solvent was added to the cell culture instead of the compound. The highest concentration of DMSO (or dimethylformamide [DMF] for cisplatin experiments) used as the vehicle control was below 0.2% (*v*/*v*), which did not affect the cell functions tested. In the 50% cytotoxic concentration (IC50) range of the compounds, the solvent concentration did not exceed 0.05–0.06%. Cytotoxic concentrations (IC50) were separately calculated for each experiment by using GraphPad Prism 6.01 software (San Diego, CA, USA) and AAT Bioquest server tools (Sunnyvale, CA, USA). The mean ± standard error of mean (SEM) values were calculated for at least three independent experiments carried out on two to three separate passages per day for each compound and each cell line. Sensitivity to cisplatin was evaluated every two weeks and was stable during the experiments performed on a given cell line. The cell viability (IC50) was determined by using neutral red assay (NR) as previously described [17]. Methodology is described in the Supplementary Materials.

### 4.5. Accumulation of Compounds in Cells

The intracellular levels of the compounds were measured in terms of the B, Fe, Cr, and Co content in lysates of cells incubated with metallacarborane compound compounds by using inductively coupled plasma mass spectrometry (ICP-MS). Pt was measured for comparison in cells incubated with cisplatin as described [17]. The 24-h A2780 and A2780cis cell cultures in 6-well plates (1 × 10^6^ cells per well, 37 °C, 5% CO_2_) were exposed to the compounds (80 μM) or cisplatin for 24 h (37 °C, 5% CO_2_). The incubation period, cell counts, and compound volume and concentration were established in an initial experiment. The cells were then washed gently with sterile warm PBS and collected by trypsinization and centrifugation (200× *g*, 5 min). Subsequently, the cell pellets were washed three times with sterile PBS and finally mineralized with 2 mL 70% HNO_3_ overnight at ambient temperature (20–22 °C). The element concentration (B, Fe, Cr, and Co) was measured with the ICP-MS method on a NexION 300D spectrometer (Perkin Elmer Inc. Shelton, CT, USA) and expressed in terms of ppm/mg of cell proteins and ppm/10^6^ cells. The amount of protein in the cell lysates was determined with the micro-Lowry method and the Onishi and Barr modification (Total Protein Kit). Cell lysates for protein content analysis were obtained from the same probe by repeatedly freezing and thawing the samples and by sonication for 1 min (Sonorex Digitec DT 31H, Bandelin electronic GmbH & Co. KG, Berlin, Germany).

### 4.6. Apoptosis, Necrosis, and ROS Production

A2780cis cells were seeded into 6-well plates at a density of 3.5 × 10^5^ cells and cultured for 24 h before treatment (37 °C and 5% CO_2_). Next, the cells were treated with the compounds alone for 48 h; in some experiments, cisplatin was added (10 μM) after 3-h treatment with the compounds. The concentration of the compounds was lower than or equal to the IC50 for compound cytotoxicity, determined earlier for individual cell lines. Cell death was determined in terms of necrosis and apoptosis of cells stained with PI and annexin V-FITC. The cells cultured for 24 h in 6-well plates (5 × 10^5^ cells per well, 37 °C, and 5% CO_2_) were treated with the compounds alone for 48 h; in some experiments, cisplatin was added (10 mM) after 3-h treatment with the compounds. Control cells were not treated or treated with a single compound in two-drug combination experiments. The cisplatin concentrations for the different cell lines were adjusted to the cisplatin sensitivity, that is, to the half-maximal effect on cell viability.

#### 4.6.1. PI/Annexin V Assay

After treatment, the cells were collected by trypsinization and stained with annexin V-FITC and PI as described earlier [17]. The cells were analyzed by flow cytometry within 30 min on an LSRII BD cytometer equipped with the FACSDiva software (BD Dickinson, BD Biosciences, San Jose, CA, USA) with an excitation wavelength of 488 nm. Emission was measured in the green and red fluorescence channels (FL1 and FL3, respectively). PI incorporation into cells (necrosis) without annexin V-FITC binding to the cellular membrane indicated necrotic cells, annexin V-FITC binding without PI staining indicated early apoptosis, and annexin V-FITC plus PI staining indicated late apoptosis, that is, necrosis as a final result of apoptosis.

#### 4.6.2. DCF-DA Cellular-Based Assay

The DCF-DA (2′,7′-dichlorofluorescin diacetate) was used to measure the ROS concentration in cells that were either untreated or treated with compounds. After DCF-DA diacetate crossed the membrane, it de-esterifies to DCFH, which is oxidized to fluorescent DCF by the ROS. Cells were incubated with DCF-DA in HBSS (10 μM, 3 × 10^5^ cells in 0.3 mL, 30-min incubation at 37 °C). The fluorescence intensity was determined using an LSRII BD cytometer with an excitation wavelength of 488 nm and emission in the green fluorescence channel FL1 and expressed as median fluorescence intensity (MFI).

#### 4.6.3. Plasma Membrane Potential

The plasma membrane potential (Δψ_p_, PMP) was also determined in the same cell samples. Δψ_p_ was measured using flow cytometry analysis involving the fluorescence intensity of bis-barbituric acid oxonol (DiBAC4(3)), a standard potentiometric probe for this purpose [67]. The Δψ_p_ changes were interpreted as a degree of plasma membrane damage since decrease in transmembrane potential accompanies disruption of the cell membrane. When the plasma membrane is depolarized, its permeability increases and the dye penetrates through the membrane, leading to enhanced green fluorescence. Cells were incubated with DiBAC4(3) in HBSS (10 nM, 3 × 10^5^ cells in 0.3 mL, 30-min incubation at 37 °C) as described earlier [17]. DiBAC4(3) fluorescence intensity in individual cells was measured within 15 min in LSRII BD cytometer with an excitation wavelength of 488 nm and emission in the green fluorescence channel (FL1) and expressed as median fluorescence intensity (MFI). The reference experiment demonstrating a decrease in plasma membrane potential in A2780cis cells in response to the toxic effect of sodium azide is shown in the Supplementary Materials (Figure S3B).

### 4.7. Cell Cycle

After the cells were treated as described above, the collected and washed cells were fixed with ice-cold 70% ethanol and stored at −20 °C. Before fixation, the number of cells was estimated by using a Thoma cell counting chamber (Marienfeld, Germany). Before staining, the cells were washed with cold PBS, suspended in fresh PBS, and left to reach ambient temperature (20–22 °C). Cells were stained with PI (50 μg/mL) in PBS containing RNase A (200 μg/mL) for 30 min in the dark. PI incorporation without annexin binding indicated rapid cell death due to acute cytotoxicity of the compounds. The cells were analyzed using flow cytometry with an excitation wavelength of 488 nm and emission in the red fluorescence channel FL3. Doublets or cell aggregates were excluded from the analysis according to FL3-height versus FL3-area dot plot, as described earlier [17]. The cell cycle phase distribution was determined using the ModFit software (BD Dickinson).

### 4.8. Expression Level of Tumor Suppressor p53 Protein

Level of p53 (total, phosphorylated at Ser15 and Ser37, and acetylated at Lys382) was determined by the simultaneous measurement of various forms of p53 in the same probe. For this purpose, a set of specific antibodies conjugated with a fluorescent dye was used for intracellular staining according to the manufacturer’s procedures, and the relative fluorescence intensity was determined by flow cytometry using an LSRII BD cytometer. Changes in protein levels due to the effects of the compounds were analyzed in relation to the untreated control. The calibration of parameters such as autofluorescence, nonspecific isotype control binding, and equal PMT voltage allowed p53 expression level comparison between different cell lines. Percentage values (treatment *versus* control) were compared.

### 4.9. Reporter Gene Cell Lines and AP-1 and NF-κB Activity Assays

Commercially available adenocarcinoma cell lines were used for the assessment of the effect of the compounds on the activity of transcription factor AP-1 (Luciferase Reporter Stable HeLa cell line, 350-SL-0019-NP; Signosis, Inc., Santa Clara, CA, USA). A stable ovarian cancer reporter cell line NF-κB (SkoV3–Nf-κB) from the Regulation Laboratory IBM PAN, Poland, derived as previously described [68,69], was used for the assessment of NF-κB promoter activity. The cells were seeded into 96-well plates in the density of 1 × 10^4^ cell/well and maintained in culture medium for 24 h to allow attachment to the plate surface. After this time the cells were treated with cisplatin (2.5, 5, 10, or 15 μM) or compound **23** (5, 10, 20, or 40 μM) for 6 h and 24 h. For luciferase activity, the cells were washed in phosphate-buffered saline and resuspended in 50 μL of lysis buffer (25 mM Tris-phosphate, 4 mM EGTA, 1% Triton X-100, 15 mM MgSO_4_, and 2 mM dithiothreitol, pH 7.8) as described [70]. The plates were frozen in −70 °C for at least 20 min. After adjusting plates to room temperature, luciferase activity was measured in a EnVision 2103 Multilabel Reader (Perkin Elmer, Waltham, MA, USA) or in TECAN Infinite 200 PRO (TECANGroup Ltd. Mannedorf, Switzerland) using a solution of luciferase substrate (1.2 mM luciferin, 1.2 mM coenzyme A) in a kinetic mode.

### 4.10. Long-Term Cultures

#### 4.10.1. Long-Term Treatment with the Compounds

**Scheme 4:**
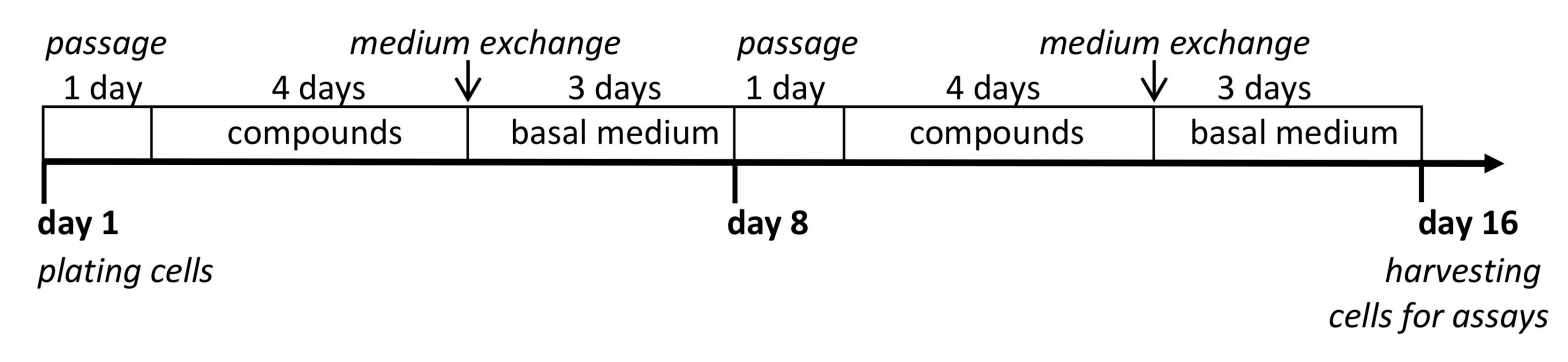
Long-time treatment of ovarian cancer cells with the compounds—design of experiment.

Cisplatin-resistant cells (A2780cis and A2780cisR) were cultivated with compound **23**, and references **2** and **25** to investigate the development of possible cross-resistance to common anticancer drugs. The cells were first cultivated in 5 mL of the culture medium [RPMI, 10% fetal bovine serum (FBS), and 1% P/S] containing the compounds (20 μM) for four days in 25 cm^2^ bottles. Subsequently, the medium was exchanged for the basal medium without the compounds and the cells were grown for an additional three days. Next, the cells were passaged by trypsinization, and the procedure was applied twice, ([4 + 3 + 1]_2_ regime, i.e., 4 days with and 3 days without a compound, plus 1 day of passage, repeated twice); the protocol is shown below (Scheme 4).

Medium exchange for a basal medium a few days prior to trypsinization was a routine procedure in the long-term treatment of cells with a given compound. The control cells were grown in the basal medium without the compounds, and alternatively, in the medium with the addition of the vehicle (DMF 0.02%), which did not affect cell growth. The experiment was repeated three times. The total number of cells was determined at each passage using counting microscope chamber. The cell growth rate and doubling time (h) were determined at the end of the culture period. The cell growth rate was calculated as the cell number over time between passages (seven days). The doubling time was calculated according to the following equation: T_d_ = 24 × (T_harvest_ − T_passage_) × log 2/(log N − log N_0_), where T_harvest_ − T_passage_ indicates the time between passages (7 days), and N_0_ and N denote the number of cells initially seeded and finally harvested, respectively.

#### 4.10.2. Cell Response to Cisplatin, Carboplatin, Paclitaxel, Doxorubicin, or Gemcitabine

Next, the cells were collected from different culture conditions and seeded into appropriate 24-well and 96-well plates (3 × 10^5^ and 2 × 10^4^ cells/well, respectively) for 24 h, followed by treatment with the following drugs: cisplatin, carboplatin, paclitaxel (PTX), doxorubicin (DOX), or gemcitabine (GEM) for 24 h and 48 h (at IC50 and half IC50). The cisplatin, carboplatin, DOX, PTX, and GEM concentration ranges were initially adjusted according to a cell line sensitivity evaluated using NR and MTT assays (0.001–100 µM, 0.01–200 µM, 0.0001–50 µM, 0.00001–10 µM, or 0.0001–10 µM, respectively). The sensitivity of the cells to the drugs was determined using the NR viability test, apoptosis/necrosis assay (PI and annexin V staining), and DCF-DA assay of ROS production, using flow cytometry.

### 4.11. Three-Dimensional Cultures, Spheroids

#### 4.11.1. Spheroid Formation

Spheroids were obtained from the A2780, A2780cis, A2780cisR, SKOV-3, and OVCAR-3 cell lines. Spheroids were cultured for up to three weeks, depending on the rate of spheroid formation (A2780 lines—7 days, SKOV-3—two weeks, OVCAR-3—3 weeks). The spheroid formation was followed by using light microscopy. Initially, 0.2 × 10^6^ cells per well suspended in the culture medium were seeded on a 24-well plate with an ultra-low attachment culture surface (Nunclon Sphera 24-well plates, Thermo Fisher Scientific, Waltham, MA, USA). The initial culture medium consisted of a 1–2-day culture medium that was appropriate for a given cell line, in a 1:1 ratio with a spheroid culture medium (DMEM: F12, 1: 1, supplemented with 2 g/L glucose, 5% FBS, penicillin, and streptomycin). During 1–3 weeks of culture (depending on the cell line), the culture medium was gradually changed 1:1 by gently discharging 1 mL of the old medium and supplementing it with 1 mL of the fresh medium for spheroids. The experiment was repeated three times using separate cell passages. For the experiments, the spheroid suspensions from 12 culture wells were pooled, the excess medium was removed, and 9 mL suspensions were distributed at 0.15 mL across the 96-well plate (Nunclon Sphera 96-well plates, Thermo Fisher Scientific, Waltham, MA, USA) and then treated with compounds, as described below.

#### 4.11.2. Treatment of Spheroids with Compounds

The spheroids in the suspension (0.15 mL) in the 96-well ultra-low attachment tissue culture plates were then treated with compound **23** alone, cisplatin, or in combination. In the combined treatment, the cells were incubated first with compound **23** (for 3 h, 40 μM) followed by 48- or 72-h culture with cisplatin (2.5, 5, 10, or 70 μM, depending on the experiment). In the control experiments, solvent (DMF) was added to the cell culture instead of the compound. The solvent did not affect cell viability at the concentrations used (≤0.06%). Preliminary results showed that OVCAR spheroids were extremely resistant to cisplatin. Therefore, the concentration of cisplatin was 50–70 μM in some experiments with OVCAR-3 spheroids. Five-to-six wells of the 96-well plate were used for one treatment of OVCAR-3 cells to increase the precision of the method and to prevent the effects of a possibly uneven distribution of spheroids of various dimensions in a given volume. In some experiments, compound **15** was used for comparison.

#### 4.11.3. Determination of Spheroid Viability

The viability of the cells was determined by using a CellTiter 96 3D Viability Assay Kit, according to the manufacturer’s procedure (Promega, Madison, WI, USA), based on the ATP luminescent measurement (RLU—relative luminescent units). The mean ± SEM values were calculated from experiments in triplicate. The effect of compound **23** alone (40 μM) and in combination with cisplatin on ATP levels was also measured in cells grown in a monolayer for comparison (A2780, A2780cis, A2780R, SKOV-3, and OVCAR-3).

#### 4.11.4. Microscopic Imaging of Spheroids

The spheroids were transferred to glass 8-well Nunc Lab-Tek II Chamber Slides (NUNC, Roskilde, Denmark), gently washed with warm HBSS/5% FBS, and incubated with CellTracker Green CMFDA (cytoplasm-selective fluorescent dye) at a concentration of 5 μM in HBSS for 30 min at 37 °C in a 5% CO_2_ atmosphere. At the same time, nuclei were stained with 5 μg/mL Hoechst 33258. After the procedures, the spheroids were gently washed with warm HBSS/5% FBS, and PI was added (2–5 μg/mL). The gentle washing of the spheroids after staining consisted of carefully removing the dye-containing buffer from the pellet of the spheroid, adding fresh buffer without dye, and repeating the procedure twice. The spheroids were processed without fixation (live-cell imaging). The concentration of dyes and the conditions of imaging (time and ambient temperature) did not affect cell viability. Spheroid staining was calibrated by selecting the lowest concentration of dyes for sufficiently intense emission and to prevent spectral overlap. Fluorescence photographs were collected by using the Nikon Eclipse TE 2000-U microscope (Nikon Plan Fluor 10×, 20× and 40× objectives, Nikon, Japan) equipped with UV excitation UV-1A, blue excitation bandpass B-2E/C, and green excitation G-2A filters and a DS-U2 digital color camera (Nikon), using equal exposure times and gain parameters. Differential interference contrast (DIC, Nomarski interference contrast) images were collected to visualize the spheroid morphology. The camera was remotely controlled using NIS Elements AR Analysis 3.2 software. The resolution of the acquired digital images was 2560 × 1920 pixels. The original images were archived in nd2 format and converted to jpg. The method of imaging large 3D structures (focusing on individual surfaces of the sphere) is described in the Supplementary Materials (Figure S11). In addition, time-lapse imaging along the *z*-axis was used to study the morphology of the largest OVCAR-3 spheroids (video and frame-by-frame analysis).

### 4.12. Statistical Analysis

The Statistica software package, version 13.3 (TIBCO Software Inc., Palo Alto, CA, USA), was used for the calculations. Parametric or nonparametric multiple comparison tests were performed according to data distribution. The type of data distribution was determined using the Shapiro–Wilk test. Data have been expressed in terms of mean ± SEM of separate independent experiments. Homogeneity of variance was verified using Levene’s test. Data were analyzed by means of parametric or nonparametric tests of multiple comparisons, in accordance with the repeat number and distribution. Differences between treatment groups and controls were assessed by analysis of variance (ANOVA) or the Kruskal–Wallis test, followed by two-tailed Student’s *t*-test or nonparametric tests, respectively with the Bonferroni correction for post-hoc evaluation, unless mentioned otherwise. The strengths of associations and correlations were evaluated by the Spearman nonparametric test. *p* values were two-sided; *p* ≤ 0.05 was considered statistically significant. The relative similarities of the compounds according to their effects on cell viability were studied using hierarchical cluster analysis (unweighted pair grouping using mathematical averages method, UPGMA). The hierarchical and k-means clustering methods were used to classify the compounds in relation to their anticancer activity into four groups: active (toxic), moderately active, low active, and non-active; the cell lines were classified into four groups according to their platinum susceptibility: sensitive, moderately resistant, resistant, and highly resistant. Discriminant analysis was performed to verify grouping predictability and relationships between qualitative descriptors of the compounds and their biological activities. The experimental values of cell response to the compounds were referred to as control values and gathered for the common numerical analysis. Data standardization was performed according to the following formula: cell_response = (cells_treated_ − cells_control_)/cells_control_, where *cells_treated_* indicates values obtained for cells treated with compounds, and *cells_control_* denotes values for control cells not treated with compounds. Dose-response curve fitting for IC50 calculations was done by using GraphPad Prism 6.01 software (San Diego, CA, USA) and AAT Bioquest server tools (Sunnyvale, CA, USA).

### 4.13. Physicochemical Characterization of Compounds

Nuclear magnetic resonance (NMR): The ^1^H, ^13^C, dept135 and ^11^B-NMR spectra in CDCl_3,_ CD_3_OD or CD_3_COCD_3_ were recorded using a Bruker AVANCE III HD (Billerica, MA, USA) 500 MHz, a BrukerAvance DPX 250 MHz, or Varian Mercury 400 MHz spectrometer, resp.; shift values in parts per million are relative to the SiMe_4_ internal reference. Multiplets were assigned as s (singlet), bs (broad singlet), d (doublet), t (triplet), m (multiplet), bm (broad multiplet), dd (doublet of doublet), dt (doublet of triplet), td (triplet of doublet), tt (triplet of triplet), and ddd (doublet of doublet of doublet). Coupling constants are reported in Hertz (Hz).

Mass spectrometry. Fast atom bombardment (FAB) mass spectra were recorded on a Finnigan MAT (Bremen, Germany). The *m*/*z* was measured in a positive and negative modes. The MALDI-TOF spectra were recorded on a Voyager Elite mass spectrometer (PerSeptive Biosystems Inc., Framingham, MA) in the linear, negative ion mode. The ESI spectra was recorded on a Waters HPLC/MS (Milford, MA, USA). High-resolution mass spectra were recorded on a LTQ Orbitrap Velos instrument, Thermo Scientific (Waltham, MA, USA). The calculated m/z corresponds to calculated values based on the average mass of the elements consisting natural isotopes.

Ultraviolet spectroscopy measurements (UV). UV measurements were performed on a GBC Cintra10 UV-VIS spectrometer (Dandenong, Australia). Samples for UV experiments, ca. 0.5 A_260_ OD of each compound were dissolved in 96% C_2_H_5_OH. The measurement was performed at ambient temperature.

Attenuated total reflectance−Fourier transform infrared (ATR−FTIR) spectroscopy measurements: infrared absorption spectra were recorded using a Smart iTR diamond attenuated total reflectance (ATR) attachment on a Nicolet 6700 FT-IR spectrometer (Thermo Scientific) equipped with an ETC EverGlo* source for the IR range, a Ge-on-KBr beam splitter, and a DLaTGS detector. Samples to be analyzed were placed on a diamond ATR element in the solid form. The Omnic 8.1 software program was used for data acquisition and processing. Alternatively, a Jasco 6200 (Easton, MD, USA) FT-IR spectrometer was used and spectra run in KBr pellet.

### 4.14. X-ray Crystallography

X-ray structure analysis of 8-dioxane-[3-chromma bis(1,2-dicarbollide)] (**7**): X-ray diffraction analysis of the cesium salt of the parent chromium sandwich Cs **3** and the zwitterion **7** (dark violet-red crystals obtained by overlaying CH_2_Cl_2_ or benzene solutions with hexane, respectively, and slow crystallization) was performed with a Rigaku XtaLAB Synergy S diffractometer equipped with Mo (Mo/*K_α_* radiation; *λ* = 0.71073 Å) and Cu (Cu/*K_α_* radiation; *λ* = 1.54184 Å) micro-focus X-ray source and Hybrid Pixel Array Detector (HyPix-6000HE). An Oxford Cryosystems (Cryostream 800) cooling device was used for data collection at a temperature of 100 K. CrysAlis Pro software was used for data collection and cell refinement, data reduction, and absorption correction (CrysAlisPRO, Version 1.0.43, Oxford Diffraction/Agilent Technologies UK Ltd., Yarnton, UK, 2020). Data were corrected for absorption effects using empirical absorption correction (spherical harmonics), implemented in the SCALE3 ABSPACK scaling algorithm and numerical absorption correction based on Gaussian integration over a multifaceted crystal model.

The structures of prepared compounds 3 and 7 were solved with the ShelXT [71] structure solution program using Intrinsic Phasing and refined with the ShelXL refinement package [72] using least squares minimization implemented in Olex2 [73]. Anisotropic displacement parameters were refined for all non-H atoms. All hydrogen atoms were localized on a difference Fourier map. For crystallographic data and structure refinement see ESI. Molecular graphics for 3 and 7 were generated using DIAMOND software (Diamond, v4.6.3,) [74]. For crystal data and structure refinement, see Table S1 in the Supplementary Materials. Crystallographic data for structural analysis have been deposited with the Cambridge Crystallographic Data Center, CCDC nos. 2042928 and 2042960. Copies of this information may be obtained free of charge from The Director, CCDC, 12 Union Road, Cambridge CB2 1EY, UK (fax: +44-1223-336033; e-mail: deposit@ccdc.cam.ac.uk or www: http://www.ccdc.cam.ac.uk, accessed on 21 January 2021).

### 4.15. Synthesis of Compounds

Synthesis of 6-*N*-{5-[3,3′-cobalta-bis(1,2-dicarba-*closo*-undekaborate)-8-yl]-3-oxa-pentoxy}-2′-deoxyadenosine (**14**) [34], 6-*N*-{5-[3,3′-ferra-bis(1,2-dicarba-*closo*-undekaborate)-8-yl]-3-oxa-pentoxy}-2′-deoxyadenosine (**15**) [36] and 6-*N*-{5-[chroma-bis(1,2-dicarba-*closo*-undekaborate)-8-yl]-3-oxa-pentoxy}-2′-deoxyadenosine (**16**) [35] was performed according to the literature procedure.

Synthesis of 8-ethynyl-2′-deoxyadenosine (**17**) was performed according to the literature procedure [54].

Synthesis of 2′-*O*-(1-pentyn-5-yl)adenosine (**21**) was performed as described [55].

Synthesis of 2′-*O*-{[5-(7,8-dicarba-*nido*-undekaborate-10-yl)-3-oxa-pentoxy]-1*N*-1,2,3-triazol-4-yl}propyloadenosine (**25**) was performed as described [32].

Synthesis of 8-dioxane-[3, 3′-chromma bis(1,2-dicarbollide)] (**7**).

[3-Chromma-bis(1,2-dicarba-*closo*-undecaborate](−1) (**3**) (1.71 g, 3.8 mmol) was suspended and freshly dried with sodium and distilled dioxane (5.5 mL) under stirring. Then, BF_3_.Et_2_O (1.62 g, 11.4 mmol) was added via syringe through a septum and the reaction mixture was heated to reflux temperature under continuous stirring and refluxed for 4 h. The resultant slurry was left to cool down, then an excess of dioxane was evaporated under reduced pressure. To the resultant violet *semi*-solid residue, a 30% aqueous ethanol (5 mL) was added, then the whole was instantly filtered, and the solids on the Schott sintered filter funnel were washed quickly twice with water. The resultant crude product was dried in vacuum for 30 min. To obtain the purified preparation, the crude product was dissolved in benzene (20 mL), the resultant solution was dried over solid MgSO_4_, filtered, then the filtrate was partly evaporated to a volume ca. 10 mL. The two volume portions of hexane were carefully layered, then the whole was left to stand to crystallize for two days. The crystalline product was recovered by filtration (or eventually by decantation) and for further synthesis was dried in vacuum at 60 °C for 8 h.

Compound **7**: Dark violet solid, yield 0.803 g, 52%; TLC (CH_3_CN/CH_2_Cl_2_ 1:3): R_f_ = 0.72; HPLC (Column: Separon SGX C8 250x 4 mm I.D., 3 mM hexylamine acetate pH 6.5 in 65% aqueous CH_3_CN, 1 mL/min, detection DAD UV 237 and 398 nm) k’ = 7.60, purity 98%, UV/Vis (CH_3_CN): λ_min_ = 223.1, 265.0 nm, λ_max_ = 235.9, 297.5 nm; ^1^H NMR (400 MHz, CD_3_COCD_3_, 25 °C, TMS): δ = 4.22 (4H, s, CH^carborane^), 1.35 (4H, m, dioxane), 0.92 (4H, m, dioxane), ^13^C{1H} NMR (100.58 MHz, CD_3_COCD_3_, 25 °C, TMS): δ = 132.35 (2C, dioxane), 122.79 (2C, dioxane), 77.40 (2C, CH^carborane^), 69.35 (2C, CH^carborane^); ^11^B NMR (128.33 MHz, CD_3_COCD_3_, 25 °C, BF_3_/(C_2_H_5_)_2_O): δ = 225.21 (1B, s), 111.62 (1B,s), 84.55 (1B, s), −42.55 (2B, s), −77.55 (2B, s), −175.80 (1B, s), signals from boron atoms (B8, B8′, B4,7, B4′7′) adjacent to Cr(III) could not be found in the range 2000 ppm to −4000 ppm, apparently due to their very extensive broadening due to interaction with paramagnetic high spin center. MS (ESI- in CH_3_OH), C_8_H_32_B_18_O_3_Co *m*/*z* (%): found 435.50 (100%), 438.24(10%) [M + CH_3_O]^−^, calculated 435.36 (100%), 438.35(15%).

Synthesis of 8-(5-azido-3-oxa-pentoxy)-3,3′-chroma bis(1,2-dicarba-*closo*-undekaborate](-1) (**11**).

Anhydrous 8-dioxane-3-chromium bis(dicarbollide) (122 mg, 0.22 mmol) and sodium azide (16.83 mg, 0.25 mmol) were dissolved in dry dimethylformamide (DMF, 2.4 mL). The mixture was stirred at ambient temperature under an inert atmosphere of dry argon (60 min) and the reaction progress was monitored by TLC (CH_2_Cl_2_/CH_3_OH 9:1) until complete conversion of the starting material. Then, the solvent was evaporated to dryness under vacuum. A crude product was purified by silica gel column chromatography (230–400 mesh) using a linear gradient of methanol in methylene chloride (from 0% to 20%) as an eluent, yielding 100 mg of the product (**11**). Yield: 82%.

Synthesis of 8-{5-[3,3′-cobalta-bis(1,2-dicarba-*closo*-undekaborate)-8-yl]-3-oxa-pentoxy}-1*N*-1,2,3-triazol-4-yl}-2′-deoxyadenosine (**18**), 8-{5-[3,3′-ferra-bis(1,2-dicarba-*closo*-undekaborate)-8-yl]-3-oxa-pentoxy}-1*N*-1,2,3-triazol-4-yl}-2′-deoxyadenosine (**19**) and 8-{5-[3-chroma-bis(1,2-dicarba-*closo*-undekaborate)-8-yl]-3-oxa-pentoxy}-1*N*-1,2,3-triazol-4-yl}-2′-deoxyadenosine (**20**).

8-Ethynyl-2′-deoxyadenosine (**17**) (58.6 mg, 0.21 mmol) and 8-(5-azido-3-oxapenthoxy)-3-metala-bis(1,2-dicarba-*closo*-undecaborate) (100 mg, 0.22 mmol) were dissolved in a mixture of *tert*-butanol and water 2.5 mL (1:1, *v*/*v*). Next, a freshly prepared solution of CuSO_4_·5H_2_O (100 mM) and potassium ascorbate (100 mM) was added simultaneously. The reaction mixture was stirred at room temperature for 24 h and the reaction progress was monitored by TLC CH_2_Cl_2_/MeOH (9:1). Then the solvent was removed in vacuo and the crude material was purified by column chromatography using a gradient of methanol in dichloromethane.

Yield **18**: 76%. TLC (CH_2_Cl_2_/MeOH, 8.4:1.6): *R_f_* 0.24, UV (96% EtOH): _max_ = 304.92 nm, FT-IR: ν_max_(KBr/cm^−1^) = 3439 (OH), 2927, 2872 (CH_2_), 2560 (BH), ^1^HNMR (MeOD, 250.131MHz, 25 °C, TMS), (δ, ppm): 8.62 (s, 1H, H-2), 8.15 (s, 1H, H^triazole^), 7.24 (dd, 1H, H-1′, *^3^J_HH_* = 9.03, 8.92, 5.97Hz), 4.75-4.60 (m, 2H, CH_2_N), 4.13–4.0 (m, 5H, H-3′, 4 × CH^metallacarborane^), 3.99–3.55 (m, 9H, H-4′, H-5′, 3 × CH_2_), 3.15–2.95 (m, 2H, H-2′), MS (Gly, FAB, -Ve) *m*/*z* (%): 731.3 (20%) [M], (C_20_H_42_B_18_CoN_8_O_5_ calculated 731.43).

Yield: **19** 92%. TLC (CH_2_Cl_2_/MeOH, 8.4:1.6): *R_f_* 0.23, UV (96% EtOH): λ_max_ = 283.34 nm, FT-IR: ν_max_(KBr/cm^−1^) = 3439, 3385 (OH), 2961, 2927, 2872 (CH_2_), 2557 (BH), MS (Gly, FAB, -Ve) *m*/*z* (%): 728.5 (20%) [M], (C_20_H_42_B_18_FeN_8_O_5_ calculated 728.43).

Yield **20**: 15%. UV–Vis (95% C_2_H_5_OH): λmin = 212 nm, 254 nm, λmax = 228 nm, 297 nm; FT-IR: ν_O-H_ = 3141, 3195, 3334, ν_CH2_ = 2927, 2871, ν_B-H_ = 2533; ^1^H NMR (600 MHz, (CD_3_)_2_O) δ (ppm): 0.86–2.82 (m, 18H, BH-metallacarborane), 3.09–3.18 (m, 2H, H-2′), 3.70–3.89 (m, 9H, H-4′, H-5′, 3 × CH_2_), 4.01–4.70 (m, 5H, H-3′, 4xCH-metallacarborane), 4.89–5.00 (m, 2H, CH_2_N), 6.70–6.90 (m, 1H, H-1′), 7.53 (s, 1H, H-triazole), 8.19 (s, 1H, H-2), ^13^C NMR (152.90 MHz, MeOD) δ (ppm): δ 157.57 (C6), 153.04 (C2), 151.22 (C4), 143.91 (C8), 140.20 (C4″-triazole), 131.41 (C5″-triazole), 121.15 (C5), 90.43 (C4′), 88.88 (C1′), 74.69 (CH_2_- linker), 73.79 (C3′), 64.51 (C5′), 57.62 (CH_2_-linker), 53.87 (CH_2_-linker), 50.00 (CH_2_-linker), 41.72 (C2′); TLC CH_2_Cl_2_/MeOH (9:1 *v*/*v*) Rf = 0.2; MS (MALDI, -VE): *m*/*z* = 721.3 [M], calcd for (C_20_H_42_B_18_CrN_8_O_5_) = 721.19).

2′-*O*-{{5-[3,3′-cobalta-bis(1,2-dicarba-*closo*-undekaborate)-8-yl]-3-oxa-pentoxy}-1*N*-1,2,3-triazol-4-yl}propyloadenosine (**22**). Yield: 68%; TLC (CH_2_Cl_2_:MeOH, 8:2 *v*/*v*) R*_f_* = 0.15; UV-Vis (MeOH): λ_max_ = 262.2, 312.12 nm; ^1^H NMR (250.13 MHz, MeOD, 25 °C, TMS): δ = 8.30 (s, 1H, H-2), 8.20 (s, 1H, H-8), 7.85 (s, 1H, H^triazole^), 6.06 (d, 1H, H-1′, ^3^*J_HH_* = 6.0Hz), 4.51–4.47 (m, 5H, H-2′, NCH_2_CH_2_−), 4.28–4.25 (m, 3H, H-3′, OCH_2_CH_2_CH_2_) 4.10–4.20 (m, 1H, H-4′), 4.09–4.00 (bs, 4H, 4 × CH^carborane^), 3.90-3.70 (m, 4H, H-5′, CH_2_), 3.65–3.55 (m, 2H, OCH_2_CH_2_O), 3.50–3.40 (m, 2H, OCH_2_CH_2_O); 1.85–1.66 (m, 2H, CH_2_CH_2_CH_2_), 1.61–1.72 (m, 2H, CH_2_CH_2_CH_2_); 0.5–3.4 (bm, 17H, BH); ^11^B NMR (80.250 MHz, MeOD), ^11^B {^1^H BB}: δ = 24.86 (s, B8), 6.56 (s, B8′), 2.04 (s, B10), −1.10 (s, B10), −3.37 (s, B4′,7′), −6.28 (bs, B9′,12′,9,12,4,7), −16.19 (s, B5′11′), −19.55 (bs, B5,11,6′), −28.16 (s, B6); FT-IR (KBr cm^−1^): ν_max_ = 3424 (OH), 2958, 2929, 2872 (CH_2_), 2562 (BH); MS (FAB, Gly, -Ve): *m*/*z* = 789.30 [M]^+^ (C_23_H_48_B_18_CoN_8_O_6_, calculated: 789.47, 100%).

Synthesis of 2′-*O*-{{5-[3, 3′-ferra-bis(1,2-dicarba-*closo*-undekaborate)-8-yl]-3-oxa-pentoxy}-1*N*-1,2,3-triazol-4-yl}propyloadenosine (**23**) and 2′-*O*-{{5-[3-chromma-bis(1,2-dicarba-*closo*-undekaborate)-8-yl]-3-oxa-pentoxy}-1N-1,2,3-triazol-4-yl}propyloadenosine (**24**).

The substrates 2′-*O*-penthynyladenosine (**21**) (297 mg, 0.891 mmol) and 8-(5-azido-3-oxapenthoxy)-3-iron-bis(1,2-dicarba-*closo*-undecaborano)an (**10**) (492 mg, 1 mmol) or 2′-*O*-penthynyladenosine (**21**) (66.5 mg, 0.1997 mmol) and 8-(5-azido-3-oxapenthoxy)-3-chromium-bis(1,2-dicarba-*closo*-undecaborano)an (**11**) (100 mg, 0.22 mmol) were dissolved in a mixture of tert-BuOH/H_2_O (1:1, 2.5 mL). Solutions of CuSO_4_ × 5H_2_O (100 mM) and sodium ascorbate (100 mM) were added to the reaction mixture. The reaction was stirred in room temperature for 24 h, and monitored by TLC CH_2_Cl_2_/MeOH (9:1). Then, the solvent was removed in vacuum and crude material was purified by column chromatography using a gradient of methanol in dichloromethane. After reaction completion (usually 24 h), the solvent was evaporated under vacuum. The crude product was purified by silica gel column chromatography using a gradient of methanol in methylene chloride as an eluent. Pure compounds were lyophilized from a mixture of *tert*-butanol and water (1:1, *v*/*v*) or benzene/methanol (9:1, *v*/*v*).

Compound **23**: Yield: 30%. FT-IR: ν_N-H_ = 3334, ν_O-H_ = 3196, 3141, ν_CH-metallacarborane_ = 3039, ν_CH_ = 2927, 2871, ν_B-H_ = 2583, ν_C=N_ = 1639, ν_C-OH_ = 1092; ^1^H NMR (600 MHz, MeOD) δ (ppm): 0.40–2.30 (m, 20H, BH-metallacarborane), 3.33–3.37 (m, 4H, overlapped by methanol signal, 2 × CH_2_), 3.43 (d, 1H, 4′-H, J = 12.0 Hz), 3.55–3.61 (m, 2H, 2 × CH_2_), 3.62–3.69 (m, 2H, H-2′,H- 3′), 3.70–3.81 (m, 2H, C-CH_2_-O), 4.60 (bs, 1H, triazole-CH_2_-C), 5.29 (d, 1H, d, H-1′, J = 6.0 Hz), 7.76 (s, 1H, s, H-8), 7.88 (s, 1H, H-2); ^13^C NMR (152.90 MHz, MeOD) δ (ppm): 18.70 (C), 27.03 (C-metallacarborane), 45.20 (CH_2_-C-triazole), 60.74 (C5′), 61.56 (CH_2_), 67.61 (CH_2_), 68.88 (C3′), 80.87 (C2′), 86.47 (C1′), 87.34 (C4′), 118.59 (C5), 139.99 (C-triazole), 143.73 (H8), 147.96 (C4), 151.71 (C2), 155.54 (C6); TLC CH_2_Cl_2_/MeOH (9:1 *v*/*v*) Rf= 0.33; MS (MALDI, -VE): *m*/*z* = 783.5 [M], calcd for (C_23_H_48_B_18_FeN_8_O_6_) = 783.12).

Compound **24**: Yield: 15%. UV–Vis (95% C_2_H_5_OH): λmin = 233 nm, 293 nm, λmax = 262 nm, 312 nm; FT-IR: ν_O-H_ = 3136, 3022, ν_CH2_ = 2928, 2870, ν_B-H_ = 2528; ^1^H NMR (600 MHz, (CD_3_)_2_O) δ (ppm): 0.5-3.0 (m, 18H, BH-metallacarborane), 3.60-3.91 (m, 5H, OCH_2_), 4.22-4.61 (m, 7H, H-3′,H-4′, H-5′, 4 × CH-metallacarborane), 6.10 (s, 1H, H-1′), 7.40 (bs, 1H, H-triazole), 8.21 (s, 1H, H-2), 8.36 (s,1H, H-8); ^13^C NMR (152,90 MHz, MeOD) δ (ppm): 41.72 (C2′), 50.00 (CH_2_-linker), 53.87 (CH_2_-linker), 57.62 (CH_2_-linker), 64.51 (C5′), 73.79 (C3′), 74.69 (CH_2_-linker), 88.88 (C1′), 90.43 (C4′), 121.15 (C5), 131.41 (C5″-triazole), 140.20 (C4″-triazole), 143.91 (C8), 151.22 (C4), 153.04 (C2), 157.57 (C6); TLC CH_2_Cl_2_/MeOH (9:1 *v*/*v*) Rf = 0.2; MS (MALDI, -VE): *m*/*z* = 779.2 [M], calcd for (C_23_H_48_B_18_CrN_8_O_6_ = 779.27).

## 5. Conclusions

The results obtained here show that adenine nucleosides modified with metalla bis(dicarbollides) may aid in sensitizing ovarian cancer cells to chemotherapeutic agents such as cisplatin in combination therapy. Compound **23** [a conjugate of adenosine and ferra bis(dicarbollide) ion] has been identified as an anticancer lead compound, which shows high intracellular accumulation, toxicity against both cisplatin-sensitive and cisplatin-resistant cancer cells, cytotoxic activity against cancer spheroids, and enhanced cisplatin proapoptotic effects. Moreover, in cancer cells, it does not induce cross-resistance to five standard anticancer drugs—cisplatin, carboplatin, doxorubicin, paclitaxel, and gemcitabine—in long-term cultures. It is also worth noting that only the combination of a nucleoside with a metallacarborane protects cells from developing multidrug cross-resistance while exerting proapoptotic effects.

The results obtained using ovarian cancer spheroids indicated that appropriately designed adenosine derivatives, utilizing the unique properties of metalla bis(dicarbollide) ion modification, may help to design drugs that resensitize cancer cells to chemotherapeutic agents in combination therapy. The use of adenosine derivatives modified with boron clusters to overcome drug resistance may be important in the context of cisplatin-resistant cells, which are sensitive to these compounds as parental cell lines. The switching-on of death signaling mechanisms in cancer cells by metallacarborane derivatives can also yield therapeutic benefits in the future.

## Figures and Tables

**Table 1:** Comparison of platinum sensitivity of six ovarian carcinoma cell lines (IC_50_, mean ± SEM).

Similarity * %	Cel Line	Cisplatin (μM)	*p* Value	Resistance Index **	Carboplatin (μM)	*p* Value	Resistance Index *
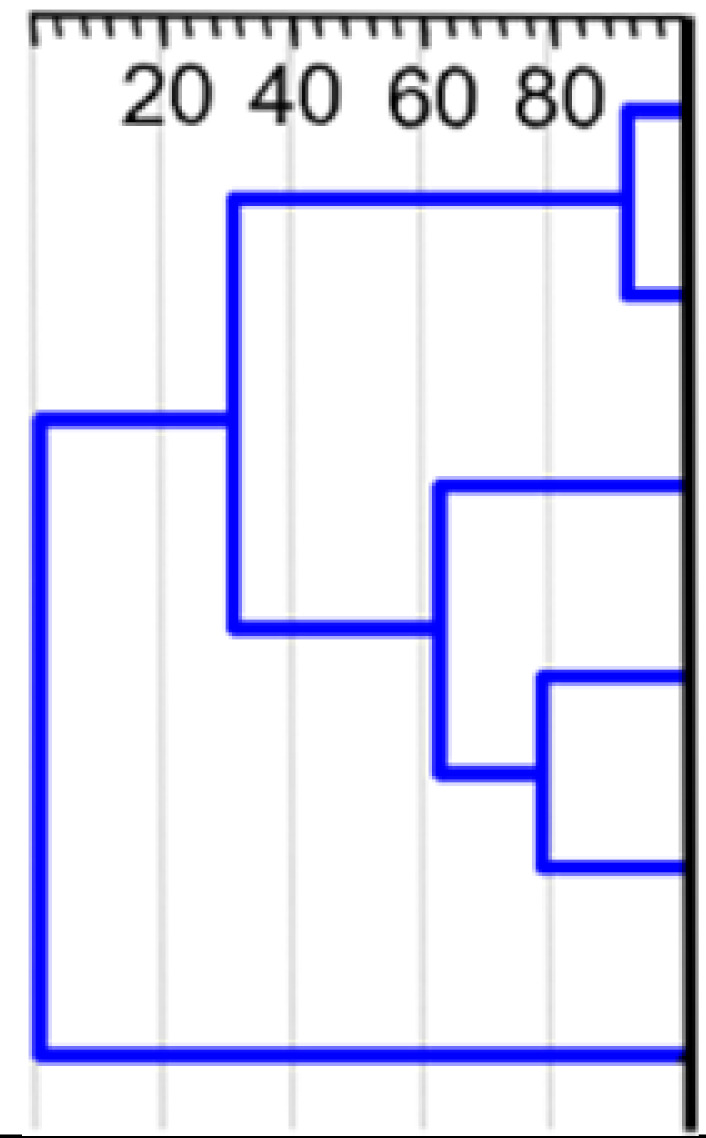	A2780	2.8 ± 0.21	—	—	51 ± 4.4	—	—
	OVCAR-3	5.1 ± 0.45	0.971	n.a.	55 ± 4.4	0.945	n.a.
	A2780cis	15 ± 0.64	<0.001	5.4	108 ± 4.9	<0.001	2.1
	SKOV-3	21 ± 1.6	<0.001	n.a.	211 ± 11	<0.001	n.a.
	A2780cisR	24 ± 0.89	<0.001	8.6	162 ± 5.6	<0.001	3.2
	A2780cisKB	37 ± 2.1	<0.001	13.2	230 ± 10	<0.001	4.5

N.a.—not applicable; similarity*—grouping cell lines according to platinum susceptibility using unweighted pair-group method with arithmetic mean (UPGMA), Euclidean distance; ** The resistance index was calculated as the ratio between the IC50 of resistant and sensitive cells; the mean IC50 ± SEM values were calculated for n ≥ 6 independent experiments; *p* values calculated for comparisons with IC50_A2780_, post-hoc Tukey’s test following ANOVA; data distribution and homogeneity of variance were verified using the Shapiro–Wilk and Levene’s tests. Relevant comparisons; cisplatin: IC50_A2780cis_ vs. IC50_A2780cisR_, *p* < 0. 0001; IC50_A2780cis_ vs. IC50_A2780cisKB_, *p* < 0.0001; IC50_A2780cisR_ vs. IC50_A2780cisKB_, *p* < 0.0001; IC50_OVCAR-3_ vs. IC50_A2780cis_, *p* < 0.001; IC50_SKOV-3_ vs. IC50_A2780cis_, *p* = 0.0525; IC50_SKOV-3_ vs. IC50_A2780cisR_, *p* = 0.490; IC50_SKOV-3_ vs. IC50_A2780cisKB_, *p* < 0.0001; carboplatin: IC50_A2780cis_ vs. IC50_A2780cisR_, *p* < 0.0001; IC50_A2780cis_ vs. IC50_A2780cisKB_, *p* < 0.0001; IC50_A2780cisR_ vs. IC50_A2780cisKB_; *p* < 0.0001; IC50_OVCAR-3_ vs. IC50_A2780cis_, *p* < 0.0001; IC50_SKOV-3_ vs. IC50_A2780cis_, *p* < 0.0001; IC50_SKOV-3_ vs. IC50_A2780cisR_, *p* < 0.01; IC50_SKOV-3_ vs. IC50_A2780cisKB_, *p* = 0.990; vs.—*versus*. Hierarchical and k-means clustering methods were used to classify the cell lines according to their susceptibility to platinum drugs (IC50 values). The results were verified using discriminant analysis, which showed a 98% accuracy of the classification. Four groups were differentiated: sensitive (A2780 and OVCAR-3) > moderately resistant (A2780cis) > resistant (A2780cisR and SKOV-3) > highly resistant (A2780cisKB).

**Table 2:** Cytotoxicity of compound **23** in different ovarian carcinoma cell lines depending on their cisplatin (10 μM) sensitivity and form of growth—monolayer or spheroids. Measurements of cellular ATP level.

Cell Line	Cisplatin		Compound 23		Spheroid Structure
	Adherent	Spheroids	Adherent	Spheroids	
OVCAR-3	40	100	49	36	compact
A2780	44	49	52	81	loose
A2780cis	82	88	58	91	loose
A2780cisR	89	89	49	83	loose
SKOV-3	82	81	24	89	loose

Cell viability was determined by using the CellTiter-Glo 3D Viability Assay (cisplatin 10 μM, **23** 40 μM). Average percentage values of cell viability are shown (untreated cells, 100%). Color intensity corresponds to compound cytotoxicity. Color scale: from nontoxic (white) to the most toxic (red). OVCAR-3 cells grown in compact spheroids became refractory to cisplatin, but also became more sensitive to compound **23**. The cells in loose spheroids retained their sensitivity to cisplatin, but became resistant to compound **23.**

## Data Availability

Data are contained within the article or Supplementary Materials.

## Supplementary Materials

The following are available online at https://www.mdpi.com/article/10.3390/cancers13153855/s1, Figure S1: Effect of compounds **1**–**24** (40 μM) on the necrosis and late apoptosis of A2780cis cells, Figure S2: Effect of compounds **23**, **2**, and cisplatin on apoptosis and necrosis in A2780 sublines, A2780, A2780cis, and A2780cisR, Figure S3: Effect of increasing doses of cisplatin on plasma membrane potential in A2780, A2780cis, and A2780cisR cells (A); (B) the reference experiment demonstrating decrease in plasma membrane potential in A2780cis cells in response to toxic effect of sodium azide, Figure S4: Relationships of incorporation of metallacarboranes unmodified or adenosine derivatives into A2780cis cells to cell viability (non-apoptotic cells, %) and necrosis (PI, %), Figure S5: Expression levels of tumor suppressor protein p53 and its phosphorylated and acetylated forms in ovarian cancer cell lines A2780, A2780cis, A2780cisR, SKOV-3, and OVCAR-3; basal expression level and after treatment with cisplatin, Figure S6: Dose-dependent increase in expression of p53 (upper panel) and p53 p53(pS15) (lower panel) in ovarian cancer cells, stimulated with compound **23**, in comparison to treatment with compound **2**, Figure S7: Effect of compounds **23** and **2** on the expression levels of p53 and its phosphorylated forms (p53 pS15 and p53 pS37) in various cell lines of ovarian cancer, Figure S8: Apoptosis (A) and necrosis (B) of A2780cisR cells cultured for long-term in medium with addition of compounds **2**, **23**, or **25** exposed to cisplatin (cis-Pt), carboplatin, DOX, PTX, or GEM, Figure S9: Viability of cells cultured in different conditions (i.e., with compounds **2**, **23**, or **25**, or without compounds (control and control/DMF)), and next treated for 48 h with DOX (0.16 μM), PTX and GEM (0.08 μM); data for neutral red (NR) assay were expressed as percentage values of control cells not treated with drugs (100%), Figure S10: ROS production in A2780cisR cells cultured for long-term with compounds **23**, **2**, or **25**, Figure S11: Differential interference contrast (DIC) image of the spheroids formed by OVCAR-3 cells focused on the following areas of the spheroid, along the z–axis: boundary, slope and top, Figure S12: The effect of solvent (DMF) on the viability of spheroids and adherent cells cultured for 48 h (37 °C and 5% CO2 in humidified atmosphere) at concentrations equal to DMF content in the samples incubated with the compounds (**23** and cisplatin alone, or in combination), Figure S13: Effect of compounds **15** and **23** alone and in combination with cisplatin on the viability of OVCAR-3 spheroids, Figure S14: Crystal packing in compound **3** along the *ab* plane, Figure S15: The Cs^+^ cations surrounded by [3,3′-Cr(1,2-C_2_B_9_H_11_)_2_]^–^ anions located in the cavities of **3**, Figure S16: Crystal packing in compound **7** along the *ab* plane, Table S1: Effect of compounds **23** and **2** on p53 expression expressed as fold change (treated/control), Table S2: Crystal data and structure refinement for compounds **3** and **7**.
