# Supplementary material for: Interaction of Adenosine, Modified Using Carborane Clusters, with Ovarian Cancer Cells: A New Anticancer Approach against Chemoresistance
Source: Cancers (Basel). 2021 Jul 30;13(15):3855. doi: 10.3390/cancers13153855 (PMC8345486; doi:10.3390/cancers13153855)
Supplement: Supplementary file 1 [file cancers-13-03855-s001.zip › cancers-1308492-supplementary.pdf]

Supplementary Materials

Katarzyna Bednarska-Szczepaniak,[1,*] Ewelina Przelazły,[1] Katarzyna Kania,[2,3] Marzena Szwed,[4] Miroslava Litecká,[5] Bohumír Grűner,[5] Zbigniew J. Leśnikowski[1]

[1] Institute of Medical Biology, Polish Academy of Sciences, Laboratory of Medicinal Chemistry, 106 Lodowa, 92-232 Lodz, Poland; kbednarska@cbm.pan.pl (K.B-S.); zlesnikowski@cbm.pan.pl (Z.J.L); ewelina.przelazly@gmail.com (E.P)

[2] Institute of Medical Biology, Polish Academy of Sciences, Laboratory of Transcriptional Regulation, 106 Lodowa, 92-232 Lodz, Poland; kkania@cbm.pan.pl

[3] Institute of Medical Biology, Polish Academy of Sciences, Laboratory of Virology, 106 Lodowa, 92-232 Lodz, Poland; kkania@cbm.pan.pl

[4] University of Lodz, Institute of Biophysics, Department of Medical Biophysics, Pomorska 141/143, 90-236 Lodz, Poland; marzena.szwed@biol.uni.lodz.pl

[5] Institute of Inorganic Chemistry of the Czech Academy of Sciences, Hlavní 1001, 250 68 Rež, Czech Republic; gruner@iic.cas.cz (B.G.); litecka@iic.cas.cz (M.L.)

* Correspondence: kbednarska@cbm.pan.pl

I. Biology

Methodology details – UPGMA analysis

To examine structure-activity relationship, the characteristics of the compounds were finally defined: a) structural: presence of a nucleoside, type of metal ion, and modification site; b) functional: effect on cell viability and accumulation in cells. The effect on cell viability was expressed as i) variables as raw data (IC50 values), ii) categorical variables (e.g., active and non-active). The creation of similarity groups according to the activity of the compounds, compared with their chemical structure (type of metal ion, substitution site, and presence of the nucleoside unit), allowed the formulation of more general conclusions. Detailed analysis showed that the three unmodified metalla bis(dicarbollide) ions were clustered distinctly separately from the modified adenosine derivatives; compound **3** was found to be nontoxic and compounds **1** and **2** formed a pair of slight or moderate inhibitors of cell viability. The C2′ and C8 adenosine derivatives were clustered together as two groups with high percentage similarity in toxicity (80%); compounds **22**, **23**, and **24** were close (90% similarity) and separated slightly from compounds **18**, **19**, and **20**. Among the adenosine derivatives, compound **14** was found to be the least toxic in this group. Compound **15** was highly active against OVCAR-3 and SKOV-3 cells, with more than twice the toxicity on the other cell lines, and was also placed separately.

Neutral red incorporation assay

The cell viability (IC50) was determined by using neutral red assay (NR) as previously described [17]. After the cells were treated with the compounds, a neutral red solution (0.33%) was added to the culture medium (1:10, v/v), which was then gently mixed with a multi-channel pipette to avoid crystal formation; subsequently, it was incubated at 37⍰C and 5% CO_2 for not longer than for 2 h. Subsequently, the cells were washed with warm PBS, and 150 ⍰l solubilization solution (50% v/v ethanol, 1% v/v acetic acid in water) was added to each well. The absorbance was measured at 540 nm with a reference wavelength of 630 nm by using a Labsystems Multiskan RC Reader. The background value was the absorbance of the samples with the cell medium incubated with dye, but without cells, and it was subtracted from the absorbance value obtained for the cells. The results were calculated as a percentage of control values obtained for untreated cells.

MTT assay

After the cells were treated with the compounds, they were incubated for 3 h with a pre-warmed solution of MTT in HBSS (0.5 µg/ml, 37°C, and 5% CO_2). Then, the MTT solution was thoroughly removed, the cells were washed in HBSS, and 150 µl DMSO was added for formazan solubilization (5–10 min, shaking at 600 × *g* at ambient temperature in the dark, 20–22°C). The absorbance at 595 nm (test wavelength) and 630 nm (reference wavelength) was measured using a microplate reader (Labsystems Multiskan RC reader, Vantaa, Finland). The results were calculated as a percentage of control values obtained for untreated cells. MTT assay is based on formazan production. Our data obtained for all nucleoside conjugates with metallacarboranes and unmodified metallacarboranes showed that the compounds caused an overproduction of formazan inside the cell regardless influence on cell viability, evaluated by ATP or NR assays. The results of MTT test were therefore inconsistent with other viability assays (data not shown).

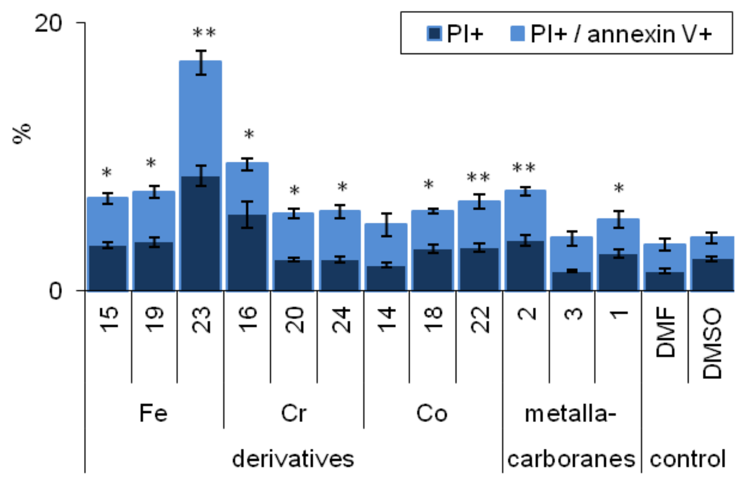

Figure S1. Effect of compounds **1**–**24** (40 μM) on the necrosis and late apoptosis of A2780cis cells. DMF or DMSO (<0.04%, not toxic, not effective) was used as the control. Data were expressed as percentage of cells in necrosis (PI+, annexin V-) and late apoptosis (annexin V+/ PI+) stages; the mean ± SEM of three – six experiments was shown; * p <0.05; ** p <0.01, compound *vs.* control (DMF), total PI-positive cell count. The results for PI staining and NR viability assay were consistent.

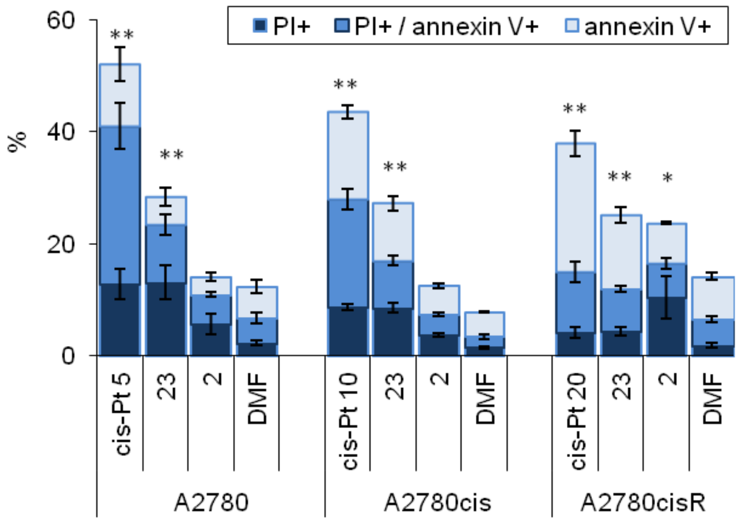

Figure S2. Effect of compounds **23**, **2**, and cisplatin on apoptosis and necrosis in A2780 sublines, A2780, A2780cis, and A2780cisR. DMF (<0.04%, not toxic, not effective) was used as the control. Data were expressed as cumulative percentage of apoptotic and necrotic cells at different stages: necrosis (PI+, annexin V-), late (annexin V+/ PI+) and early apoptosis (annexin V+/ PI-); the mean ± SEM of three – six experiments was shown; * p <0.05; ** p <0.01, compound *vs.* control (DMF); p values were shown for total PI-positive cell count. The results for PI staining and NR viability assay were consistent.

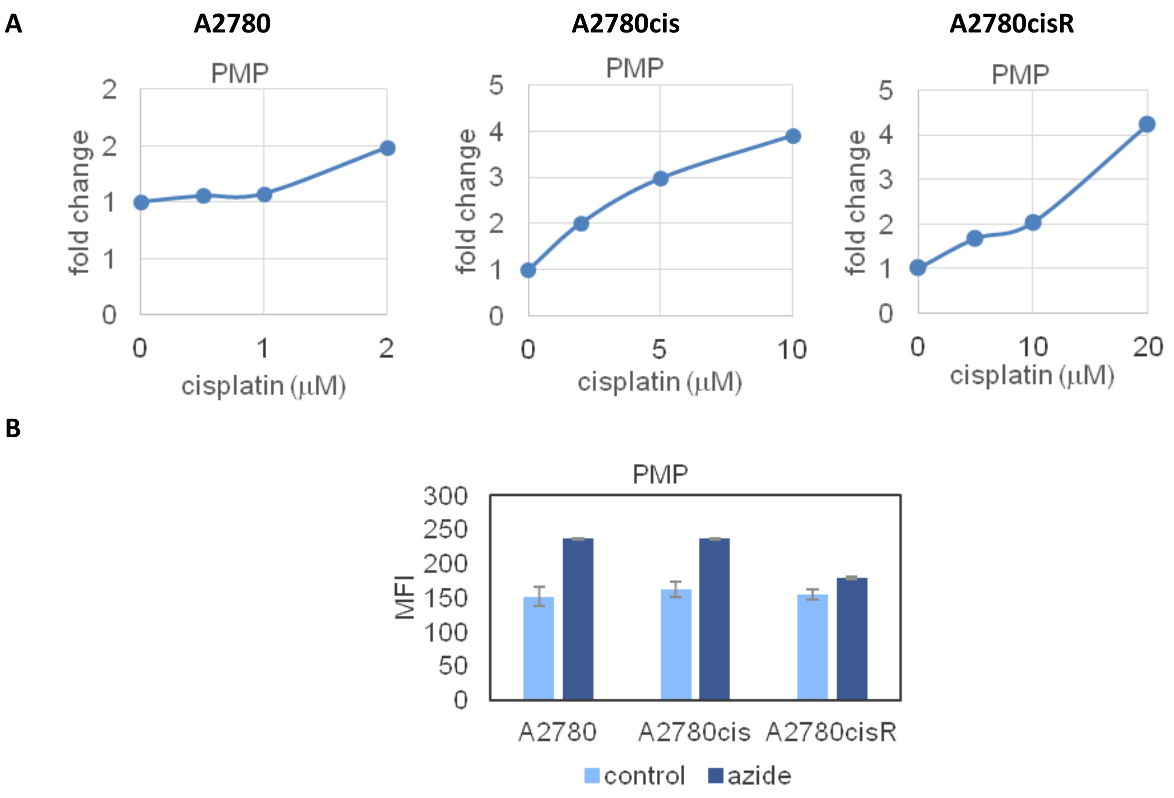

Figure S3. Effect of increasing doses of cisplatin on plasma membrane potential in A2780, A2780cis, and A2780cisR cells (A); (B) the reference experiment demonstrating decrease in plasma membrane potential in A2780cis cells in response to toxic effect of sodium azide.

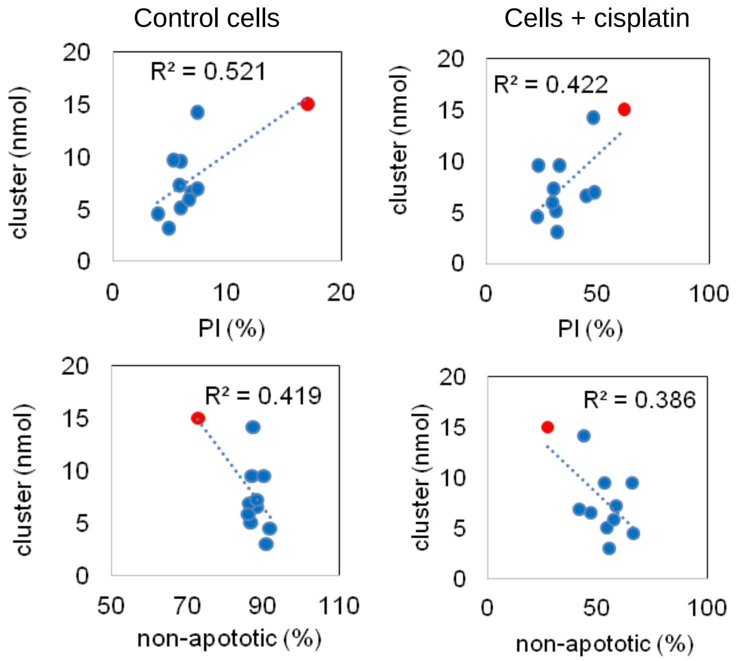

Figure S4. Relationships of incorporation of metallacarboranes unmodified or adenosine derivatives into A2780cis cells to cell viability (non-apoptotic cells, %) and necrosis (PI, %). Incorporation of boron-bearing compounds into cells was determined using ICP-MS method; boron content was expressed as nmoles of Fe-, Cr-, or Co-containing metallacaborane cluster ($Me[C_2B_9H_{11}]_2$) per 10^6 cells; red dots – compound **23**.

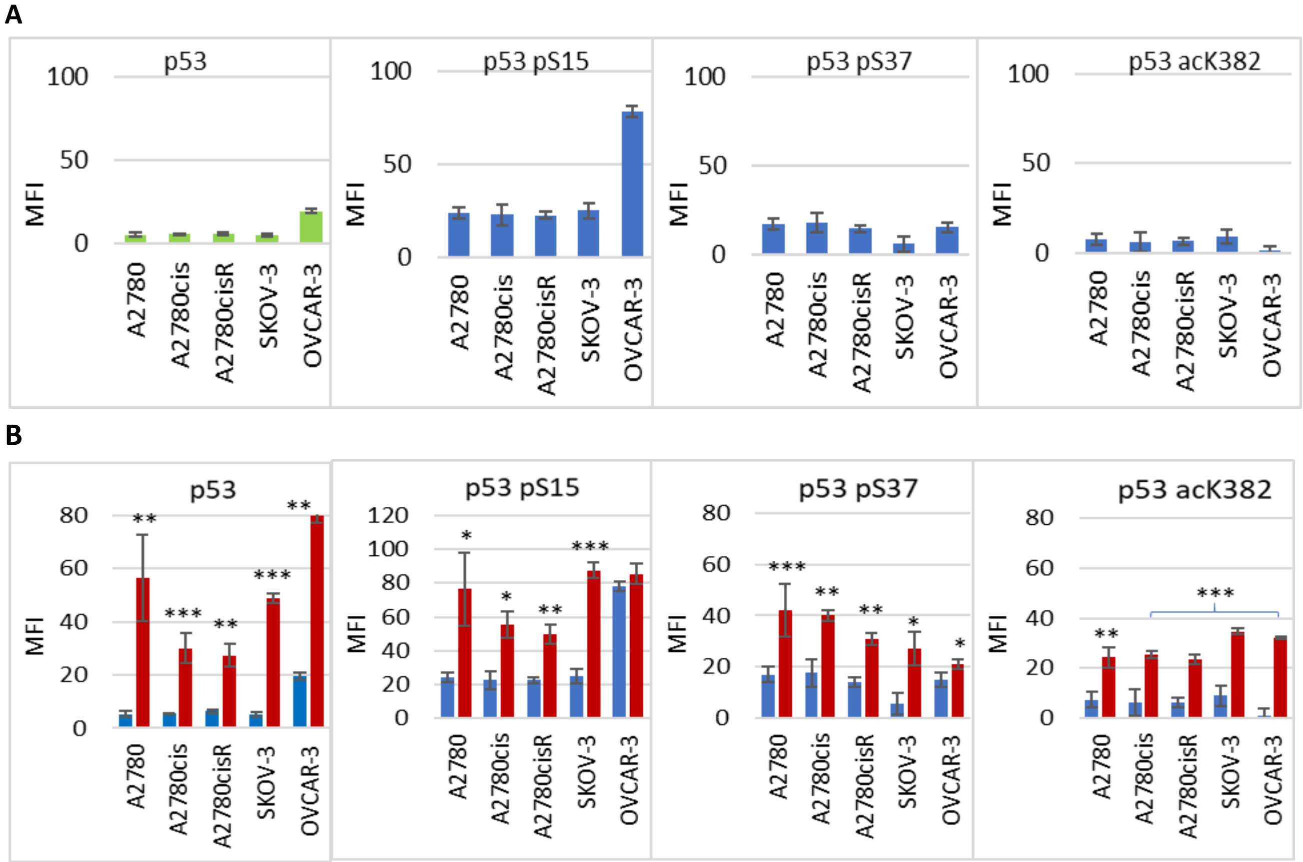

Figure S5. Expression levels of tumor suppressor protein p53 and its phosphorylated and acetylated forms in ovarian cancer cell lines A2780, A2780cis, A2780cisR, SKOV-3, and OVCAR-3; basal expression level and after treatment with cisplatin. The expression of p53 was measured using intracellular labeling method with specific antibodies for human wild type and mutant p53, clone DO-7 (p53); p53 phosphorylated at Ser15 (p53 pS15); p53 phosphorylated at Ser37 (p53 pS37); and p53 acetylated in the C-terminal region at Lys382 (p53 acK382). Intracellular labeling was performed as described in Material and Methods. Data are expressed in relative fluorescence units (MFI, median fluorescence intensity) as means ± SEM values calculated for three – four independent experiments.

A) Basal expression levels. B) Effect of cisplatin, red bars. Cells were incubated for 24 h with cisplatin (5 μM for A2780 and OVCAR-3, 20 μM for A2780cis, A2780cisR, and SKOV-3). DMF was used as the control (0.01% or 0.05%, not toxic, not effective on p53 expression level). *P < 0.05, **p < 0.01, and ***p < 0.001, compounds *versus* control (Student's t-test with Bonferroni correction for multiple comparisons).

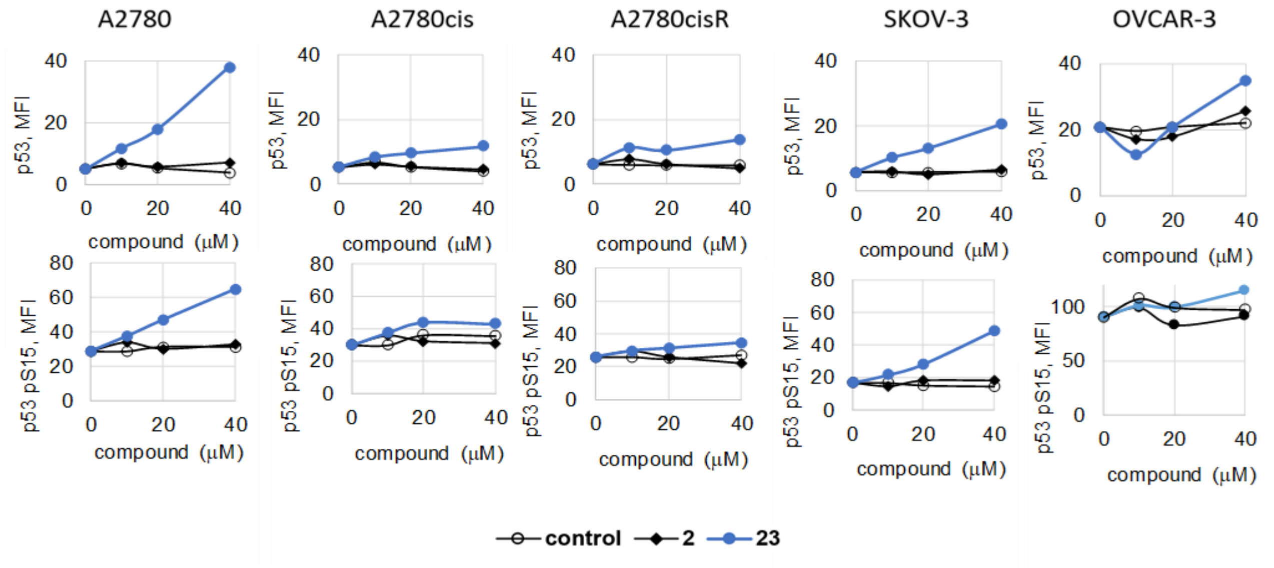

Figure S6. Dose-dependent increase in expression of p53 (upper panel) and p53 p53(pS15) (lower panel) in ovarian cancer cells, stimulated with compound **23**, in comparison to treatment with compound **2**. Cells were incubated with compounds **23** or **2** (10, 20 and 40 µM) for 24 h. Intracellular labelling was performed in PFA-fixed cells in the presence of using FITC anti-p53 mAb (clone DO-7, BD Bioscience) and anti-p53 pS15-APC mAb (clone REA825, Miltenyi Biotec) followed by flow cytometry measurement, as described in Material and Methods. Data are expressed in relative fluorescence units (MFI, median fluorescence intensity).

Table S1. Effect of compounds **23** and **2** on p53 expression expressed as fold change (treated/control).

A

	p53	pS15	pS37	AcK383
A2780	7.9	2.9	2.7	4.3
A2780cis	3.5	1.9	1.9	4.6
Acis2780R	3.1	1.7	1.9	5.1
SKOV-3	4.6	2.5	5.8	3.9
OVCAR-2	3.4	1.7	2.2	75.3

B

	p53	pS15	pS37	AcK383
A2780	1.7	1.7	1.6	0.8
A2780cis	0.9	1.2	0.7	1.3
Acis2780R	0.7	1.2	1.1	1.2
SKOV-3	1.2	1.1	1.7	1.0
OVCAR-2	1.2	1.0	1.0	12.7

Cells were incubated for 24 h with compounds **23** (A) or **2** (B) at concentration of 40 µM for 24h. Expression levels of p53, p53 phosphorylated at Ser15 (pS15) and Ser37 (pS37), or acetylated at Lys382 (AcK382) were determined by intracellular staining as described in Materials and Methods. The mean of four independent experiments are shown. High relative values for AcK382 in OVCAR-3 cells are due to marginal baseline.

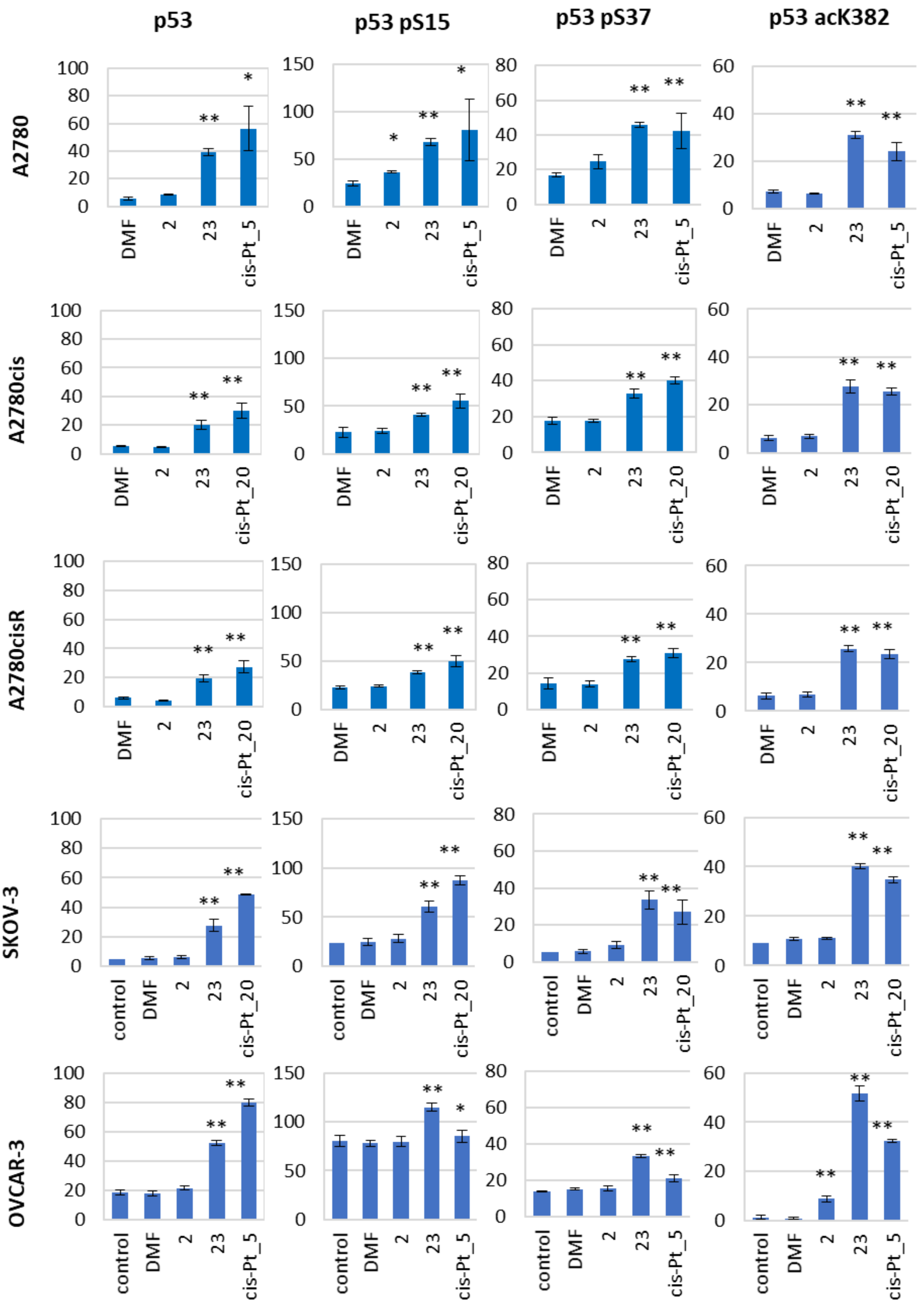

Figure S7. Effect of 2’ derivative of adenosine modified with this metallacarborane (compound **23**) and Fe-containing metallacarborane (compound **2**) on the expression levels of p53 and its phosphorylated forms (p53 pS15 and p53 pS37) in various cell lines of ovarian cancer. The results for cisplatin are shown for comparison as a positive control for p53 activation by a pro-apoptotic inducer. Cells were incubated for 24 h with compounds **2** or **23** (40 μM), or cisplatin (5 μM, or 20 μM, for both cisplatin-sensitive and cisplatin-resistant cell lines, respectively). DMF was used as the control (0.01%, 0.02%, or 0.04%, not toxic, not effective on p53 expression level). Intracellular labelling was performed in PFA-fixed cells in the presence of using specific antibodies, as described in Material and Methods. Data are expressed in relative fluorescence units (MFI, median fluorescence intensity) and expressed as means ± SEM values calculated for three – four independent experiments. *P <0.05, and **p <0.01, compounds *vs.* control (Student’s t-test with Bonferroni correction for multiple comparisons).

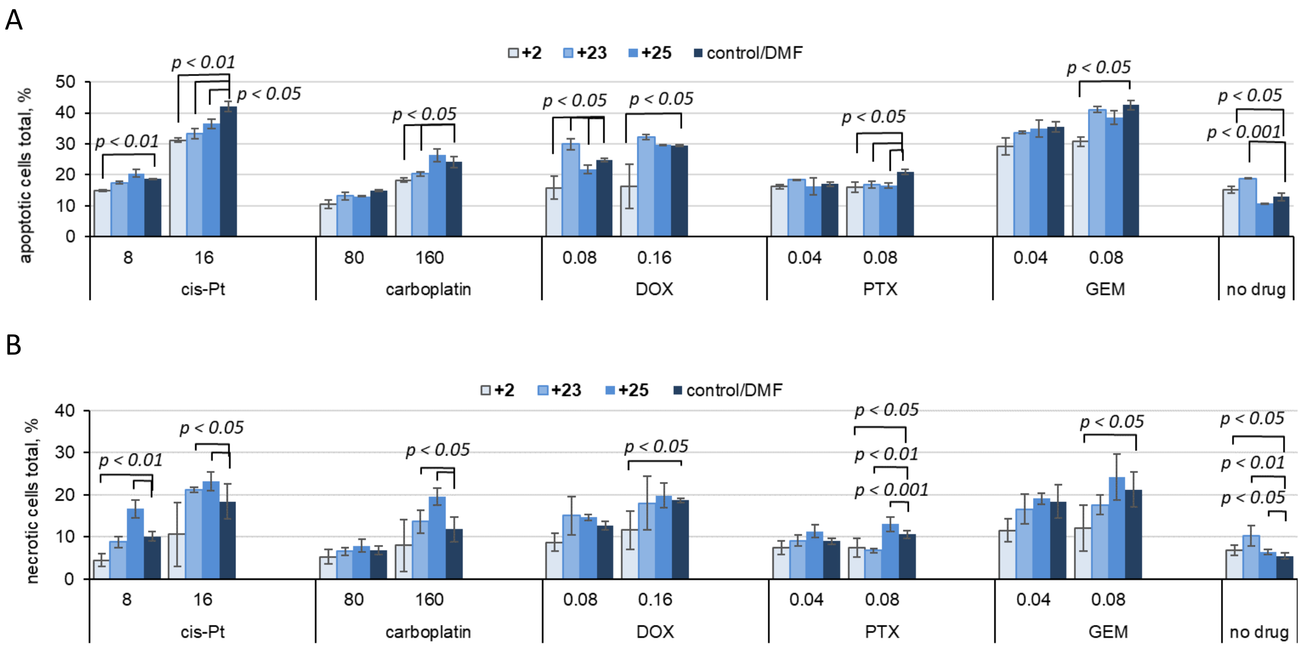

Figure S8. Apoptosis (A) and necrosis (B) of A2780cisR cells cultured for long term in medium with addition of compounds **2**, **23**, or **25** exposed to cisplatin (cis-Pt), carboplatin, DOX, PTX or GEM. The cell cultures grew in the basal medium (control), medium with addition of vehicle DMF 0.02% (DMF), or compounds **2**, **23**, and **25** at a concentration of 20 μM for a period of 16 days (see Materials and Methods). Next, cells were treated 24h with cisplatin (8 and 16 μM), carboplatin (80 and 160 μM), DOX (0.08 and 0.16 μM), PTX or GEM (0.04 and 0.08 μM). Cell apoptosis (A) and necrosis (B) was determined by flow cytometry by PI and annexin V staining, respectively. Data are expressed as percentage of PI or Annexin V positive cells. The mean ± SEM of three experiments are shown. *P* values were calculated by Student t-test, cells cultured with compound *vs.* without compound.

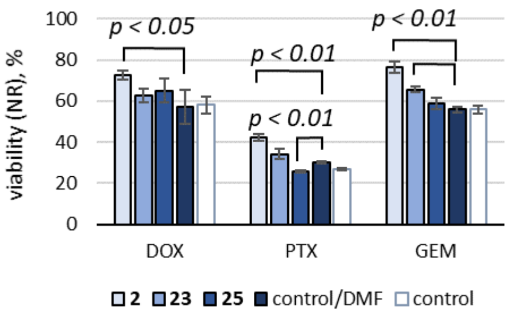

Figure S9. Viability of cells cultured in different conditions (i.e. with compounds **2**, **23**, or **25**, or without compounds (control and control/DMF)), and next treated for 48h with DOX (0.16 μM), PTX and GEM (0.08 μM); data for neutral red (NR) assay were expressed as percentage values of control cells not treated with drugs (100%). The mean ± SEM of three experiments were shown; p values calculated for comparison: cells cultured with compound *vs.* without compound; Student t-test with Bonferroni correction.

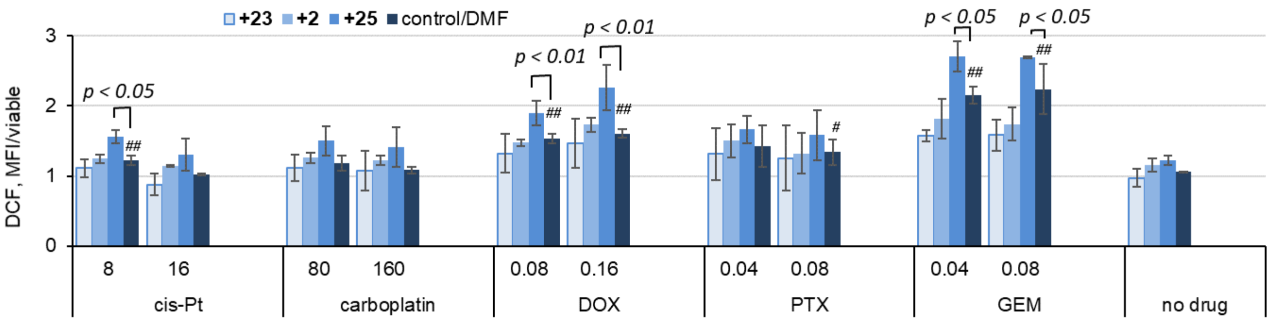

Figure S10. ROS production in A2780cisR cells cultured for long term with the presence of 2’ adenosine derivative modified with this metallacarborane (compound **23**) or with metallacarborane containing Fe ion (compound **2**) or with *nido*-carborane (compound **25**). Data were normalized to the percentage of viable cells. A2780cisR cells were cultured for long term (as described in Materials and Methods) with the presence compounds **2**, **23**, or **25**. Next, cells were treated 24h with cisplatin, carboplatin, DOX, PTX or GEM (at IC50 and ½ × IC50). Intracellular ROS production was measured by flow cytometry using TMRE assays (MFI, median intensity of fluorescence). MFI values were normalized to the percentage of live cells, PI- (MFI/viable cells). The mean ± SEM of three experiments are shown. *P* values were calculated by Student t-test, cells cultured with compound *vs.* without compound; ; #p < 0.05, ##p < 0.01 drug *vs.* no drug.

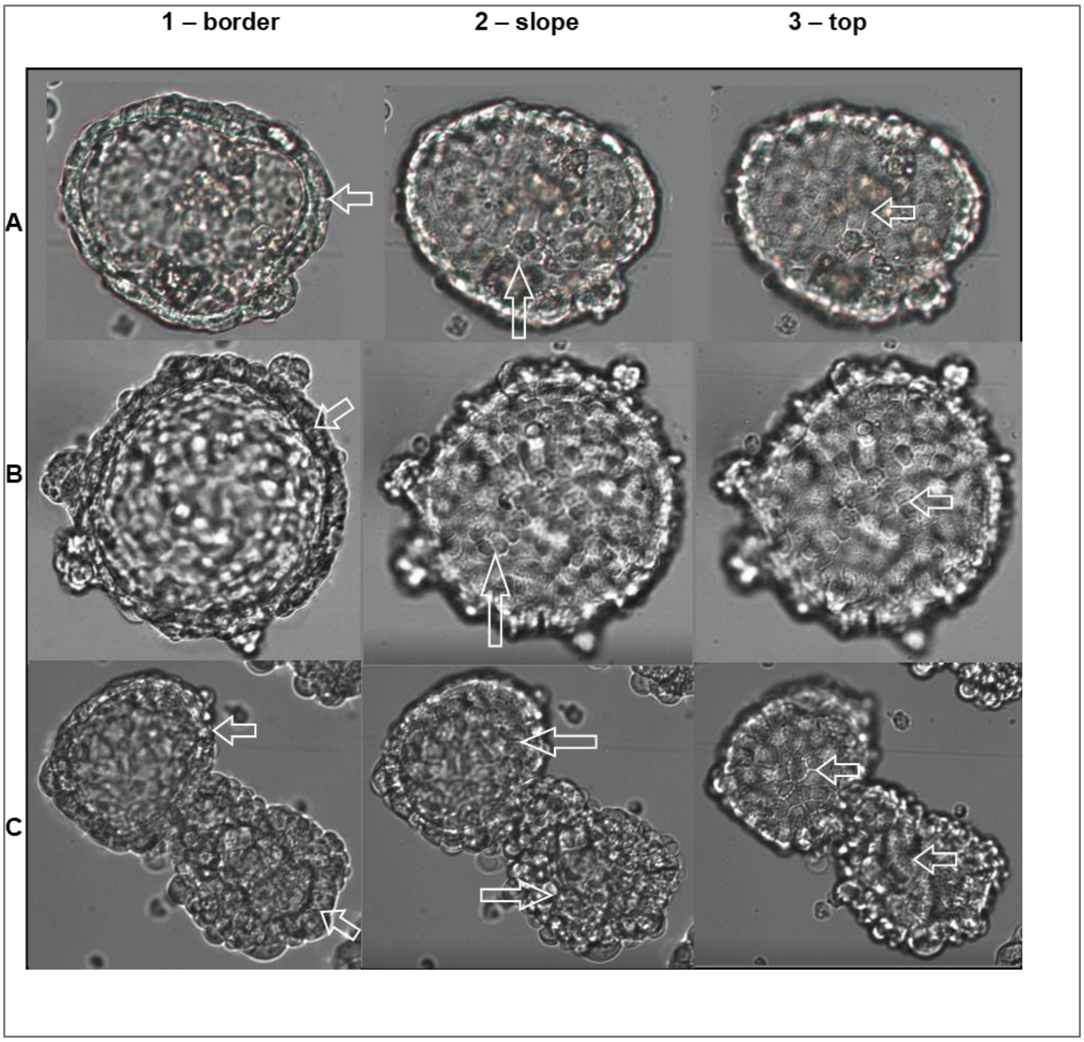

Figure S11. Differential interference contrast (DIC) image of the spheroids formed by OVCAR-3 cells focused on the following areas of the spheroid, along the z–axis: boundary, slope and top. OVCAR-3 cells form compact spheroids of various sizes (A-C).

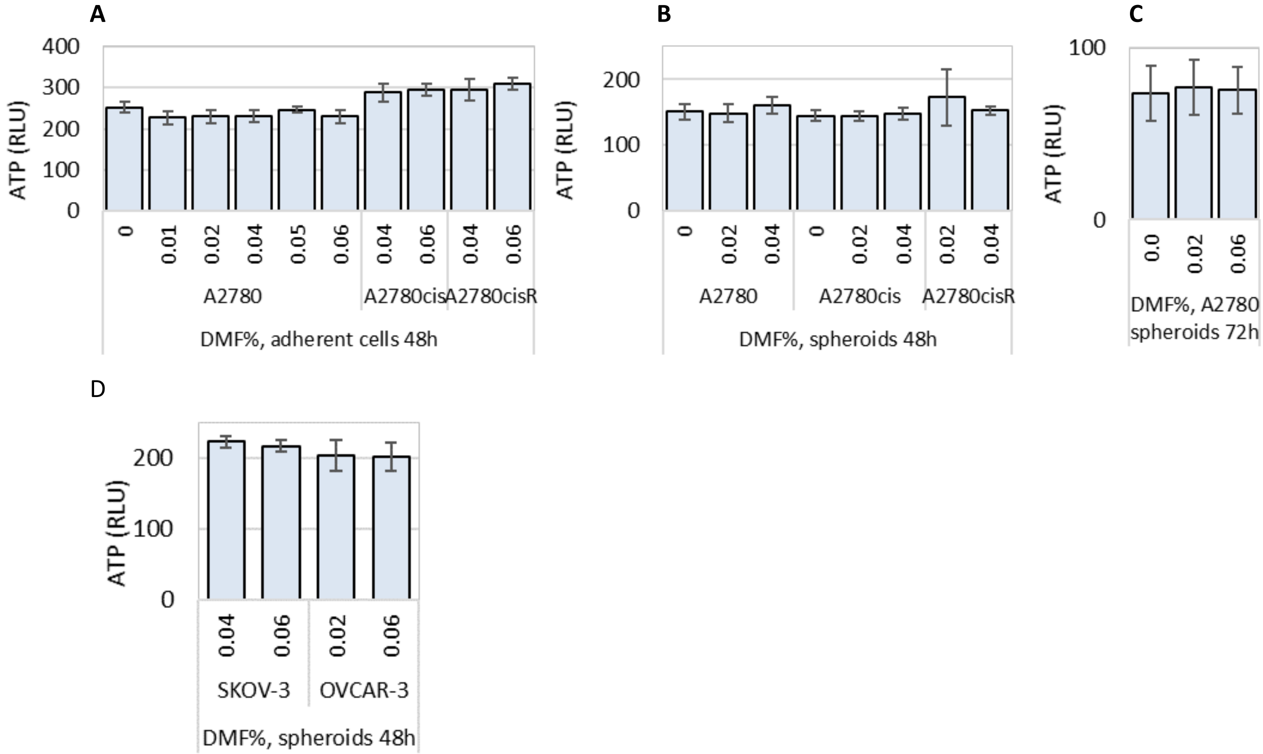

Figure S12. The effect of solvent (DMF) on the viability of spheroids and adherent cells cultured for 48 h (37°C and 5% CO2 in humidified atmosphere) at concentrations equal to DMF content in the samples incubated with the compounds (**23** and cisplatin alone, or in combination). Control cells without compound treatment were cultured with a solvent in amounts corresponding to that of the treated samples. The viability of the cells was determined by using a CellTiter 96 3D Viability Assay (Promega, Madison, USA), based on the luminescent measurement of ATP. Values are expressed as the relative luminescent units (RLU). The mean ± SEM values from three experiments are presented. (A) Adherent cells of A2780, A2780cis, and A2780R lines; (B) spheroids derived from A2780, A2780cis, and A2780R cell lines; (C) 72 hours cultures of A2780 spheroids for comparison; (D) spheroids derived from SKOV-3 and OVCAR-3 cell lines. DMF was not toxic to the spheroids.

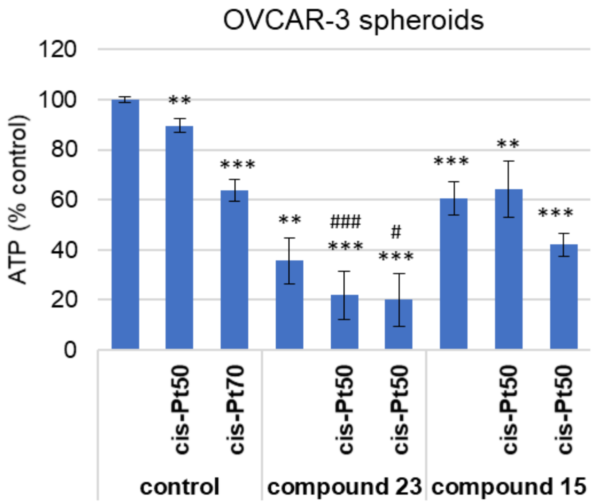

Figure S13. Effect of compounds **15** and **23** alone and in combination with cisplatin on the viability of OVCAR-3 spheroids. Spheroids were incubated with compounds (40 μM) for 3 hours followed by 48 hours of the cisplatin treatment (50 μM and 70 μM). Cell viability was determined using CellTiter-Glo 3D Viability Assay (Promega), based on the luminescent measurement of ATP (RLU – relative luminescent units). DMF (<0.06%, not toxic) was used as the control. Data are expressed as percentage values of the control (untreated cells). The mean ± SEM values were calculated for three-four experiments. *P < 0.05, **p < 0.01, and ***p <0.001, compounds *vs.* control; #p < 0.05 and ##p < 0.001, combined treatment *vs.* cisplatin; Student's t-test, differences significant for multiple comparisons are shown (Bonferroni correction).

II. Chemistry

Crystalography Data

Table S2. Crystal data and structure refinement for compounds **3** and **7**.

	**3**	**7**
**CCDC deposition number**	2042928	2042960
Empirical formula	$C_6H_{33}B_{27}Cr_{1.5}Cs_{1.5}$	$C_{14}H_{35}B_{18}CrO_2$
Formula weight [g·mol^{-1}]	674.56	482.00
T [K]	100.00(10)	100.01(10)
Crystal system	Monoclinic	Monoclinic
Space group	$P2_1/n$	$P2_1/c$
a [Å]	11.4856(4)	15.98920(10)
b [Å]	22.3739(6)	11.02570(10)
c [Å]	11.5971(4)	14.55260(10)
α [°]	90	90
β [°]	116.270(5)	93.3340(10)
γ [°]	90	90
Volume [Å^3]	2672.39(18)	2561.17(3)
Z; calculated density [g·cm^{-3}]	4; 1.677	4; 1.250
Absorption coefficient [mm^{-1}]	2.636	3.738
F(000)	1290.0	996.0
Crystal shape, color	prism, red	plate, clear intense red
Crystal size [mm^3]	0.107 × 0.077 × 0.049	0.078 × 0.049 × 0.025
Radiation	Mo$K\alpha$ (λ = 0.71073)	Cu$K\alpha$ (λ = 1.54184)
2ϑ range for data collection [°]	5.348 to 52.744	5.536 to 140.12
Index ranges	$-14 \le h \le 14$, $-27 \le k \le 27$, $-14 \le l \le 13$	$-19 \le h \le 19$, $-13 \le k \le 13$, $-17 \le l \le 17$
Reflections collected	29885	89944
Independent reflections	5433 [R_{int} = 0.0447, R_{sigma} = 0.0297]	4860 [R_{int} = 0.0519, R_{sigma} = 0.0177]
Data/restraints/parameters	5433/0/445	4860/0/456
Goodness-of-fit on F^2	1.038	1.030
Final R indexes [$I \geq 2\sigma(I)$]	R_1 = 0.0224, wR_2 = 0.0498	R_1 = 0.0351, wR_2 = 0.0941
Final R indexes [all data]	R_1 = 0.0280, wR_2 = 0.0523	R_1 = 0.0364, wR_2 = 0.0952
Largest diff. peak/hole [e.Å^{-3}]	0.49/–0.41	0.55/–0.40

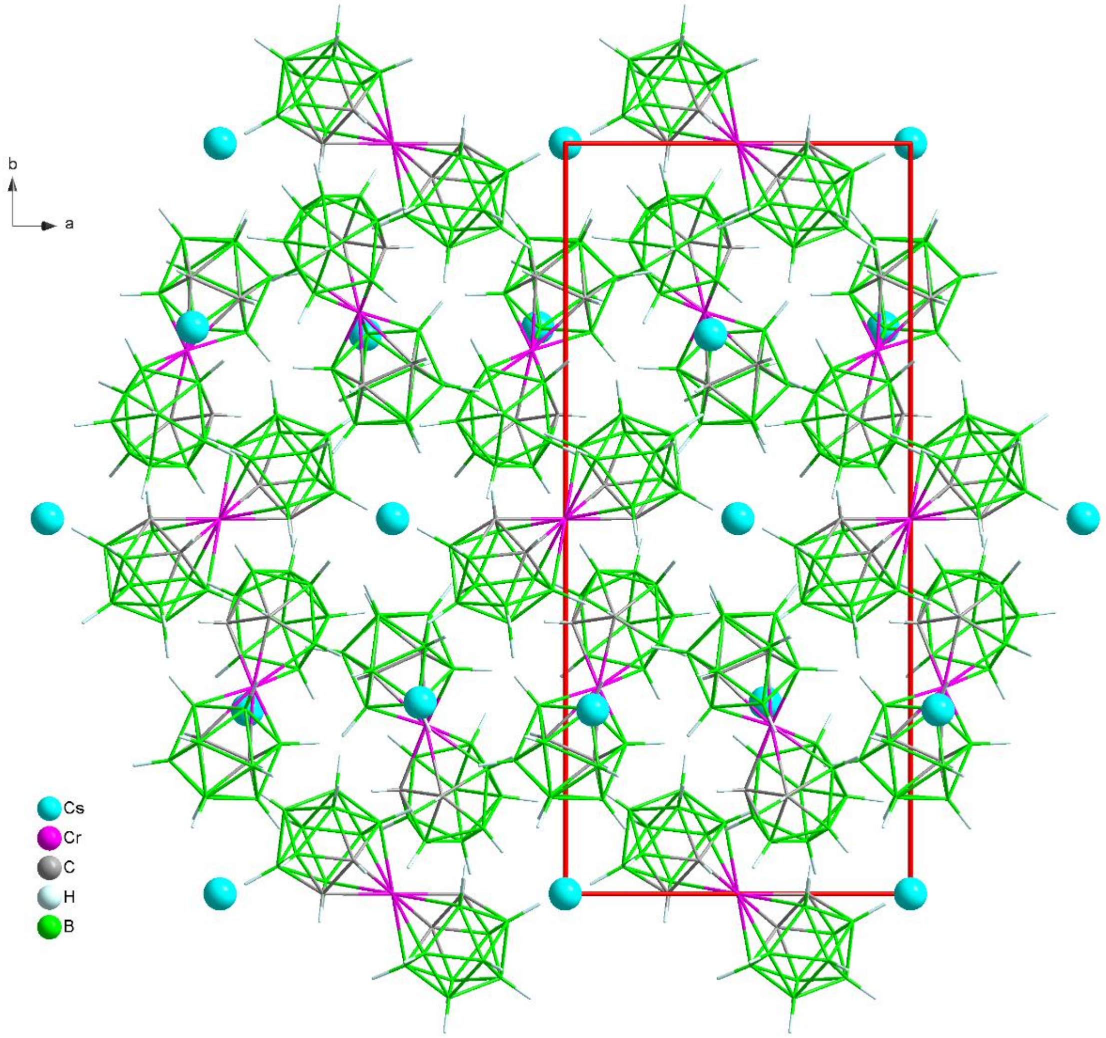

Figure S14. Crystal packing in compound **3** along the *ab* plane.

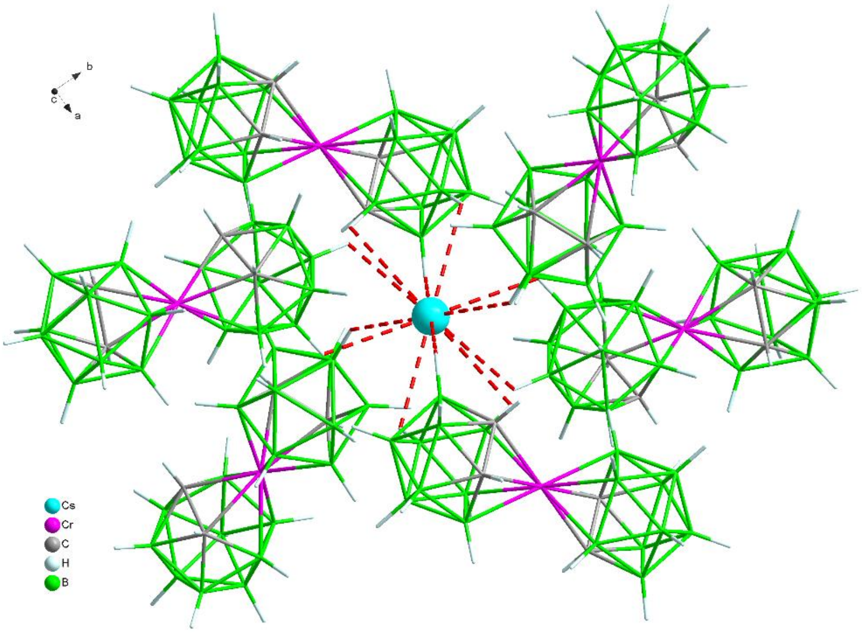

Figure S15. The Cs^{+} cations surrounded by $[3,3'\text{-}Cr(1,2\text{-}C_2B_9H_{11})_2]^{-}$ anions located in the cavities of compound **3**.

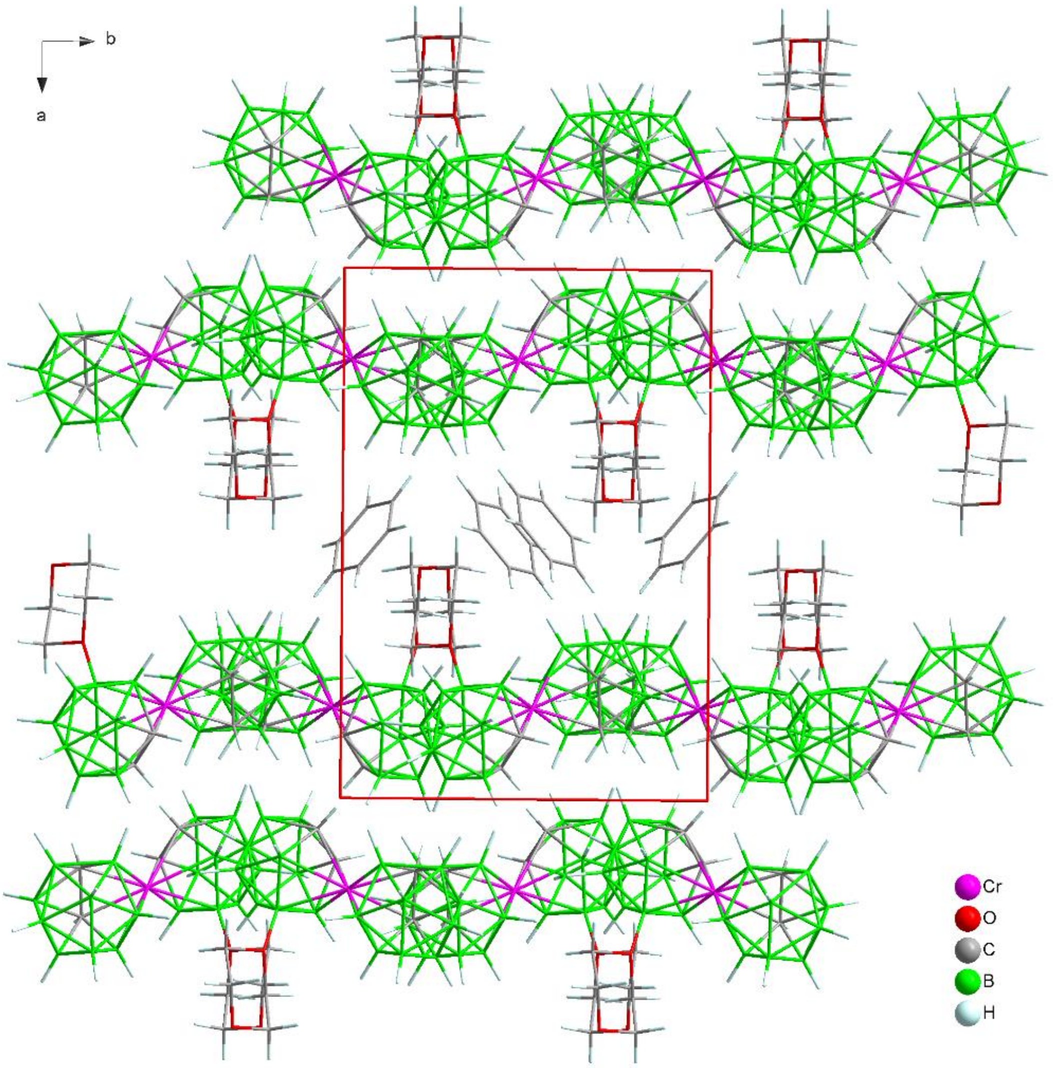

Figure S16. Crystal packing in compound **7** along the *ab* plane.
